# Patterns of breast, prostate and cervical cancer incidence and mortality in Colombia: an administrative registry data analysis

**DOI:** 10.1186/s12885-020-07611-9

**Published:** 2020-11-11

**Authors:** Juliana Alexandra Hernández Vargas, Paula Ximena Ramírez Barbosa, Ana Milena Gil Quijano, Ana María Valbuena, Lizbeth Acuña, Jaime Alberto González

**Affiliations:** 1 Cuenta de Alto Costo, Fondo Colombiano de Enfermedades de Alto Costo, Avenue career 45 number 103-34, Building Logic 2, Office 802, 110111 Bogotá, Colombia; 2 Asociación Colombiana de Hematología y Oncología, Bogotá, Colombia

**Keywords:** Cancer, Epidemiology, Incidence, Mortality, Registries

## Abstract

**Background:**

Cancer is widely recognized as a global public health problem. Breast, prostate, and cervical cancer are among the most frequent types in developing countries. Assessing their incidence and mortality by regions and municipalities is important to guide evidence-based health policy. Our aim was to describe the incidence and mortality trends for breast, cervical, and prostate cancer across regions and municipalities in Colombia during 2018.

**Methods:**

*We performed* a cross-sectional analysis with data from people with breast, prostate, or cervical cancer, reported to the National Administrative Cancer Registry during 2018. A descriptive analysis was performed. Age-standardized incidence and mortality rates were estimated at national, regional, and municipal levels. Finally, we identify the regions and municipalities with significantly higher or lower incidence and mortality rates compared to national estimations.

**Results:**

Breast cancer was the most frequent type among all new cases and deaths in Colombia. Breast, prostate and cervical cancer incidence and mortality rates per 100,000 were: 18.69 (CI 95%: 18.15–19.25) and 10.48 (CI 95%: 10.07–10.91); 11.34 (CI 95%: 10.90–11.78) and 7.58 (CI 95%: 7.22–7.96); 5.93 (CI 95%: 5.62–6.25) and 4.31 (CI 95%: 4.05–4.58), respectively. Eastern region had both, incidence and mortality rates, significantly lower than national for all types of cancer. By municipalities, there was a heterogeneous pattern. Nonetheless, Agua de Dios (Cundinamarca), had one of the highest incidence rates for all types.

**Conclusions:**

We observed clear differences in cancer incidence and mortality across regions and municipalities, depending on each type of cancer. Our findings are important to improve screening coverage, early detection, and treatment in the country.

**Supplementary Information:**

The online version contains supplementary material available at 10.1186/s12885-020-07611-9.

## Background

Cancer is one of the major public health concerns worldwide because its incidence and mortality, as well as its impact on life expectancy across populations, are rapidly increasing. Besides aging, population distribution, and changes in the frequency of the main risk factors for cancer can explain this trend and heterogeneity between regions, especially in countries with low socioeconomic development [1]. According to the Global Cancer Observatory (GLOBOCAN), in 2018, there were 18.1 million new cases and 9.6 million cancer deaths worldwide. For both sexes, lung cancer was the most frequent type with 2.1 million and 1.8 million incident cases and deaths, respectively. Global 5-year prevalent cases were 43.8 million [2].

Regarding region, Asia concentrates the highest number of new cases (8.8 million) and deaths (5.5 million), while Latin American and the Caribbean (LAC) is in the fourth place with 7.8% of incidence (1.4 million cases) and 7.1% (672 thousand deaths) of mortality worldwide [2]. In terms of the burden of the disease, cancer caused 233.5 million DALYs (Disability-Adjusted Life-Years) in 2017, of which 97% came from YLLs (Disability-Adjusted Life-Years) and 3% from YLDs (Years Lived with Disability) [3].

Globally, there were more new cases and deaths in men (9.5 and 5.4 million, respectively) than women (8.6 and 4.2 million, respectively). Lung cancer accounts for 14.5 and 22.0% of incidence and mortality in men. Further, 13.5 and 10.9% of new cases were diagnosed with prostate and colorectal cancer. Otherwise, in women, the most common incident cancers were breast (24.2%), colorectal (9.5%), and lung (8.4%), and breast and lung cancer were the leading cause of cancer death (15.0 and 13.8%, respectively) [2].

Concerning mortality, despite it has been declined in most higher-income countries, this progress has been deficient in low- and middle-income countries, where the Sustainable Development Goals have not been reached. It is well known that cancer is a complex disease with mortality patterns which vary importantly across countries and specific types of cancer. Variations depend on differences in lifestyles, such as smoking, and structural conditions of the health systems of each country. In most higher-income countries, mortality rates have decreased mainly due to interventions focused on screening, early prevention, and timely diagnosis while in countries in transition, such us Colombia they are rising or stable for many types, including breast, prostate, and colorectal cancer [4].

As we mentioned, cancer distribution by region is heterogeneous. In fact, in LAC, breast (14.1%) and prostate cancer (13.5%) were the most common types among new cases. Prostate and breast cancer grouped 27.9 and 27.4% of incidence in men and women, respectively [2]. This pattern is similar for all countries in the region, including Colombia, due to the ongoing sociodemographic transitions and health care conditions: advanced stage at diagnosis and limited access to diagnosis and treatment strategies [5]. Morbidity and mortality trends within countries are similar and their analysis is important for health care planning to reduce disparities in cancer distribution and impact on populations.

Therefore, we aim to describe the incidence and mortality trends for breast, cervix uteri and prostate cancer across regions and municipalities in Colombia during 2018.

## Methods

### Data sources

A cross-sectional analysis was performed on data compiled by the National Administrative Cancer Registry (NACR) administered by the High-Cost Diseases Fund (CAC-in its Spanish acronym) from January 2nd, 2018 to January 1st, 2019. The NACR was created by the Ministry of Health of Colombia in 2012 [6] and its goal is to collect and analyze demographic, clinical, and administrative information on people with cancer across the country through the annual report of 134 variables. Taking into account that 98% of the Colombian population is insured to the national health system and must be reported to the NACR by its health insurers, it can provide reliable information about real-life patterns and trends of the most common cancer types in Colombia. Since the first measurement in 2015, 279,155 people have been reported with cancer. Unique identifiers have been created for identifying and protecting the personal information of the participants. Data on prevalent cases are updated every year, while for new cases, full registration is completed. There is a well-established data monitoring process to guarantee the quality of information, which is carried out in two steps: a prior identification of mistakes in the reporting process through a systematized algorithm. Then, the information reported is auditing and compared with health clinical records to ensure their accuracy for all new diagnoses reported.

### Eligibility of participants

The analysis was performed with information from people with breast, prostate, or cervix uteri cancer reported during the study period. In case of breast cancer, we restricted the analysis to women.

### Cancer incidence data

Incident cases were defined as people with a primary breast, prostate, or cervix uteri tumor, diagnosed within the analysis time frame and reported by the first time to the NAC*R. cancer* diagnosis could be clinical or histopathological. The diagnosis was confirmed in medical records through a data monitoring process. The anatomic site and histology were coded according to the International Classification of Diseases 10th edition (ICD-10) and the International Classification of Diseases for Oncology third edition (ICD-O-3). For the types of cancer analyzed, codes were defined as follows: breast (ICD-10: C50 to D50; ICD-O-3: C50), cervix uteri (ICD-10: C53 to D06; ICD-O-3: C53), and prostate (ICD-10: C61; ICD-O-3: C61).

### Cancer mortality data

Information about deaths was reported to the CAC by health insurers. It was verified with external sources provided by the Ministry of Health and the National Registry of Civil Status. Deaths for any cause were considered to estimate mortality rates.

### Demographic and clinic data

Demographic information included age, sex, region, and the municipality of residence and health insurance. Regarding region, they were defined by the Department for National Statistics (DANE, by its acronym in Spanish), from Colombia’s 32 departments according to the gross domestic product, identifying 6 regions: Bogotá D. C, Central, Eastern, Pacific, Caribbean and Other departments (Figure S1 of Supplementary Material). Municipalities were defined as autonomous territories, at a lower level than departments and their classification was also provided by the DANE. About health insurance, there are four regimes (contributory, subsidized, special, and exception) and a minimum proportion is uninsured. In respect of clinical data, invasive cancer was defined following IARC criteria based on ICD-10 [7]. The staging was determined depending on the type of cancer, as follows: for breast and prostate cancer the Tumor-Node-Metastasis (TNM) classification was used based on the eighth edition of the American Joint Committee on Cancer (AJCC) [8, 9] and for cervical cancer, the staging was based on the revised 2018 International Federation of Gynecology and Obstetrics (FIGO) system [10]. Patient care opportunity was calculated as the number of days between the clinical suspicion and the confirmed diagnosis (date of pathology report or clinical diagnosis) and, between the diagnosis and the first treatment (systemic therapy, radiotherapy, or surgery).

### Statistical analysis

We summarized continuous variables as medians and interquartile range (IQR) and categorical variables as absolute values and percentages. Incidence and mortality rates were calculated using the mid-year population projected by the DANE (national, regional, or municipal) for the calendar year of interest. Rates by region or municipality were age-standardized (ASR) to the Colombian population estimated by the DANE with a cut-off date on June 30th, 2018 (*n* = 49,834,240). In the case of breast and cervix uteri cancer, we restricted the population to women only (*n* = 25,228,444) and, for prostate cancer we used the male population (*n* = 24,605,796). National estimations were standardized using the LAC population estimated by the United Nations for 2019 [11]. ASR were calculated including only invasive cases. Also, ASR and their 95% confidence intervals were estimated by region and municipalities and were expressed per 100,000 people. For each type of cancer, municipalities whose incidence and mortality were significantly higher than national estimations were highlighted in the maps. The statistical analyses were performed in Stata version 13 (StataCorp LP, College Station, Texas, USA), and QGIS version 3.12.2 was used to create the maps.

## Results

### National incidence and mortality rates for each type of cancer

Breast cancer was the most frequent type among all incident cases and deaths with 16.31% (*n* = 4506) and 13.82% (*n* = 2454), respectively. Age-standardized incidence and mortality rates for breast, prostate, and cervical cancer were, in their order: 18.69 (CI 95%: 18.15–19.25) and 10.48 (CI 95%: 10.07–10.91); 11.34 (CI 95%: 10.90–11.78) and 7.58 (CI 95%: 7.22–7.96); 5.93 (CI 95%: 5.62–6.25) and 4.31 (CI 95%: 4.05–4.58) per 100,000 people.

### Demographic and clinic characteristics of incident cases (breast, prostate and cervical cancer)

Table 1 shows demographic and clinical information new cases of breast, cervical, and prostate cancer. Women diagnosed with cervical cancer were younger than those with breast cancer. The highest proportion of people diagnosed with invasive cancer was found in prostate cancer, while the lowest in cervical cancer. Regarding regions, Central and Bogotá D.C. had the greatest number of new cases. The contributory insurance had more than 67.00% of incident cases of breast and prostate cancer, while cervical cancer was more frequent in the subsidized. Most new cases of cervical cancer were in situ, while breast and prostate cancer were mainly diagnosed in stage II. Consistently with the above, surgery was more common in cervical cancer, while systemic therapy was the most indicated treatment in breast and prostate cancer. In terms of care opportunity, time to diagnosis was less for cervical cancer, whereas that, time until the first treatment was shorter for breast cancer.

**Table 1:** Demographic and clinic characteristics of new cases of breast, prostate and cervical cancer in Colombia 2018^a^

Variables	Type of cancer		
	Breast (***n*** = 4855)	Prostate (***n*** = 2617)	Cervical (***n*** = 1930)
*Age, years*^b^	57 (47–66)	68 (62–74)	47 (37–59)
*Age categories, years*			
0–9	0 (0.00)	1 (0.04)	0 (0.00)
10–19	1 (0.02)	0 (0.00)	1 (0.05)
20–29	75 (1.54)	0 (0.00)	125 (6.48)
30–39	389 (8.01)	0 (0.00)	497 (25.75)
40–49	1004 (20.68)	42 (1.60)	474 (24.56)
50–59	1349 (27.79)	355 (13.57)	377 (19.53)
60–69	1216 (25.05)	1074 (41.04)	257 (13.32)
70–79	592 (12.19)	876 (33.47)	141 (7.31)
80+	229 (4.72)	269 (10.28)	58 (3.01)
*Region*			
Central	1522 (31.35)	776 (29.65)	513 (26.58)
Bogotá D.C.	1104 (22.74)	654 (24.99)	400 (20.73)
Pacific	810 (16.68)	462 (17.65)	377 (19.53)
Caribbean	827 (17.03)	411 (15.71)	356 (18.45)
Eastern	544 (11.20)	295 (11.27)	217 (11.24)
Other departments	48 (0.99)	19 (0.73)	67 (3.47)
*Health insurance*			
Contributory	3265 (67.25)	1768 (67.56)	857 (44.40)
Subsidized	1351 (27.83)	663 (25.33)	1025 (53.11)
Exception	178 (3.67)	96 (3.67)	32 (1.66)
Special	52 (1.07)	88 (3.36)	12 (0.62)
Uninsured	9 (0.19)	2 (0.08)	4 (0.21)
*Invasive cancer (yes)*	4506 (92.81)	2593 (99.08)	1425 (73.83)
*Staging (yes)*	4503 (92.75)	1903 (72.72)	1814 (93.99)
*Clinical stage*			
In situ	419 (8.63)	42 (1.60)	535 (27.72)
Stage I	904 (18.62)	514 (19.64)	434 (22.49)
Stage II	1759 (36.23)	773 (29.54)	366 (18.96)
Stage III	1148 (23.65)	190 (7.26)	392 (20.31)
Stage IV	273 (5.62)	384 (14.67)	87 (4.51)
No data	352 (7.25)	714 (27.28)	116 (6.01)
*Treatment*			
Systemic therapy	3112 (64.10)	926 (35.38)	614 (31.81)
Radiotherapy	714 (14.71)	582 (22.24)	603 (31.24)
Surgery	2033 (41.87)	751 (28.70)	636 (32.95)
*Care opportunity*^b^			
Clinical suspicion to diagnosis	35 (18–68)	39 (20–90)	34 (15–76)
Diagnosis to treatment	59 (36–91)	71 (35–114)	71 (42–105)

^a^Includes invasive and in situ tumors. Data are presented as absolute values (proportions), unless otherwise specified

^b^Reported values are medians (interquartile ranges)

### Incidence and mortality rates by regions

Table 2 shows incidence and mortality rates of breast, prostate, and cervical cancer by regions. Compared with national estimations, the incidence of breast cancer was significantly higher in Bogotá D.C., and Central region, while Caribbean, Eastern and Other departments regions had incidence rates significantly lower. The incidence of prostate cancer was significantly higher in Bogotá D.C. but significantly lower in the Caribbean, Eastern, and Other departments regions. Finally, cervical cancer incidence was significantly higher in Other departments while it was lower in the Eastern region. Regarding mortality, its distribution across the regions was more heterogeneous than the incidence. Breast cancer mortality rates were significantly lower in Eastern, and Other departments, compared to national. In the case of prostate cancer, they were significantly lower in the Caribbean, Eastern and Other departments, whereas for cervical cancer, the lowest ASR were observed in Bogotá D.C. and Eastern region.

**Table 2:** Incidence and mortality rates for breast, prostate and cervical cancer by regions, Colombia 2018^a^

Region	Type of cancer					
	Breast		Prostate		Cervical	
	n	ASR (95% CI)	n	ASR (95% CI)	n	ASR (95% CI)
**Incidence**						
Bogotá, D.C.	1012	21.59 (20.28–22.97)*	648	15.56 (14.38–16.80)*	267	5.73 (5.06–6.46)
Caribbean	797	16.69 (15.55–17.89) ^¥^	404	8.38 (7.58–9.24) ^¥^	312	6.48 (5.78–7.24)
Central	1385	21.02 (19.93–22.16)*	768	11.73 (10.91–12.59)	382	5.95 (5.36–6.57)
Eastern	511	11.84 (10.84–12.91) ^¥^	293	6.66 (5.92–7.47) ^¥^	155	3.61 (3.07–4.23) ^¥^
Pacific	757	17.44 (16.22–18.72)	461	10.93 (9.96–11.98)	255	5.86 (5.17–6.63)
Other departments	44	8.27 (5.98–11.11) ^¥^	19	3.72 (2.23–5.76) ^¥^	54	9.99 (7.48–13.04)*
**National**	**4506**	**18.69 (18.15–19.25)**	**2593**	**11.34 (10.90–11.78)**	**1425**	**5.93 (5.62–6.25)**
**Mortality**						
Bogotá, D.C.	442	9.71 (8.83–10.67)	292	7.98 (7.09–8.95)	160	3.44 (2.93–4.02) ^¥^
Caribbean	494	10.49 (9.59–11.46)	329	6.70 (6.00–7.47) ^¥^	266	5.59 (4.94–6.30)*
Central	700	10.48 (9.72–11.29)	457	6.99 (6.36–7.66)	265	4.03 (3.56–4.54)
Eastern	332	7.59 (6.80–8.46) ^¥^	234	5.13 (4.49–5.83) ^¥^	130	2.99 (2.50–3.55) ^¥^
Pacific	466	10.64 (9.69–11.65)	318	7.29 (6.51–8.14)	176	4.02 (3.45–4.66)
Other departments	20	3.77 (2.28–5.82) ^¥^	11	2.18 (1.09–3.84) ^¥^	24	4.68 (2.98–6.96)
**National**	**2454**	**10.48 (10.07–10.91)**	**1641**	**7.58 (7.22–7.96)**	**1021**	**4.31 (4.05–4.58)**

^a^All rates are age-standardized per 100.000 population. They were estimated including only invasive cases

*ASR significantly higher than national estimations. *p*-value < 0,05 in a test of proportions

^¥^ASR significantly lower than national estimations. *p*-value < 0,05 in a test of proportions

### Incidence and mortality rates by municipalities

**Fig. 1:**
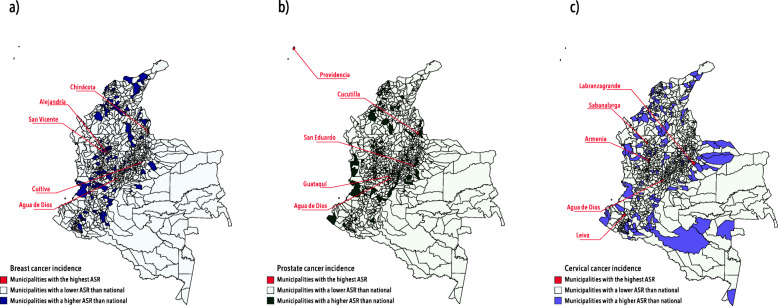
Age-standardized incidence rates of breast, prostate and cervical cancer by municipalities, Colombia 2018. Figure 1 shows the age-standardized incidence rates for each cancer by municipalities (**a**) Age-standardized incidence rates of breast cancer (**b**) Age-standardized incidence rates of prostate cancer (**c**) Age-standardized incidence rates of cervical cancer. ASR: Age-Standardized Rates. Data source: new cases of cancer reported to the National Administrative Cancer Registry during 2018

Figure 1 shows the municipalities with the highest incidence rates. We identified a heterogeneous pattern depending on the type of cancer. Nearly one-half of new cases occurred in five capital cities (Bogotá D.C., Medellín, Cali, Cartagena, and Barranquilla). Agua de Dios, a municipality in Cundinamarca had the highest incidence rate for breast cancer (1502.12; CI 95%: 655.84–2716.23) and were in the top 3 for prostate (218.42; CI 95%: 71.43–470.35) and cervical (364.16; CI 95%: 167.78–657.83) cancer. The highest incidence rates for prostate (364.02; CI 95%: 9.22–1407.09) and cervical (665.88; CI 95%: 16.86–2545.84) cancer were observed in Cucutilla (Norte de Santander) and Labranzagrande (Boyacá).

**Fig. 2:**
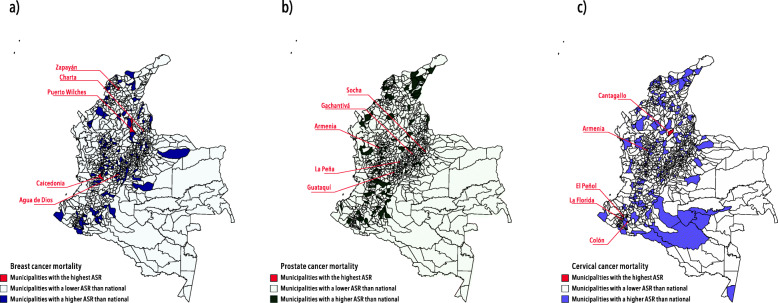
Age-standardized mortality rates of breast, prostate and cervical cancer by municipalities, Colombia 2018. Figure 2 shows the age-standardized mortality rates for each cancer by municipalities (**a**) Age-standardized mortality rates of breast cancer (**b**) Age-standardized mortality rates of prostate cancer (**c**) Age-standardized mortality rates of cervical cancer. ASR: Age-Standardized Rates. Data source: cancer deaths reported to the National Administrative Cancer Registry during 2018

On the other hand, the highest mortality rates across the municipalities are presented in Fig. 2. Close to 50.00% of the cancer deaths occurred in the same capital cities in the top for incidence (Bogotá D.C., Cali, Medellín, Barranquilla, and Cartagena). Agua de Dios, Socha (Boyacá) and El Peñol (Nariño) had the highest ASR for breast (191.63; CI 95%: 48.94–458.07), prostate (782.39; CI 95%: 19.81–2987.81) and cervical cancer (2086.38; CI 95%: 253.70–5918.24), respectively. See Tables S1 and S2 of Supplementary Material for complete information about incidence and mortality rates by municipalities.

## Discussion

During 2018, breast cancer was the leading cause of incidence and mortality in Colombia. Most new cancer cases (~ 50.00%) occurred in Central and Bogotá D.C. regions as well as in the contributory insurance, conversely, cervical cancer was more frequent in the subsidized. Regards incidence by region, there was a homogeneous pattern for breast and prostate cancer. Some regions showed significantly higher incidence rates than national, varying by type of cancer, as follows: Bogotá D.C. and Central for breast cancer, Bogotá D.C. for prostate cancer and “other departments” for cervical cancer. Consistently, Eastern region had both, incidence and mortality rates, significantly lower than national for all types of cancer. By municipalities, Agua de Dios in Cundinamarca was among the highest incidence rates for breast and cervical cancer and they were significantly higher than national. Mortality patterns by municipality were highly heterogeneous.

There are differences in ASR for all types of cancer between CAC and GLOBOCAN estimations. Generally, GLOBOCAN rates overestimate both, incidence and mortality rates reported by CAC. In 2018, the biggest difference was observed for prostate cancer (CAC: 11.34 vs. GLOBOCAN: 49.80), followed by breast (CAC: 18.69 vs. GLOBOCAN: 44.10) and cervical cancer (CAC: 5.93 vs. GLOBOCAN: 12.70). The same pattern was found for mortality in prostate (CAC: 7.58 vs. GLOBOCAN: 12.00), breast (CAC: 10.48 vs. GLOBOCAN: 11.90) and cervical cancer (CAC: 4.31 vs. GLOBOCAN: 5.70). Differences are consistent with a previous study conducted by the CAC and could be explained by the data sources and methodology used. In the case of GLOBOCAN, data on incidence come from four Colombian city-based registries that have been classified as high quality and the calculation is based on projections, whereas CAC information provided by health insurers is updated yearly [12].

In both sexes combined, the leading causes of new cases of cancer and deaths in Colombia are different from global trends. While in the world, lung cancer was the most commonly diagnosed and the leading cause of death [2], in Colombia was breast cancer. Compared with LAC, breast cancer was also the leading type among new cases and unlike Colombia, mortality trend was similar to the world [2].

Differences in cancer distribution between Colombia and the world reflect the ongoing social, economic, and health care changes in LAC. Such remarkable geographical contrast can be explained by differences in exposure to risk factors (reproductive, dietary, hormonal and environmental) and serious inequalities in timely access to screening and effective cancer treatment [5, 13].

Discrepancies in the distribution of incidence and mortality worldwide were also reflected in Colombia’s regions. In terms of incidence, there was a homogeneous trend in breast and prostate cancer, with the highest ASR in Bogotá D.C., Central and Pacific regions, being significantly higher than the national in Bogotá D.C. This pattern could be explained by geographical proximity and similar social and economic development, which from a broad epidemiological perspective, implies a comparative distribution of risk background and access to quality care. Furthermore, domestic differences on prostate cancer incidence could also be explained by the distribution of ethnic or genetic variations across the country that have been linked with a higher risk of this type of cancer [14–16]. Indeed, regions with the highest incidence coincide with a high proportion of Afro-Colombian population [17]. Otherwise, prostate cancer screening coverage has also been associated with incidence rates and more than other cancer, screening with the prostate-specific antigen (PSA) increases the probability of being diagnosed [18]. In fact, countries with a high PSA screening coverage also have higher incidence rates, early diagnosis and lower mortality rates [19]. In Colombia, an organized population screening is not recommended. Early detection is focused on men aged > 50 years or those aged < 50 years with known risk factors and screening interval should not be inferior to 5 years [20, 21]. According to the national health survey, conducted in 2015, coverage of PSA screening in men older than 50 years was 44.60% and it varied by insurance, geographic location, education and socioeconomic level, being higher in Bogotá D. C while the lowest was identified in “other departments” [22]. This coverage corresponds to the magnitude of prostate cancer incidence in those regions.

On the other hand, the incidence pattern for cervical cancer was different, showing a significantly higher ASR in “other departments” region, mainly composed of nonmetropolitan and rural areas. It has been reported that women in rural areas may experience barriers to optimal cervical cancer prevention, screening, and treatment, as well as, a higher frequency of risky sexual behaviors [23, 24].

Regarding mortality, its distribution varied widely between regions, being the Eastern region the one that had lower ASR than national for all types of cancer. According to previous studies, disparities in mortality can be explained by diagnosis in advanced stages, limited access to quality health services, and treatment opportunity [25–27]. However, in our study population, there were no differences between the proportion of people diagnosed with invasive neoplasms or the first treatment initiation.

As the trend is evaluated at a lower level, such as municipalities, there is more variability in patterns for both, incidence and mortality. Nevertheless, Agua de Dios in Cundinamarca was among the significantly higher incidence rates than the national for all types of cancer. The above may suggest a high prevalence of known risk factors in the municipality, as well as, limited access to screening, early diagnosis, and quality treatment.

Although there is a national policy for cancer attention [28], differences in incidence and mortality trends can be related to local approaches for implementing health programs and emerging social and economic changes typical of each region, department or municipality.

Finally, some studies have documented similar results and remarkable findings. According to the analysis of the cancer situation in 2015 performed by the Colombian Cancer Institute, in the city of Pasto, its population-based cancer registry showed how types of cancer, such as breast and prostate cancer had a higher incidence in urban areas while cervical cancer was commonly diagnosed in rural areas [22]. Likewise, a report prepared by the National Health Observatory showed that there was a high health inequality across the country for these type of cancer. For example, in breast cancer, a higher concentration of cases and deaths was observed in regions with the highest wealth per capita, in women belonging to the contributory insurance and with increased access to mammography and specialized centers [29, 30].

Regarding cervical and prostate cancer, their findings suggest that higher mortality rates were reported in regions with noteworthy socioeconomic inequality. They found that the lowest cervical cancer mortality rates were observed in the richest municipalities and at departmental/regional settings, they found a correlation between a higher income inequality (measured by the GINI index) and higher death rates. In the case of prostate cancer, the lowest rates were also reported in the richest municipalities, however, there was no evidence of a gradient consistent with municipal poverty levels. Surprisingly, at the departmental/regional level findings were contradictory, with a directly proportional correlation between income per capita and prostate cancer mortality rates, but and an inversely proportional association with the GINI inequality index [30].

### Strengths and limitations

This analysis has important strengths, including the large completeness of the NACR, which guarantees the external validity and utility of our findings in the evidence-informed health policymaking process at the national and regional levels. Furthermore, the accuracy and quality of the information of all new cases were verified by a data monitoring process.

On the other hand, some limitations should be discussed. First, the passive case reporting by the health insurers could lead to under-reporting. In any case, it would be a small proportion because the reporting process is mandatory [12]. The cross-sectional nature of the analysis does not allow establishing consistency in the trends we observed. Moreover, information bias cannot be ruled out because clinical records are the primary data source and they may be subject to error.

## Conclusions

We observed clear differences in cancer incidence and mortality across regions and municipalities, depending on each type of cancer. Our findings are important to improve the policy response from governments and health insurers regarding screening coverage, early detection, and access to treatment. Prioritization of health resources to geographical areas with incidence and mortality rates higher than national is crucial in a developing country, especially in breast, prostate, and cervical cancer, for which a better diagnosis and treatment can make a major difference in terms of survival, quality of life and economic burden of disease.

### Supplementary Information

**Additional file 1:** **Figure S1**. Socioeconomic distribution of regions in Colombia, 2018^1^. **Table S1.** Incidence rates for breast, prostate and cervical cancer by municipalities, Colombia 2018^1^. **Table S2**. Mortality rates for breast. Prostate and cervical cancer by municipalities. Colombia 2018^1^.

## Data Availability

The datasets generated and/or analyzed during the current study are not publicly available due they are owned and managed by the Colombian health system but are available from the corresponding author on reasonable request.

## Abbreviations

GLOBOCAN: Global Cancer Observatory
LAC: Latin American and the Caribbean
DALYs: Disability-Adjusted Life-Years
YLLs: Disability-Adjusted Life-Years
YLDs: Years Lived with Disability
NACR: National Administrative Cancer Registry
CAC: Cuenta de Alto Costo (High Cost Diseases Fund)
DANE: Departamento Administrativo Nacional de Estadística (Department for National Statistics)
IQR: Interquartile range
ASR: Age-standardized rate
PSA: Prostate-specific antigen
