# Supplementary material for: Patterns of breast, prostate and cervical cancer incidence and mortality in Colombia: an administrative registry data analysis
Source: BMC Cancer. 2020 Nov 11;20:1097. doi: 10.1186/s12885-020-07611-9 (PMC7661250; doi:10.1186/s12885-020-07611-9)
Supplement: Supplementary file 1 — Additional file 1: Figure S1. Socioeconomic distribution of regions in Colombia, 20181. Table S1. Incidence rates for breast, prostate and cervical cancer by municipalities, Colombia 20181. Table S2. Mortality rates for breast. Prostate and cervical cancer by municipalities. Colombia 20181. [file 12885_2020_7611_MOESM1_ESM.zip › Supplementary materialR3.docx]

**Figure S1. Socioeconomic distribution of regions in Colombia, 2018^1^**


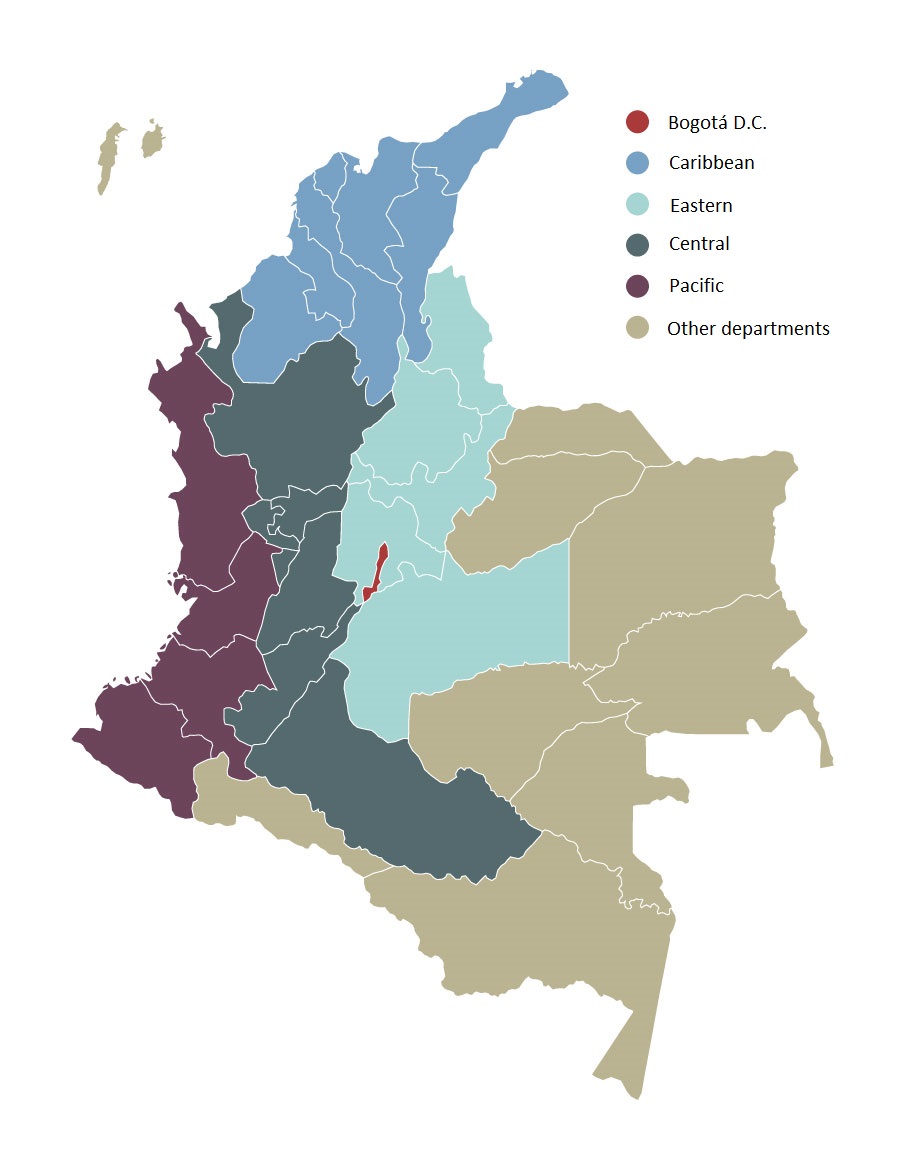


^1^ Colombian regions were defined by the Department for National Statistics (DANE, by its acronym in Spanish), grouping the Colombia’s 32 departments according to the gross domestic product in 6 regions: Bogotá D.C, Central, Eastern, Pacific, Caribbean and Other departments.

**Table S1. Incidence rates for breast, prostate and cervical cancer by municipalities, Colombia 2018^1^**

| **Municipality** | **Breast cancer** | | **Cervical cancer** | | **Prostate cancer** | |
| --- | --- | --- | --- | --- | --- | --- |
|  | **n** | **ASR (95% CI)** | **n** | **ASR (95% CI)** | **n** | **ASR (95% CI)** |
| Abejorral | 2 | 7.26 (0.64 - 54.09) | 0 | 0.00 (0.00 - 0.00) | 1 | 1.38 (0.03 - 76.4) |
| Abrego | 1 | 0.77 (0.02 - 27.83) | 0 | 0.00 (0.00 - 0.00) | 0 | 0.00 (0.00 - 0.00) |
| Acacías | 3 | 4.55 (0.76 - 18.75) | 1 | 4.23 (0.11 - 22.1) | 2 | 5.08 (0.46 - 21.41) |
| Acevedo | 3 | 0.58 (0.08 - 33.69) | 0 | 0.00 (0.00 - 0.00) | 1 | 1.34 (0.03 - 28.72) |
| Agua de Dios | 23 | 1,502.12 (655.84 - 2,716.23) | 12 | 364.16 (167.78 - 657.83) | 10 | 218.42 (71.43 - 470.35) |
| Aguachica | 7 | 12.66 (4.4 - 30.37) | 1 | 0.28 (0.01 - 91.05) | 5 | 2.92 (0.77 - 13.04) |
| Aguadas | 0 | 0.00 (0.00 - 0.00) | 1 | 1.11 (0.03 - 53.9) | 1 | 1.66 (0.04 - 48.58) |
| Aguazul | 2 | 9.85 (0.67 - 68.24) | 2 | 5.81 (0.66 - 31.75) | 4 | 6.84 (1.86 - 28.94) |
| Agustín Codazzi | 1 | 0.44 (0.01 - 20.25) | 1 | 31.37 (0.79 - 122.45) | 0 | 0.00 (0.00 - 0.00) |
| Aipe | 0 | 0.00 (0.00 - 0.00) | 1 | 6.28 (0.16 - 53.63) | 1 | 2.89 (0.07 - 89.43) |
| Albán (Cundinamarca) | 0 | 0.00 (0.00 - 0.00) | 0 | 0.00 (0.00 - 0.00) | 1 | 10.3 (0.26 - 165.43) |
| Albán (Nariño) | 0 | 0.00 (0.00 - 0.00) | 1 | 2.39 (0.06 - 57.81) | 3 | 18.03 (2.35 - 68.05) |
| Albania (Caquetá) | 1 | 15.43 (0.39 - 200.91) | 0 | 0.00 (0.00 - 0.00) | 0 | 0.00 (0.00 - 0.00) |
| Albania (La Guajira) | 1 | 2.18 (0.06 - 41.65) | 0 | 0.00 (0.00 - 0.00) | 1 | 4.99 (0.13 - 51.95) |
| Alcalá | 0 | 0.00 (0.00 - 0.00) | 2 | 1.35 (0.15 - 84.27) | 0 | 0.00 (0.00 - 0.00) |
| Aldana | 0 | 0.00 (0.00 - 0.00) | 1 | 30.19 (0.76 - 188.27) | 1 | 4.69 (0.12 - 222.47) |
| Alejandría | 1 | 719.84 (18.22 – 2,762.24) | 0 | 0.00 (0.00 - 0.00) | 0 | 0.00 (0.00 - 0.00) |
| Algarrobo | 1 | 4.48 (0.11 - 100.73) | 0 | 0.00 (0.00 - 0.00) | 0 | 0.00 (0.00 - 0.00) |
| Algeciras | 1 | 26.33 (0.67 - 109.92) | 3 | 10.35 (1.11 - 69.65) | 2 | 10.06 (1.09 - 51.34) |
| Altamira | 0 | 0.00 (0.00 - 0.00) | 0 | 0.00 (0.00 - 0.00) | 1 | 70.71 (1.79 - 378.46) |
| Alto Baudo | 0 | 0.00 (0.00 - 0.00) | 0 | 0.00 (0.00 - 0.00) | 1 | 17.78 (0.45 - 114.07) |
| Amagá | 2 | 15.03 (1.77 - 62.8) | 0 | 0.00 (0.00 - 0.00) | 1 | 1.44 (0.04 - 29.55) |
| Amalfi | 2 | 10.72 (1.3 - 64.95) | 0 | 0.00 (0.00 - 0.00) | 0 | 0.00 (0.00 - 0.00) |
| Ambalema | 1 | 12.77 (0.32 - 173.47) | 0 | 0.00 (0.00 - 0.00) | 0 | 0.00 (0.00 - 0.00) |
| Ancuyá | 3 | 150.29 (19.36 - 559.09) | 0 | 0.00 (0.00 - 0.00) | 1 | 9.59 (0.24 - 129.58) |
| Andalucía | 0 | 0.00 (0.00 - 0.00) | 1 | 0.02 (0.00 - 110.07) | 1 | 4.94 (0.12 - 71.33) |
| Andes | 4 | 6.01 (1.24 - 28.97) | 1 | 2.18 (0.06 - 26.08) | 1 | 2.26 (0.06 - 21.12) |
| Angostura | 0 | 0.00 (0.00 - 0.00) | 0 | 0.00 (0.00 - 0.00) | 2 | 29.99 (2.13 - 142.74) |
| Anserma | 2 | 14.89 (0.68 - 59.69) | 0 | 0.00 (0.00 - 0.00) | 1 | 0.69 (0.02 - 30.36) |
| Ansermanuevo | 1 | 66.17 (1.68 - 258.81) | 0 | 0.00 (0.00 - 0.00) | 0 | 0.00 (0.00 - 0.00) |
| Anza | 0 | 0.00 (0.00 - 0.00) | 1 | 36.26 (0.92 - 211.62) | 0 | 0.00 (0.00 - 0.00) |
| Anzoátegui | 1 | 42.45 (1.07 - 173.8) | 0 | 0.00 (0.00 - 0.00) | 0 | 0.00 (0.00 - 0.00) |
| Apartadó | 10 | 7.39 (3.10 - 15.32) | 3 | 2.51 (0.32 - 9.41) | 2 | 4.71 (0.28 - 17.16) |
| Aquitania | 0 | 0.00 (0.00 - 0.00) | 0 | 0.00 (0.00 - 0.00) | 1 | 106.94 (2.71 - 418.10) |
| Arauca (Arauca) | 3 | 6.22 (0.94 - 28.21) | 5 | 14.37 (3.58 - 39.04) | 0 | 0.00 (0.00 - 0.00) |
| Arauquita | 0 | 0.00 (0.00 - 0.00) | 1 | 9.82 (0.25 - 45.99) | 0 | 0.00 (0.00 - 0.00) |
| Arboleda | 1 | 41.61 (1.05 - 208.07) | 1 | 22.04 (0.56 - 170.2) | 1 | 23.43 (0.59 - 194.54) |
| Arboledas | 0 | 0.00 (0.00 - 0.00) | 0 | 0.00 (0.00 - 0.00) | 1 | 1.99 (0.05 - 127.25) |
| Arboletes | 0 | 0.00 (0.00 - 0.00) | 2 | 21.42 (2.47 - 71.84) | 0 | 0.00 (0.00 - 0.00) |
| Arenal | 0 | 0.00 (0.00 - 0.00) | 1 | 2.65 (0.07 - 59.08) | 0 | 0.00 (0.00 - 0.00) |
| Argelia (Antioquia) | 1 | 1.03 (0.03 - 137.88) | 0 | 0.00 (0.00 - 0.00) | 0 | 0.00 (0.00 - 0.00) |
| Argelia (Valle del Cauca) | 0 | 0.00 (0.00 - 0.00) | 0 | 0.00 (0.00 - 0.00) | 1 | 4.65 (0.12 - 188.88) |
| Ariguaní | 1 | 7.98 (0.2 - 57.88) | 0 | 0.00 (0.00 - 0.00) | 0 | 0.00 (0.00 - 0.00) |
| Arjona | 6 | 5.56 (1.65 - 28.84) | 1 | 0.45 (0.01 - 10.82) | 8 | 20.94 (8.44 - 42.43) |
| Armenia (Antioquia) | 9 | 123.89 (50.49 - 475.95) | 1 | 191.69 (4.85 - 791.63) | 0 | 0.00 (0.00 - 0.00) |
| Armenia (Quindío) | 28 | 55.73 (20.88 - 108.34) | 7 | 8.42 (1.26 - 23.14) | 25 | 19.58 (11.32 - 30.91) |
| Armero | 2 | 28.48 (3.13 - 115.01) | 0 | 0.00 (0.00 - 0.00) | 0 | 0.00 (0.00 - 0.00) |
| Astrea | 0 | 0.00 (0.00 - 0.00) | 1 | 8.00 (0.2 - 64.96) | 1 | 8.42 (0.21 - 64.64) |
| Ataco | 1 | 3.04 (0.08 - 83.41) | 0 | 0.00 (0.00 - 0.00) | 1 | 0.5 (0.01 - 74.22) |
| Atrato | 0 | 0.00 (0.00 - 0.00) | 0 | 0.00 (0.00 - 0.00) | 1 | 30.78 (0.78 - 166.57) |
| Ayapel | 3 | 5.59 (1.1 - 27.32) | 0 | 0.00 (0.00 - 0.00) | 1 | 19.27 (0.49 - 75.68) |
| Bahía Solano | 0 | 0.00 (0.00 - 0.00) | 1 | 10.48 (0.27 - 168.94) | 0 | 0.00 (0.00 - 0.00) |
| Bajo Baudó | 1 | 12.09 (0.31 - 106.37) | 1 | 1.82 (0.05 - 102.56) | 1 | 15.31 (0.39 - 95.34) |
| Balboa (Cauca) | 0 | 0.00 (0.00 - 0.00) | 1 | 3.36 (0.09 - 38.40) | 0 | 0.00 (0.00 - 0.00) |
| Baranoa | 3 | 4.38 (0.73 - 32.48) | 2 | 1.87 (0.23 - 17.98) | 0 | 0.00 (0.00 - 0.00) |
| Barayá | 1 | 12.01 (0.3 - 110.81) | 0 | 0.00 (0.00 - 0.00) | 1 | 8.03 (0.20 - 82.92) |
| Barbacoas | 1 | 3.29 (0.08 - 27.21) | 1 | 1.36 (0.03 - 105.96) | 1 | 10.95 (0.28 - 50.89) |
| Barbosa (Antioquia) | 13 | 87.29 (34.68 - 169.01) | 0 | 0.00 (0.00 - 0.00) | 2 | 161.52 (4.31 - 612.34) |
| Barbosa (Santander) | 2 | 5.21 (0.63 - 38.9) | 1 | 0.98 (0.02 - 43.5) | 1 | 3.32 (0.08 - 46.15) |
| Barichara | 1 | 4.62 (0.12 - 205.28) | 0 | 0.00 (0.00 - 0.00) | 0 | 0.00 (0.00 - 0.00) |
| Barrancabermeja | 20 | 25.05 (14.02 - 40.55) | 5 | 1.41 (0.41 - 9.09) | 11 | 1.81 (0.50 - 10.41) |
| Barrancas | 1 | 2.6 (0.07 - 101.05) | 1 | 8.38 (0.21 - 111.16) | 1 | 2.5 (0.06 - 61.11) |
| Barranco de Loba | 3 | 22.99 (1.59 - 94.35) | 1 | 8.04 (0.20 - 94.96) | 0 | 0.00 (0.00 - 0.00) |
| Barranquilla | 138 | 21.08 (16.20 - 26.78) | 42 | 5.74 (4.00 - 7.97) | 91 | 24.24 (18.63 - 30.8) |
| Belén de Los Andaquies | 1 | 3.10 (0.08 - 766.19) | 0 | 0.00 (0.00 - 0.00) | 1 | 12.17 (0.31 - 98.88) |
| Belén de Umbría | 1 | 5.71 (0.14 - 45.18) | 0 | 0.00 (0.00 - 0.00) | 0 | 0.00 (0.00 - 0.00) |
| Belén (Nariño) | 1 | 0.53 (0.01 - 253.69) | 0 | 0.00 (0.00 - 0.00) | 0 | 0.00 (0.00 - 0.00) |
| Bello | 73 | 37.67 (26.9 - 50.76) | 14 | 11.08 (3.02 - 24.97) | 40 | 28.09 (18.93 - 39.58) |
| Belmira | 0 | 0.00 (0.00 - 0.00) | 0 | 0.00 (0.00 - 0.00) | 1 | 36.18 (0.92 - 190.68) |
| Betania | 1 | 7.64 (0.19 - 151.97) | 0 | 0.00 (0.00 - 0.00) | 0 | 0.00 (0.00 - 0.00) |
| Betulia (Antioquia) | 0 | 0.00 (0.00 - 0.00) | 0 | 0.00 (0.00 - 0.00) | 1 | 1.62 (0.04 - 153.3) |
| Boavita | 0 | 0.00 (0.00 - 0.00) | 1 | 19.88 (0.50 - 184.17) | 0 | 0.00 (0.00 - 0.00) |
| Bochalema | 0 | 0.00 (0.00 - 0.00) | 0 | 0.00 (0.00 - 0.00) | 1 | 29.71 (0.75 - 171.17) |
| Bogotá D.C. | 1012 | 18.16 (16.79 - 19.61) | 267 | 4.85 (3.92 - 5.91) | 648 | 44.19 (40.06 - 48.55) |
| Bojayá | 0 | 0.00 (0.00 - 0.00) | 1 | 20.45 (0.52 - 268.59) | 0 | 0.00 (0.00 - 0.00) |
| Bolívar (Cauca) | 2 | 8.71 (0.54 - 36.9) | 1 | 2.79 (0.07 - 28.16) | 0 | 0.00 (0.00 - 0.00) |
| Bolívar (Valle del Cauca) | 1 | 27.83 (0.70 - 133.25) | 0 | 0.00 (0.00 - 0.00) | 0 | 0.00 (0.00 - 0.00) |
| Bosconia | 1 | 3.59 (0.09 - 26.5) | 0 | 0.00 (0.00 - 0.00) | 2 | 20.19 (2.45 - 74.99) |
| Briceño (Antioquia) | 0 | 0.00 (0.00 - 0.00) | 1 | 2.76 (0.07 - 134.28) | 0 | 0.00 (0.00 - 0.00) |
| Bucaramanga | 111 | 31.62 (24.95 - 39.46) | 11 | 3.78 (1.49 - 7.63) | 55 | 9.37 (6.65 - 13.11) |
| Buenaventura | 13 | 37.15 (12.73 - 75.12) | 13 | 11.34 (2.96 - 25.92) | 5 | 28.27 (3.49 - 79.88) |
| Buenavista (Boyacá) | 1 | 56.66 (1.43 - 285.54) | 0 | 0.00 (0.00 - 0.00) | 0 | 0.00 (0.00 - 0.00) |
| Buenavista (Córdoba) | 2 | 10.47 (0.9 - 58.75) | 0 | 0.00 (0.00 - 0.00) | 0 | 0.00 (0.00 - 0.00) |
| Buenavista (Sucre) | 1 | 14.64 (0.37 - 106.8) | 0 | 0.00 (0.00 - 0.00) | 0 | 0.00 (0.00 - 0.00) |
| Buenos Aires | 0 | 0.00 (0.00 - 0.00) | 0 | 0.00 (0.00 - 0.00) | 2 | 9.31 (1.05 - 59.19) |
| Buesaco | 1 | 5.44 (0.14 - 42.95) | 3 | 36.43 (5.32 - 110.5) | 0 | 0.00 (0.00 - 0.00) |
| Bugalagrande | 1 | 10.25 (0.26 - 63.88) | 1 | 7.05 (0.18 - 59.61) | 2 | 3.21 (0.27 - 58.75) |
| Cáceres | 0 | 0.00 (0.00 - 0.00) | 1 | 0.39 (0.01 - 36.17) | 0 | 0.00 (0.00 - 0.00) |
| Caicedo | 1 | 13.05 (0.33 - 155.33) | 0 | 0.00 (0.00 - 0.00) | 0 | 0.00 (0.00 - 0.00) |
| Caicedonia | 3 | 11.61 (2.39 - 59.05) | 0 | 0.00 (0.00 - 0.00) | 0 | 0.00 (0.00 - 0.00) |
| Caimito | 1 | 6.12 (0.15 - 196.76) | 1 | 13.5 (0.34 - 110.23) | 0 | 0.00 (0.00 - 0.00) |
| Cajamarca | 1 | 2.04 (0.05 - 48.25) | 0 | 0.00 (0.00 - 0.00) | 1 | 4.26 (0.11 - 99.24) |
| Cajibío | 1 | 0.23 (0.01 - 31.3) | 1 | 0.74 (0.02 - 34.8) | 1 | 1.05 (0.03 - 23.67) |
| Cajicá | 1 | 0.41 (0.01 - 14.69) | 1 | 20.67 (0.52 - 80.46) | 3 | 20.11 (1.69 - 65.88) |
| Calamar (Bolívar) | 2 | 12.18 (0.49 - 60.13) | 3 | 24.79 (4.39 - 83.83) | 1 | 0.18 (0.00 - 43.13) |
| Calarca | 1 | 1.75 (0.04 - 16.87) | 0 | 0.00 (0.00 - 0.00) | 2 | 0.96 (0.12 - 10.88) |
| Caldas (Antioquia) | 14 | 34.18 (13.89 - 66.83) | 7 | 5.87 (2.04 - 17.9) | 4 | 26.61 (1.28 - 92.9) |
| Caldas (Boyacá) | 0 | 0.00 (0.00 - 0.00) | 0 | 0.00 (0.00 - 0.00) | 1 | 40.69 (1.03 - 322.79) |
| Cali | 394 | 29.06 (21.88 - 37.32) | 96 | 2.95 (2.20 - 3.94) | 268 | 15.78 (13.52 - 18.3) |
| Calima | 1 | 168.39 (4.26 - 643.35) | 0 | 0.00 (0.00 - 0.00) | 0 | 0.00 (0.00 - 0.00) |
| Caloto | 3 | 43.79 (7.55 - 127.45) | 1 | 6.06 (0.15 - 64.38) | 0 | 0.00 (0.00 - 0.00) |
| Campo de La Cruz | 2 | 82.08 (3.40 - 300.07) | 1 | 13.88 (0.35 - 120.89) | 1 | 1.65 (0.04 - 73.6) |
| Campoalegre | 2 | 11.82 (1.43 - 43.16) | 2 | 9.83 (1.12 - 38.43) | 2 | 5.77 (0.65 - 72.15) |
| Canalete | 1 | 0.69 (0.02 - 67.71) | 2 | 1.39 (0.08 - 110.14) | 0 | 0.00 (0.00 - 0.00) |
| Candelaria (Atlántico) | 5 | 162.14 (40.17 - 406.07) | 2 | 12.3 (1.49 - 140.73) | 0 | 0.00 (0.00 - 0.00) |
| Candelaria (Valle del Cauca) | 9 | 18.03 (3.71 - 47.21) | 0 | 0.00 (0.00 - 0.00) | 2 | 9.16 (1.11 - 30.54) |
| Cantagallo | 0 | 0.00 (0.00 - 0.00) | 1 | 1.18 (0.03 - 251.16) | 1 | 8.69 (0.22 - 125.2) |
| Cañasgordas | 1 | 6.42 (0.16 - 66.71) | 1 | 0.93 (0.02 - 121.08) | 1 | 6.48 (0.16 - 54.86) |
| Caramanta | 0 | 0.00 (0.00 - 0.00) | 1 | 12.08 (0.31 - 225.48) | 0 | 0.00 (0.00 - 0.00) |
| Carepa | 1 | 4.55 (0.12 - 32.45) | 2 | 4.21 (0.48 - 23.94) | 0 | 0.00 (0.00 - 0.00) |
| Carolina | 1 | 31.83 (0.81 - 340.49) | 1 | 8.33 (0.21 - 276.12) | 0 | 0.00 (0.00 - 0.00) |
| Cartagena | 140 | 31.72 (24.62 - 39.9) | 43 | 10.91 (4.90 - 19.65) | 69 | 6.67 (4.97 - 8.84) |
| Cartagena del Chairá | 1 | 0.32 (0.01 - 30.38) | 0 | 0.00 (0.00 - 0.00) | 0 | 0.00 (0.00 - 0.00) |
| Cartago | 15 | 22.76 (8.74 - 45.26) | 6 | 3.7 (1.24 - 10.59) | 7 | 4.76 (1.83 - 12.56) |
| Casabianca | 0 | 0.00 (0.00 - 0.00) | 0 | 0.00 (0.00 - 0.00) | 1 | 11.41 (0.29 - 312.17) |
| Caucasia | 8 | 8.52 (3.24 - 19.3) | 7 | 10.87 (3.36 - 38.98) | 1 | 3.53 (0.09 - 16.24) |
| Cereté | 11 | 4.28 (1.59 - 18.31) | 4 | 1.11 (0.24 - 14.01) | 3 | 6.13 (1.21 - 20.84) |
| Cerro San Antonio | 0 | 0.00 (0.00 - 0.00) | 0 | 0.00 (0.00 - 0.00) | 1 | 2.45 (0.06 - 116.1) |
| Chachagüí | 2 | 0.38 (0.04 - 123.19) | 0 | 0.00 (0.00 - 0.00) | 1 | 28.99 (0.73 - 136.04) |
| Chalán | 1 | 6.47 (0.16 - 850.95) | 1 | 30.05 (0.76 - 285.44) | 0 | 0.00 (0.00 - 0.00) |
| Chaparral | 1 | 42.21 (1.07 - 162.25) | 0 | 0.00 (0.00 - 0.00) | 1 | 0.63 (0.02 - 16.45) |
| Charalá | 0 | 0.00 (0.00 - 0.00) | 0 | 0.00 (0.00 - 0.00) | 2 | 4.71 (0.30 - 154.63) |
| Charta | 0 | 0.00 (0.00 - 0.00) | 0 | 0.00 (0.00 - 0.00) | 1 | 84.21 (2.13 - 522.48) |
| Chía | 12 | 14.16 (5.77 - 30.69) | 2 | 0.25 (0.02 - 10.48) | 10 | 11.37 (3.16 - 27.82) |
| Chigorodó | 1 | 0.84 (0.02 - 51.4) | 0 | 0.00 (0.00 - 0.00) | 0 | 0.00 (0.00 - 0.00) |
| Chimichagua | 1 | 1.65 (0.04 - 47.85) | 0 | 0.00 (0.00 - 0.00) | 0 | 0.00 (0.00 - 0.00) |
| Chinácota | 3 | 248.76 (8.83 - 908.11) | 0 | 0.00 (0.00 - 0.00) | 1 | 23.43 (0.59 - 108.08) |
| Chinchiná | 2 | 0.94 (0.11 - 19.67) | 1 | 1.30 (0.03 - 19.95) | 2 | 6.01 (0.38 - 28.3) |
| Chinú | 3 | 41.16 (2.54 - 137.65) | 0 | 0.00 (0.00 - 0.00) | 2 | 68.48 (1.84 - 260.47) |
| Chipatá | 0 | 0.00 (0.00 - 0.00) | 1 | 13.56 (0.34 - 187.6) | 0 | 0.00 (0.00 - 0.00) |
| Chiquinquirá | 3 | 1.07 (0.16 - 20.9) | 2 | 5.16 (0.21 - 23.56) | 3 | 21.25 (1.61 - 69.81) |
| Chíquiza | 0 | 0.00 (0.00 - 0.00) | 0 | 0.00 (0.00 - 0.00) | 1 | 42.17 (1.07 - 321.18) |
| Chiriguaná | 2 | 8.85 (1.07 - 76.48) | 0 | 0.00 (0.00 - 0.00) | 1 | 1.87 (0.05 - 317.82) |
| Chitagá | 1 | 9.71 (0.25 - 94.7) | 0 | 0.00 (0.00 - 0.00) | 1 | 5.61 (0.14 - 547.6) |
| Chivolo | 0 | 0.00 (0.00 - 0.00) | 1 | 50.11 (1.27 - 225.4) | 1 | 10.69 (0.27 - 71.51) |
| Chivor | 1 | 25.23 (0.64 - 998.88) | 0 | 0.00 (0.00 - 0.00) | 0 | 0.00 (0.00 - 0.00) |
| Cicuco | 2 | 3.78 (0.43 - 134.16) | 0 | 0.00 (0.00 - 0.00) | 0 | 0.00 (0.00 - 0.00) |
| Ciénaga de Oro | 4 | 10.99 (1.30 - 38.08) | 2 | 7.56 (0.84 - 29.97) | 3 | 5.32 (1.09 - 19.29) |
| Ciénaga (Magdalena) | 13 | 14.01 (6.92 - 27.42) | 4 | 3.99 (0.92 - 13.74) | 0 | 0.00 (0.00 - 0.00) |
| Cimitarra | 1 | 0.62 (0.02 - 27.88) | 0 | 0.00 (0.00 - 0.00) | 0 | 0.00 (0.00 - 0.00) |
| Cisneros | 2 | 81.33 (5.83 - 282.26) | 0 | 0.00 (0.00 - 0.00) | 0 | 0.00 (0.00 - 0.00) |
| Ciudad Bolívar | 2 | 33.84 (1.42 - 129.45) | 1 | 17.47 (0.44 - 82.27) | 1 | 7.01 (0.18 - 68.7) |
| Cocorná | 1 | 3.00 (0.08 - 65.41) | 0 | 0.00 (0.00 - 0.00) | 0 | 0.00 (0.00 - 0.00) |
| Coello | 0 | 0.00 (0.00 - 0.00) | 0 | 0.00 (0.00 - 0.00) | 1 | 6.17 (0.16 - 100.55) |
| Colombia | 0 | 0.00 (0.00 - 0.00) | 0 | 0.00 (0.00 - 0.00) | 1 | 17.6 (0.45 - 100.79) |
| Colón (Nariño) | 1 | 27.73 (0.7 - 142.2) | 0 | 0.00 (0.00 - 0.00) | 1 | 11.13 (0.28 - 103.15) |
| Coloso | 0 | 0.00 (0.00 - 0.00) | 0 | 0.00 (0.00 - 0.00) | 1 | 25.50 (0.65 - 174.04) |
| Cómbita | 1 | 1.04 (0.03 - 151.37) | 1 | 13.97 (0.35 - 119.19) | 1 | 1.78 (0.04 - 114.98) |
| Concepción (Antioquia) | 1 | 70.92 (1.8 - 408.71) | 1 | 84.7 (2.14 - 465.88) | 0 | 0.00 (0.00 - 0.00) |
| Concordia (Magdalena) | 0 | 0.00 (0.00 - 0.00) | 2 | 4.75 (0.22 - 152.43) | 1 | 5.80 (0.15 - 103.2) |
| Condoto | 0 | 0.00 (0.00 - 0.00) | 0 | 0.00 (0.00 - 0.00) | 1 | 3.80 (0.10 - 95.35) |
| Consaca | 1 | 17.44 (0.44 - 149.98) | 1 | 3.42 (0.09 - 101.44) | 0 | 0.00 (0.00 - 0.00) |
| Convención | 1 | 6.17 (0.16 - 90.79) | 0 | 0.00 (0.00 - 0.00) | 0 | 0.00 (0.00 - 0.00) |
| Copacabana | 15 | 24.97 (11.98 - 46.94) | 4 | 31.31 (3.76 - 91.07) | 6 | 32.58 (6.83 - 82.88) |
| Córdoba (Nariño) | 0 | 0.00 (0.00 - 0.00) | 1 | 3.2 (0.08 - 74.46) | 1 | 17.02 (0.43 - 97.7) |
| Corinto | 2 | 10.2 (0.82 - 45.17) | 0 | 0.00 (0.00 - 0.00) | 0 | 0.00 (0.00 - 0.00) |
| Corozal | 5 | 4.75 (1.46 - 17.89) | 1 | 1.53 (0.04 - 21.34) | 1 | 2.64 (0.07 - 19.56) |
| Cota | 2 | 10.24 (1.13 - 68.17) | 1 | 16.49 (0.42 - 74.08) | 0 | 0.00 (0.00 - 0.00) |
| Cotorra | 0 | 0.00 (0.00 - 0.00) | 0 | 0.00 (0.00 - 0.00) | 2 | 17.11 (1.88 - 80.97) |
| Covarachía | 1 | 79.44 (2.01 - 692.56) | 0 | 0.00 (0.00 - 0.00) | 0 | 0.00 (0.00 - 0.00) |
| Coyaima | 2 | 10.12 (0.86 - 53.83) | 0 | 0.00 (0.00 - 0.00) | 0 | 0.00 (0.00 - 0.00) |
| Cubarral | 0 | 0.00 (0.00 - 0.00) | 1 | 9.28 (0.23 - 318.26) | 0 | 0.00 (0.00 - 0.00) |
| Cúcuta | 63 | 12.51 (8.32 - 17.91) | 29 | 2.35 (1.29 - 4.56) | 27 | 10.76 (6.71 - 16.31) |
| Cucutilla | 0 | 0.00 (0.00 - 0.00) | 1 | 2.27 (0.06 - 134.05) | 1 | 364.02 (9.22 - 1407.09) |
| Cuítiva | 4 | 233.96 (47.77 – 1,452.27) | 1 | 63.28 (1.6 - 810.12) | 1 | 6.56 (0.17 - 442.62) |
| Cumaribo | 0 | 0.00 (0.00 - 0.00) | 1 | 1.67 (0.04 - 83.89) | 0 | 0.00 (0.00 - 0.00) |
| Cumbal | 3 | 3.95 (0.73 - 41.12) | 1 | 2.65 (0.07 - 32.13) | 1 | 8.89 (0.23 - 42.21) |
| Cunday | 1 | 3.57 (0.09 - 113.65) | 0 | 0.00 (0.00 - 0.00) | 1 | 1.97 (0.05 - 121.69) |
| Curillo | 0 | 0.00 (0.00 - 0.00) | 1 | 16.39 (0.42 - 567.51) | 0 | 0.00 (0.00 - 0.00) |
| Curumaní | 1 | 7.09 (0.18 - 61.24) | 0 | 0.00 (0.00 - 0.00) | 1 | 8.03 (0.2 - 67.24) |
| Dabeiba | 1 | 3.84 (0.10 - 47.68) | 1 | 4.15 (0.11 - 44.57) | 0 | 0.00 (0.00 - 0.00) |
| Dibulla | 1 | 1.54 (0.04 - 23.72) | 0 | 0.00 (0.00 - 0.00) | 0 | 0.00 (0.00 - 0.00) |
| Distracción | 1 | 47.32 (1.20 - 192.12) | 0 | 0.00 (0.00 - 0.00) | 0 | 0.00 (0.00 - 0.00) |
| Dolores | 1 | 86.24 (2.18 - 432.48) | 0 | 0.00 (0.00 - 0.00) | 0 | 0.00 (0.00 - 0.00) |
| Don Matías | 2 | 106.19 (2.97 - 402.05) | 1 | 3.89 (0.10 - 36.9) | 1 | 7.17 (0.18 - 110.2) |
| Dosquebradas | 19 | 10.92 (5.09 - 21.49) | 6 | 1.63 (0.50 - 13.49) | 9 | 27.17 (9.26 - 56.29) |
| Duitama | 10 | 3.41 (1.35 - 11.26) | 2 | 7.06 (0.20 - 27.77) | 5 | 10.74 (3.23 - 26.04) |
| El Bagre | 1 | 0.72 (0.02 - 98.41) | 5 | 25.18 (6.73 - 71.31) | 2 | 13.17 (1.37 - 46.66) |
| El Banco | 5 | 25.97 (1.82 - 91.06) | 1 | 2.92 (0.07 - 31.37) | 1 | 1.62 (0.04 - 48.43) |
| El Cairo | 2 | 94.99 (3.61 - 371.63) | 0 | 0.00 (0.00 - 0.00) | 1 | 10.09 (0.26 - 95.67) |
| El Carmen | 0 | 0.00 (0.00 - 0.00) | 2 | 27.71 (1.50 - 117.32) | 0 | 0.00 (0.00 - 0.00) |
| El Carmen de Atrato | 1 | 41.05 (1.04 - 172.56) | 0 | 0.00 (0.00 - 0.00) | 0 | 0.00 (0.00 - 0.00) |
| El Carmen de Bolívar | 11 | 54.41 (15.11 - 123.3) | 4 | 16.67 (3.00 - 46.17) | 2 | 0.86 (0.08 - 13.56) |
| El Carmen de Chucurí | 1 | 28.72 (0.73 - 122.7) | 0 | 0.00 (0.00 - 0.00) | 0 | 0.00 (0.00 - 0.00) |
| El Carmen de Viboral | 4 | 2.65 (0.70 - 87.97) | 0 | 0.00 (0.00 - 0.00) | 3 | 21.23 (2.90 - 63.07) |
| El Cerrito | 6 | 18.04 (4.22 - 48.49) | 2 | 3.01 (0.11 - 20.19) | 4 | 17.58 (4.79 - 44.89) |
| El Colegio | 1 | 12.46 (0.32 - 80.95) | 0 | 0.00 (0.00 - 0.00) | 1 | 4.58 (0.12 - 46.5) |
| El Doncello | 1 | 5.73 (0.15 - 49.11) | 2 | 6.51 (0.79 - 69.19) | 1 | 1.05 (0.03 - 58.48) |
| El Guamo | 2 | 16.65 (1.28 - 206.33) | 0 | 0.00 (0.00 - 0.00) | 1 | 4.14 (0.10 - 128.27) |
| El Paso | 0 | 0.00 (0.00 - 0.00) | 2 | 3.66 (0.42 - 202.27) | 0 | 0.00 (0.00 - 0.00) |
| El Paujil | 1 | 7.63 (0.19 - 65.04) | 0 | 0.00 (0.00 - 0.00) | 1 | 8.25 (0.21 - 65.78) |
| El Peñón (Bolívar) | 1 | 12.12 (0.31 - 135.04) | 0 | 0.00 (0.00 - 0.00) | 0 | 0.00 (0.00 - 0.00) |
| El Retén | 0 | 0.00 (0.00 - 0.00) | 1 | 2.73 (0.07 - 61.01) | 0 | 0.00 (0.00 - 0.00) |
| El Rosal | 1 | 14.05 (0.36 - 137.55) | 0 | 0.00 (0.00 - 0.00) | 0 | 0.00 (0.00 - 0.00) |
| El Rosario | 0 | 0.00 (0.00 - 0.00) | 0 | 0.00 (0.00 - 0.00) | 1 | 38.02 (0.96 - 182.97) |
| El Santuario | 1 | 1.19 (0.03 - 46.62) | 0 | 0.00 (0.00 - 0.00) | 1 | 1.65 (0.04 - 33.5) |
| El Tablón de Gómez | 0 | 0.00 (0.00 - 0.00) | 0 | 0.00 (0.00 - 0.00) | 1 | 5.41 (0.14 - 162.16) |
| El Tambo (Cauca) | 0 | 0.00 (0.00 - 0.00) | 1 | 3.28 (0.08 - 23.89) | 3 | 1.2 (0.23 - 22.96) |
| El Tambo (Nariño) | 1 | 16.92 (0.43 - 119.85) | 0 | 0.00 (0.00 - 0.00) | 0 | 0.00 (0.00 - 0.00) |
| El Zulia | 1 | 0.75 (0.02 - 59.9) | 1 | 2.25 (0.06 - 53) | 1 | 13.00 (0.33 - 79.49) |
| Envigado | 69 | 34.71 (21.95 - 51.32) | 7 | 2.11 (0.78 - 7.28) | 34 | 18.7 (10.70 - 30.11) |
| Espinal | 11 | 11.87 (4.98 - 35.45) | 2 | 1.44 (0.14 - 39.16) | 6 | 16.45 (4.78 - 38.87) |
| Facatativá | 5 | 10.51 (0.61 - 36.77) | 1 | 0.92 (0.02 - 7.3) | 4 | 3.66 (0.99 - 10.9) |
| Falan | 0 | 0.00 (0.00 - 0.00) | 0 | 0.00 (0.00 - 0.00) | 1 | 9.99 (0.25 - 249.85) |
| Filandia | 1 | 9.70 (0.25 - 78.12) | 0 | 0.00 (0.00 - 0.00) | 1 | 1.43 (0.04 - 91.04) |
| Florencia (Caquetá) | 12 | 33.29 (9.15 - 74.32) | 11 | 11.56 (4.82 - 22.8) | 10 | 8.54 (3.92 - 16.89) |
| Florencia (Cauca) | 0 | 0.00 (0.00 - 0.00) | 0 | 0.00 (0.00 - 0.00) | 1 | 8.22 (0.21 - 178.62) |
| Florida | 2 | 2.52 (0.31 - 17.86) | 0 | 0.00 (0.00 - 0.00) | 1 | 1.56 (0.04 - 16.44) |
| Floridablanca | 29 | 19.90 (12.49 - 29.98) | 2 | 0.41 (0.01 - 8.02) | 13 | 12.94 (4.57 - 26.67) |
| Fomeque | 0 | 0.00 (0.00 - 0.00) | 0 | 0.00 (0.00 - 0.00) | 1 | 1.40 (0.04 - 68.20) |
| Fonseca | 2 | 3.66 (0.43 - 31.86) | 2 | 26.89 (0.84 - 106.75) | 0 | 0.00 (0.00 - 0.00) |
| Francisco Pizarro | 0 | 0.00 (0.00 - 0.00) | 1 | 8.01 (0.20 - 555.19) | 0 | 0.00 (0.00 - 0.00) |
| Fredonia | 0 | 0.00 (0.00 - 0.00) | 0 | 0.00 (0.00 - 0.00) | 2 | 9.37 (0.95 - 49.10) |
| Fresno | 1 | 1.45 (0.04 - 58.58) | 0 | 0.00 (0.00 - 0.00) | 1 | 1.26 (0.03 - 33.31) |
| Frontino | 1 | 1.38 (0.04 - 141.54) | 1 | 7.66 (0.19 - 82.95) | 0 | 0.00 (0.00 - 0.00) |
| Fundación | 3 | 3.58 (0.64 - 25.21) | 0 | 0.00 (0.00 - 0.00) | 1 | 1.29 (0.03 - 45.07) |
| Funza | 0 | 0.00 (0.00 - 0.00) | 0 | 0.00 (0.00 - 0.00) | 3 | 50.20 (1.66 - 185.13) |
| Fúquene | 0 | 0.00 (0.00 - 0.00) | 0 | 0.00 (0.00 - 0.00) | 1 | 30.62 (0.78 - 707.52) |
| Fusagasugá | 8 | 9.23 (3.01 - 21.17) | 3 | 3.07 (0.48 - 11.30) | 3 | 0.64 (0.12 - 17.70) |
| Gachancipá | 1 | 2.29 (0.06 - 62.81) | 0 | 0.00 (0.00 - 0.00) | 0 | 0.00 (0.00 - 0.00) |
| Galapa | 1 | 1.03 (0.03 - 32.18) | 0 | 0.00 (0.00 - 0.00) | 2 | 25.64 (2.95 - 82.99) |
| Galeras | 2 | 26.49 (2.22 - 94.59) | 0 | 0.00 (0.00 - 0.00) | 1 | 7.86 (0.20 - 74.36) |
| Gamarra | 1 | 9.80 (0.25 - 66.65) | 0 | 0.00 (0.00 - 0.00) | 0 | 0.00 (0.00 - 0.00) |
| Gameza | 0 | 0.00 (0.00 - 0.00) | 0 | 0.00 (0.00 - 0.00) | 1 | 12.16 (0.31 - 239.45) |
| Garzón | 5 | 7.71 (1.09 - 25.58) | 2 | 1.73 (0.13 - 13.69) | 2 | 3.15 (0.37 - 14.31) |
| Gigante | 4 | 13.04 (3.54 - 41.65) | 0 | 0.00 (0.00 - 0.00) | 0 | 0.00 (0.00 - 0.00) |
| Ginebra | 3 | 22.54 (1.88 - 88.79) | 0 | 0.00 (0.00 - 0.00) | 1 | 4.48 (0.11 - 66.72) |
| Girardot | 10 | 27.19 (5.59 - 67.74) | 3 | 14.75 (2.44 - 40.80) | 5 | 10.16 (1.57 - 29.55) |
| Girardota | 7 | 28.96 (4.45 - 80.35) | 0 | 0.00 (0.00 - 0.00) | 5 | 11.08 (3.41 - 29.28) |
| Girón | 7 | 5.46 (1.02 - 16.08) | 4 | 1.04 (0.28 - 7.95) | 3 | 3.17 (0.44 - 11.07) |
| Granada (Antioquia) | 0 | 0.00 (0.00 - 0.00) | 0 | 0.00 (0.00 - 0.00) | 1 | 4.35 (0.11 - 94.21) |
| Granada (Meta) | 3 | 108.13 (3.45 - 400.11) | 1 | 0.63 (0.02 - 43.91) | 3 | 37.65 (1.59 - 134.86) |
| Guacarí | 7 | 30.19 (7.53 - 79.82) | 2 | 1.72 (0.21 - 35.96) | 1 | 0.29 (0.01 - 45.32) |
| Guachené | 0 | 0.00 (0.00 - 0.00) | 0 | 0.00 (0.00 - 0.00) | 2 | 24.81 (0.70 - 109.39) |
| Guachucal | 3 | 37.00 (4.58 - 147.94) | 0 | 0.00 (0.00 - 0.00) | 0 | 0.00 (0.00 - 0.00) |
| Guadalajara de Buga | 17 | 38.41 (9.30 - 90.4) | 3 | 2.29 (0.33 - 12.41) | 6 | 2.68 (0.82 - 11.27) |
| Guadalupe (Huila) | 2 | 76.86 (5.97 - 253.46) | 0 | 0.00 (0.00 - 0.00) | 0 | 0.00 (0.00 - 0.00) |
| Guaduas | 0 | 0.00 (0.00 - 0.00) | 1 | 5.70 (0.14 - 33.73) | 0 | 0.00 (0.00 - 0.00) |
| Guaitarilla | 2 | 4.41 (0.36 - 138.26) | 0 | 0.00 (0.00 - 0.00) | 1 | 8.24 (0.21 - 80.27) |
| Guamal (Magdalena) | 1 | 1.09 (0.03 - 37.19) | 2 | 17.7 (2.14 - 66.09) | 1 | 15.29 (0.39 - 66.24) |
| Guamo | 1 | 3.35 (0.08 - 39.53) | 0 | 0.00 (0.00 - 0.00) | 0 | 0.00 (0.00 - 0.00) |
| Guapi | 0 | 0.00 (0.00 - 0.00) | 2 | 14.23 (1.22 - 71.95) | 1 | 10.83 (0.27 - 71.53) |
| Guarne | 5 | 99.80 (2.61 - 378.84) | 2 | 3.82 (0.35 – 23.00) | 7 | 2.59 (0.98 - 21.72) |
| Guatapé | 1 | 29.74 (0.75 - 221.87) | 0 | 0.00 (0.00 - 0.00) | 0 | 0.00 (0.00 - 0.00) |
| Guataquí | 0 | 0.00 (0.00 - 0.00) | 0 | 0.00 (0.00 - 0.00) | 1 | 165.86 (4.20 - 776.90) |
| Guateque | 0 | 0.00 (0.00 - 0.00) | 1 | 1.04 (0.03 - 105.67) | 0 | 0.00 (0.00 - 0.00) |
| Guática | 0 | 0.00 (0.00 - 0.00) | 1 | 4.04 (0.10 - 84.10) | 0 | 0.00 (0.00 - 0.00) |
| Guayabal de Siquima | 0 | 0.00 (0.00 - 0.00) | 0 | 0.00 (0.00 - 0.00) | 1 | 13.32 (0.34 - 316.65) |
| Güepsa | 0 | 0.00 (0.00 - 0.00) | 0 | 0.00 (0.00 - 0.00) | 1 | 21.97 (0.56 - 277.86) |
| Hacarí | 1 | 19.11 (0.48 - 128.99) | 0 | 0.00 (0.00 - 0.00) | 0 | 0.00 (0.00 - 0.00) |
| Hatillo de Loba | 0 | 0.00 (0.00 - 0.00) | 1 | 12.49 (0.32 - 104.8) | 1 | 8.43 (0.21 - 85.98) |
| Hato | 0 | 0.00 (0.00 - 0.00) | 1 | 4.47 (0.11 - 504.34) | 0 | 0.00 (0.00 - 0.00) |
| Hato Corozal | 1 | 7.65 (0.19 - 200.05) | 1 | 6.9 (0.17 - 394.96) | 0 | 0.00 (0.00 - 0.00) |
| Herrán | 1 | 33.73 (0.85 - 333.04) | 0 | 0.00 (0.00 - 0.00) | 0 | 0.00 (0.00 - 0.00) |
| Hobo | 1 | 0.72 (0.02 - 260.67) | 0 | 0.00 (0.00 - 0.00) | 1 | 49.11 (1.24 - 271.89) |
| Honda | 3 | 90.8 (7.68 - 287.43) | 0 | 0.00 (0.00 - 0.00) | 0 | 0.00 (0.00 - 0.00) |
| Ibagué | 62 | 20.54 (12.25 - 31.48) | 13 | 5.34 (2.29 - 10.17) | 23 | 3.05 (1.56 - 5.99) |
| Iles | 1 | 14.53 (0.37 - 169.25) | 0 | 0.00 (0.00 - 0.00) | 0 | 0.00 (0.00 - 0.00) |
| Imués | 1 | 11.12 (0.28 - 161.39) | 0 | 0.00 (0.00 - 0.00) | 0 | 0.00 (0.00 - 0.00) |
| Ipiales | 7 | 3.25 (0.83 - 11.39) | 5 | 3.96 (1.18 - 10.93) | 5 | 4.20 (1.33 - 11.49) |
| Isnos | 1 | 3.16 (0.08 - 44.02) | 0 | 0.00 (0.00 - 0.00) | 0 | 0.00 (0.00 - 0.00) |
| Istmina | 1 | 1.55 (0.04 - 76.37) | 0 | 0.00 (0.00 - 0.00) | 0 | 0.00 (0.00 - 0.00) |
| Itagui | 52 | 60.35 (39.8 - 87.49) | 12 | 8.13 (3.84 - 14.99) | 30 | 24.61 (11.15 - 44.54) |
| Jamundí | 4 | 1.91 (0.41 - 33.31) | 2 | 0.14 (0.01 - 10.7) | 7 | 9.11 (1.97 - 23.82) |
| Jardín | 1 | 5.34 (0.14 - 78.65) | 1 | 0.96 (0.02 - 78.18) | 1 | 12.08 (0.31 - 82.28) |
| Jesús María | 2 | 208.70 (15.25 - 842.93) | 0 | 0.00 (0.00 - 0.00) | 0 | 0.00 (0.00 - 0.00) |
| La Argentina | 1 | 20.10 (0.51 - 120.37) | 0 | 0.00 (0.00 - 0.00) | 0 | 0.00 (0.00 - 0.00) |
| La Belleza | 0 | 0.00 (0.00 - 0.00) | 1 | 4.28 (0.11 - 137.24) | 0 | 0.00 (0.00 - 0.00) |
| La Calera | 1 | 1.85 (0.05 - 46.07) | 0 | 0.00 (0.00 - 0.00) | 0 | 0.00 (0.00 - 0.00) |
| La Ceja | 6 | 20.41 (3.22 - 62.11) | 0 | 0.00 (0.00 - 0.00) | 6 | 23.69 (7.38 - 55.36) |
| La Celia | 1 | 8.89 (0.23 - 202.49) | 0 | 0.00 (0.00 - 0.00) | 0 | 0.00 (0.00 - 0.00) |
| La Cruz | 4 | 35.20 (7.09 - 114.59) | 1 | 12.59 (0.32 - 85.48) | 0 | 0.00 (0.00 - 0.00) |
| La Cumbre | 0 | 0.00 (0.00 - 0.00) | 1 | 7.02 (0.18 - 110.59) | 0 | 0.00 (0.00 - 0.00) |
| La Dorada | 3 | 2.95 (0.36 - 17.64) | 3 | 16.05 (3.16 - 43.51) | 0 | 0.00 (0.00 - 0.00) |
| La Estrella | 9 | 14.91 (4.29 - 38.33) | 1 | 0.52 (0.01 - 17.03) | 11 | 111.78 (23.72 - 273.04) |
| La Florida | 2 | 11.19 (1.12 - 198.56) | 0 | 0.00 (0.00 - 0.00) | 1 | 7.61 (0.19 - 97.78) |
| La Gloria | 1 | 2.81 (0.07 - 231.19) | 1 | 4.35 (0.11 - 227.89) | 0 | 0.00 (0.00 - 0.00) |
| La Llanada | 0 | 0.00 (0.00 - 0.00) | 0 | 0.00 (0.00 - 0.00) | 1 | 0.39 (0.01 - 171.8) |
| La Mesa | 1 | 7.00 (0.18 - 76.92) | 0 | 0.00 (0.00 - 0.00) | 3 | 62.92 (2.60 - 225.84) |
| La Montañita | 2 | 23.25 (1.20 - 113.54) | 0 | 0.00 (0.00 - 0.00) | 0 | 0.00 (0.00 - 0.00) |
| La Palma | 0 | 0.00 (0.00 - 0.00) | 0 | 0.00 (0.00 - 0.00) | 2 | 1.54 (0.19 - 244.6) |
| La Paz (Cesar) | 0 | 0.00 (0.00 - 0.00) | 1 | 0.14 (0.00 - 55.88) | 0 | 0.00 (0.00 - 0.00) |
| La Pintada | 1 | 20.51 (0.52 - 175.43) | 2 | 41.28 (2.43 - 397.74) | 0 | 0.00 (0.00 - 0.00) |
| La Plata | 2 | 0.74 (0.09 - 46.07) | 0 | 0.00 (0.00 - 0.00) | 0 | 0.00 (0.00 - 0.00) |
| La Sierra | 0 | 0.00 (0.00 - 0.00) | 1 | 5.61 (0.14 - 127.38) | 0 | 0.00 (0.00 - 0.00) |
| La Unión (Antioquia) | 1 | 6.22 (0.16 - 86.16) | 0 | 0.00 (0.00 - 0.00) | 1 | 5.35 (0.14 - 76.57) |
| La Unión (Nariño) | 3 | 18.81 (1.94 - 69.82) | 1 | 3.18 (0.08 - 66.89) | 0 | 0.00 (0.00 - 0.00) |
| La Unión (Sucre) | 0 | 0.00 (0.00 - 0.00) | 1 | 7.95 (0.20 - 149.17) | 2 | 2.22 (0.13 - 426.02) |
| La Unión (Valle del Cauca) | 2 | 4.90 (0.33 - 30.92) | 1 | 1.16 (0.03 - 20.95) | 0 | 0.00 (0.00 - 0.00) |
| La Uvita | 0 | 0.00 (0.00 - 0.00) | 0 | 0.00 (0.00 - 0.00) | 1 | 4.64 (0.12 - 666.14) |
| La Vega (Cauca) | 2 | 75.06 (2.24 - 280.68) | 0 | 0.00 (0.00 - 0.00) | 0 | 0.00 (0.00 - 0.00) |
| La Virginia | 10 | 53.29 (23.63 - 105.37) | 2 | 3.84 (0.18 - 43.05) | 2 | 4.89 (0.59 - 46.95) |
| Labateca | 0 | 0.00 (0.00 - 0.00) | 0 | 0.00 (0.00 - 0.00) | 1 | 57.09 (1.45 - 293.59) |
| Labranzagrande | 0 | 0.00 (0.00 - 0.00) | 1 | 665.88 (16.86 - 2545.84) | 0 | 0.00 (0.00 - 0.00) |
| Lebrija | 0 | 0.00 (0.00 - 0.00) | 0 | 0.00 (0.00 - 0.00) | 2 | 6.80 (0.60 - 31.48) |
| Leiva | 0 | 0.00 (0.00 - 0.00) | 1 | 228.94 (5.80 - 876.05) | 0 | 0.00 (0.00 - 0.00) |
| Lejanías | 1 | 5.59 (0.14 - 108.14) | 0 | 0.00 (0.00 - 0.00) | 0 | 0.00 (0.00 - 0.00) |
| Lérida | 0 | 0.00 (0.00 - 0.00) | 0 | 0.00 (0.00 - 0.00) | 1 | 21.47 (0.54 - 113.79) |
| Leticia | 1 | 1.83 (0.05 - 41.07) | 4 | 20.34 (3.93 - 63.98) | 1 | 2.79 (0.07 - 44.33) |
| Líbano | 0 | 0.00 (0.00 - 0.00) | 2 | 6.46 (0.71 - 29.56) | 1 | 0.15 (0.00 - 134.4) |
| Linares | 2 | 37.88 (1.72 - 171.19) | 1 | 1.29 (0.03 - 368.52) | 1 | 0.59 (0.02 - 239.12) |
| Lorica | 8 | 6.35 (2.07 - 17.35) | 7 | 4.20 (1.22 - 14.41) | 0 | 0.00 (0.00 - 0.00) |
| Los Córdobas | 1 | 15.15 (0.38 - 89.06) | 2 | 56.25 (3.25 - 220.21) | 0 | 0.00 (0.00 - 0.00) |
| Los Patios | 5 | 2.92 (0.91 - 17.38) | 0 | 0.00 (0.00 - 0.00) | 0 | 0.00 (0.00 - 0.00) |
| Luruaco | 2 | 18.31 (1.98 - 67.61) | 1 | 1.13 (0.03 – 39.00) | 0 | 0.00 (0.00 - 0.00) |
| Maceo | 1 | 4.79 (0.12 - 148.54) | 0 | 0.00 (0.00 - 0.00) | 1 | 8.73 (0.22 - 200.76) |
| Madrid | 4 | 20.60 (4.80 - 50.80) | 0 | 0.00 (0.00 - 0.00) | 1 | 0.79 (0.02 - 14.75) |
| Magangué | 9 | 20.74 (4.67 - 51.86) | 8 | 9.58 (3.33 - 21.87) | 4 | 0.35 (0.10 - 9.15) |
| Mahates | 4 | 9.71 (1.54 - 45.93) | 2 | 2.01 (0.20 - 82.53) | 1 | 0.53 (0.01 - 45.73) |
| Maicao | 6 | 7.78 (2.06 - 19.32) | 4 | 9.51 (1.04 - 29.15) | 4 | 3.69 (0.39 - 14.53) |
| Majagual | 0 | 0.00 (0.00 - 0.00) | 0 | 0.00 (0.00 - 0.00) | 1 | 3.53 (0.09 - 29.97) |
| Malambo | 6 | 11.72 (3.09 - 28.34) | 6 | 6.84 (2.07 - 17.86) | 1 | 1.13 (0.03 - 11.28) |
| Mallama | 0 | 0.00 (0.00 - 0.00) | 0 | 0.00 (0.00 - 0.00) | 1 | 26.06 (0.66 - 209.09) |
| Manatí | 3 | 30.34 (3.28 - 122.23) | 1 | 0.72 (0.02 - 103.65) | 0 | 0.00 (0.00 - 0.00) |
| Manaure | 1 | 3.70 (0.09 - 18.02) | 3 | 8.14 (1.05 - 24.77) | 0 | 0.00 (0.00 - 0.00) |
| Maní | 2 | 4.29 (0.36 - 219.18) | 0 | 0.00 (0.00 - 0.00) | 0 | 0.00 (0.00 - 0.00) |
| Manizales | 52 | 0.48 (0.33 - 5.51) | 13 | 1.68 (0.53 - 4.73) | 27 | 7.18 (4.31 - 11.5) |
| Manzanares | 1 | 2.17 (0.05 - 42.9) | 1 | 5.44 (0.14 - 48.41) | 0 | 0.00 (0.00 - 0.00) |
| Margarita | 1 | 9.79 (0.25 - 91.82) | 0 | 0.00 (0.00 - 0.00) | 0 | 0.00 (0.00 - 0.00) |
| María La Baja | 5 | 5.56 (1.50 - 25.21) | 4 | 58.77 (10.36 - 153.40) | 4 | 6.95 (1.47 - 29.49) |
| Marinilla | 3 | 1.07 (0.22 - 128.25) | 3 | 2.15 (0.44 - 23.40) | 5 | 10.79 (3.13 - 53.65) |
| Maripí | 0 | 0.00 (0.00 - 0.00) | 1 | 24.45 (0.62 - 207.60) | 0 | 0.00 (0.00 - 0.00) |
| Mariquita | 0 | 0.00 (0.00 - 0.00) | 1 | 0.29 (0.01 - 98.07) | 2 | 2.43 (0.27 - 30.38) |
| Marsella | 1 | 2.26 (0.06 - 118.49) | 0 | 0.00 (0.00 - 0.00) | 0 | 0.00 (0.00 - 0.00) |
| Marulanda | 1 | 9.11 (0.23 - 386.72) | 0 | 0.00 (0.00 - 0.00) | 0 | 0.00 (0.00 - 0.00) |
| Medellín | 550 | 34.73 (30.96 - 38.8) | 131 | 13.98 (8.61 - 20.7) | 324 | 20.57 (16.79 - 24.83) |
| Medina | 0 | 0.00 (0.00 - 0.00) | 0 | 0.00 (0.00 - 0.00) | 2 | 17.39 (0.74 - 101.99) |
| Melgar | 1 | 3.33 (0.08 - 32.48) | 1 | 0.15 (0.00 - 69.2) | 0 | 0.00 (0.00 - 0.00) |
| Mercaderes | 3 | 21.93 (2.94 - 159.32) | 0 | 0.00 (0.00 - 0.00) | 0 | 0.00 (0.00 - 0.00) |
| Milán | 0 | 0.00 (0.00 - 0.00) | 1 | 14.32 (0.36 - 177.13) | 0 | 0.00 (0.00 - 0.00) |
| Miraflores (Guaviare) | 0 | 0.00 (0.00 - 0.00) | 1 | 6.54 (0.17 - 106.63) | 0 | 0.00 (0.00 - 0.00) |
| Miranda | 4 | 8.09 (1.11 - 52.42) | 1 | 2.61 (0.07 - 26.65) | 2 | 2.78 (0.34 - 29.44) |
| Mistrató | 2 | 29.97 (3.63 - 121.7) | 0 | 0.00 (0.00 - 0.00) | 0 | 0.00 (0.00 - 0.00) |
| Mitú | 0 | 0.00 (0.00 - 0.00) | 1 | 11.23 (0.28 - 68.45) | 0 | 0.00 (0.00 - 0.00) |
| Mocoa | 2 | 6.65 (0.60 - 31.51) | 4 | 31.70 (1.31 - 114.66) | 1 | 10.44 (0.26 - 60.95) |
| Momil | 1 | 1.29 (0.03 - 56.4) | 1 | 1.32 (0.03 - 131.72) | 0 | 0.00 (0.00 - 0.00) |
| Mompós | 7 | 19.67 (6.86 - 52.46) | 2 | 1.73 (0.18 - 31.82) | 4 | 20.19 (3.64 - 58.36) |
| Monguí | 1 | 2.18 (0.06 - 271.96) | 0 | 0.00 (0.00 - 0.00) | 0 | 0.00 (0.00 - 0.00) |
| Moniquirá | 0 | 0.00 (0.00 - 0.00) | 2 | 5.10 (0.20 - 64.14) | 3 | 14.16 (2.76 - 69.24) |
| Montecristo | 0 | 0.00 (0.00 - 0.00) | 1 | 11.36 (0.29 - 75.45) | 0 | 0.00 (0.00 - 0.00) |
| Montelíbano | 3 | 5.54 (0.94 - 22.38) | 2 | 2.88 (0.35 - 25.91) | 0 | 0.00 (0.00 - 0.00) |
| Montenegro | 2 | 3.84 (0.42 - 33.48) | 0 | 0.00 (0.00 - 0.00) | 0 | 0.00 (0.00 - 0.00) |
| Montería | 52 | 34.08 (19.88 - 52.59) | 28 | 15.18 (6.34 - 28.68) | 22 | 16.91 (8.64 - 28.42) |
| Monterrey | 2 | 102.63 (4.21 - 373.40) | 1 | 4.59 (0.12 - 71.97) | 2 | 14.20 (1.57 - 77.69) |
| Moñitos | 1 | 0.33 (0.01 - 50.77) | 2 | 16.67 (1.07 - 74.03) | 2 | 4.63 (0.34 - 43.11) |
| Morales | 1 | 8.68 (0.22 - 71.92) | 0 | 0.00 (0.00 - 0.00) | 1 | 7.00 (0.18 - 50.02) |
| Morroa | 1 | 42.07 (1.07 - 209.81) | 0 | 0.00 (0.00 - 0.00) | 0 | 0.00 (0.00 - 0.00) |
| Mosquera (Cundinamarca) | 3 | 2.73 (0.42 - 12.24) | 0 | 0.00 (0.00 - 0.00) | 1 | 0.56 (0.01 - 30.68) |
| Mutatá | 0 | 0.00 (0.00 - 0.00) | 2 | 20.89 (1.19 - 101.25) | 0 | 0.00 (0.00 - 0.00) |
| Muzo | 1 | 77.64 (1.97 - 334.95) | 0 | 0.00 (0.00 - 0.00) | 1 | 3.39 (0.09 - 257.15) |
| Nariño (Nariño) | 1 | 4.47 (0.11 - 366.37) | 0 | 0.00 (0.00 - 0.00) | 0 | 0.00 (0.00 - 0.00) |
| Nechí | 1 | 11.33 (0.29 - 58.68) | 0 | 0.00 (0.00 - 0.00) | 0 | 0.00 (0.00 - 0.00) |
| Necoclí | 0 | 0.00 (0.00 - 0.00) | 1 | 9.06 (0.23 - 38.37) | 0 | 0.00 (0.00 - 0.00) |
| Neiva | 33 | 19.88 (12.65 - 29.40) | 15 | 8.38 (4.50 - 14.13) | 22 | 25.40 (13.50 - 41.88) |
| Nimaima | 1 | 22.49 (0.57 - 158.19) | 0 | 0.00 (0.00 - 0.00) | 1 | 78.19 (1.98 - 343.25) |
| Nobsa | 1 | 2.18 (0.06 - 56.79) | 1 | 1.15 (0.03 - 100) | 0 | 0.00 (0.00 - 0.00) |
| Nueva Granada | 2 | 15.71 (0.8 - 83.92) | 1 | 1.76 (0.04 - 41.35) | 0 | 0.00 (0.00 - 0.00) |
| Obando | 0 | 0.00 (0.00 - 0.00) | 0 | 0.00 (0.00 - 0.00) | 1 | 5.27 (0.13 - 90.95) |
| Ocaña | 7 | 17.66 (4.41 - 42.50) | 5 | 6.81 (1.61 - 19.06) | 2 | 3.67 (0.44 - 15.67) |
| Oiba | 0 | 0.00 (0.00 - 0.00) | 1 | 7.12 (0.18 - 78.41) | 0 | 0.00 (0.00 - 0.00) |
| Oicatá | 2 | 143.34 (8.65 - 636.04) | 0 | 0.00 (0.00 - 0.00) | 0 | 0.00 (0.00 - 0.00) |
| Olaya Herrera | 1 | 4.30 (0.11 - 42.88) | 0 | 0.00 (0.00 - 0.00) | 1 | 0.86 (0.02 - 27.76) |
| Orito | 1 | 13.20 (0.33 - 55.1) | 0 | 0.00 (0.00 - 0.00) | 0 | 0.00 (0.00 - 0.00) |
| Ortega | 1 | 0.02 (0.00 - 26.05) | 0 | 0.00 (0.00 - 0.00) | 1 | 2.22 (0.06 - 49.82) |
| Otanche | 1 | 17.29 (0.44 - 121.04) | 0 | 0.00 (0.00 - 0.00) | 0 | 0.00 (0.00 - 0.00) |
| Ovejas | 1 | 9.27 (0.23 - 59.66) | 0 | 0.00 (0.00 - 0.00) | 1 | 8.55 (0.22 - 55.26) |
| Pácora | 1 | 11.13 (0.28 - 87.56) | 0 | 0.00 (0.00 - 0.00) | 0 | 0.00 (0.00 - 0.00) |
| Padilla | 0 | 0.00 (0.00 - 0.00) | 0 | 0.00 (0.00 - 0.00) | 2 | 140.29 (13.36 - 449.55) |
| Pailitas | 2 | 2.29 (0.22 - 115.85) | 0 | 0.00 (0.00 - 0.00) | 0 | 0.00 (0.00 - 0.00) |
| Paime | 2 | 140.19 (7.08 - 704.14) | 0 | 0.00 (0.00 - 0.00) | 0 | 0.00 (0.00 - 0.00) |
| Paipa | 0 | 0.00 (0.00 - 0.00) | 0 | 0.00 (0.00 - 0.00) | 1 | 0.77 (0.02 - 47.92) |
| Palermo | 4 | 5.32 (1.15 - 36.09) | 0 | 0.00 (0.00 - 0.00) | 4 | 71.26 (9.57 - 203.62) |
| Palestina (Huila) | 1 | 10.18 (0.26 - 127.14) | 0 | 0.00 (0.00 - 0.00) | 1 | 8.03 (0.20 - 118.23) |
| Palmira | 40 | 12.59 (8.81 - 17.99) | 13 | 2.3 (0.88 - 8.08) | 26 | 1.62 (0.93 – 7.00) |
| Palmito | 1 | 62.88 (1.59 - 280.88) | 1 | 47.91 (1.21 - 217.08) | 0 | 0.00 (0.00 - 0.00) |
| Palocabildo | 0 | 0.00 (0.00 - 0.00) | 0 | 0.00 (0.00 - 0.00) | 1 | 22.69 (0.57 - 191.23) |
| Pamplona | 0 | 0.00 (0.00 - 0.00) | 1 | 0.15 (0.00 - 25.21) | 2 | 61.05 (2.66 – 216.00) |
| Pamplonita | 0 | 0.00 (0.00 - 0.00) | 0 | 0.00 (0.00 - 0.00) | 1 | 32.3 (0.82 - 238.48) |
| Panqueba | 0 | 0.00 (0.00 - 0.00) | 1 | 67.57 (1.71 - 773.42) | 0 | 0.00 (0.00 - 0.00) |
| Pasto | 57 | 19.68 (13.36 - 27.69) | 24 | 6.43 (1.97 - 13.94) | 21 | 6.12 (3.5 - 10.13) |
| Patía | 3 | 2.07 (0.30 - 54.81) | 0 | 0.00 (0.00 - 0.00) | 0 | 0.00 (0.00 - 0.00) |
| Paz de Ariporo | 4 | 11.11 (2.11 - 55.05) | 3 | 20.31 (3.58 - 80.46) | 1 | 0.57 (0.01 - 268.07) |
| Pelaya | 1 | 4.90 (0.12 - 69.49) | 0 | 0.00 (0.00 - 0.00) | 0 | 0.00 (0.00 - 0.00) |
| Pensilvania | 1 | 7.65 (0.19 - 50.55) | 1 | 1.38 (0.03 - 32.52) | 0 | 0.00 (0.00 - 0.00) |
| Peñol | 2 | 13.00 (1.07 - 72.35) | 1 | 7.30 (0.18 – 72.00) | 0 | 0.00 (0.00 - 0.00) |
| Peque | 1 | 1.88 (0.05 - 134.32) | 0 | 0.00 (0.00 - 0.00) | 1 | 4.23 (0.11 - 162.58) |
| Pereira | 77 | 26.27 (19.22 - 34.86) | 8 | 2.20 (0.74 - 5.16) | 26 | 12.23 (7.02 - 19.4) |
| Piedecuesta | 4 | 7.59 (1.14 - 21.63) | 1 | 0.12 (0.00 - 9.53) | 2 | 0.97 (0.09 - 8.05) |
| Piendamó | 0 | 0.00 (0.00 - 0.00) | 0 | 0.00 (0.00 - 0.00) | 1 | 6.64 (0.17 - 31.27) |
| Pijiño del Carmen | 1 | 6.03 (0.15 - 68.6) | 0 | 0.00 (0.00 - 0.00) | 0 | 0.00 (0.00 - 0.00) |
| Pinillos | 2 | 64.08 (5.60 - 217.25) | 1 | 2.75 (0.07 - 77.2) | 0 | 0.00 (0.00 - 0.00) |
| Pitalito | 0 | 0.00 (0.00 - 0.00) | 3 | 0.95 (0.19 - 9.92) | 2 | 5.61 (0.68 - 18.28) |
| Pivijay | 2 | 0.48 (0.04 - 60.04) | 2 | 1.67 (0.20 - 64.78) | 0 | 0.00 (0.00 - 0.00) |
| Planadas | 2 | 10.26 (0.29 - 132.35) | 0 | 0.00 (0.00 - 0.00) | 2 | 8.11 (0.76 - 49.09) |
| Planeta Rica | 4 | 17.67 (4.69 - 43.79) | 1 | 2.95 (0.07 - 22.15) | 4 | 10.64 (1.8 - 33.25) |
| Plato | 2 | 8.58 (1.04 – 32.00) | 3 | 4.51 (0.91 - 43.74) | 2 | 2.46 (0.22 - 16.72) |
| Policarpa | 0 | 0.00 (0.00 - 0.00) | 1 | 1.84 (0.05 - 68.71) | 0 | 0.00 (0.00 - 0.00) |
| Popayán | 22 | 16.11 (7.81 - 28.34) | 9 | 1.06 (0.25 - 7.58) | 16 | 2.22 (1.23 - 7.6) |
| Potosí | 0 | 0.00 (0.00 - 0.00) | 1 | 9.07 (0.23 - 140.02) | 0 | 0.00 (0.00 - 0.00) |
| Pradera | 0 | 0.00 (0.00 - 0.00) | 3 | 12.29 (2.33 - 38.49) | 2 | 1.49 (0.11 - 28.32) |
| Providencia (Nariño) | 1 | 0.75 (0.02 - 65.63) | 0 | 0.00 (0.00 - 0.00) | 0 | 0.00 (0.00 - 0.00) |
| Providencia (San Andrés) | 0 | 0.00 (0.00 - 0.00) | 0 | 0.00 (0.00 - 0.00) | 1 | 212.59 (5.38 - 841.84) |
| Pueblo Nuevo | 1 | 5.30 (0.13 - 42.4) | 2 | 67.14 (2.34 - 248.25) | 1 | 2.09 (0.05 - 29.37) |
| Puerto Asís | 2 | 4.60 (0.56 - 45.49) | 0 | 0.00 (0.00 - 0.00) | 1 | 3.59 (0.09 - 19.79) |
| Puerto Berrío | 1 | 5.84 (0.15 - 28.89) | 0 | 0.00 (0.00 - 0.00) | 0 | 0.00 (0.00 - 0.00) |
| Puerto Boyacá | 0 | 0.00 (0.00 - 0.00) | 2 | 8.69 (0.49 - 35.63) | 1 | 0.39 (0.01 - 34.75) |
| Puerto Caicedo | 0 | 0.00 (0.00 - 0.00) | 1 | 10.33 (0.26 - 135.71) | 0 | 0.00 (0.00 - 0.00) |
| Puerto Colombia (Atlántico) | 2 | 8.71 (0.84 - 54.46) | 0 | 0.00 (0.00 - 0.00) | 2 | 7.13 (0.82 - 39.59) |
| Puerto Escondido | 2 | 2.77 (0.24 - 43.78) | 2 | 17.5 (1.03 - 76.33) | 0 | 0.00 (0.00 - 0.00) |
| Puerto Gaitán | 1 | 3.18 (0.08 - 83.88) | 0 | 0.00 (0.00 - 0.00) | 0 | 0.00 (0.00 - 0.00) |
| Puerto Guzmán | 1 | 5.92 (0.15 - 377.69) | 0 | 0.00 (0.00 - 0.00) | 0 | 0.00 (0.00 - 0.00) |
| Puerto Libertador | 3 | 11.31 (2.17 - 38.01) | 2 | 1.91 (0.21 - 27.14) | 0 | 0.00 (0.00 - 0.00) |
| Puerto López | 2 | 7.69 (0.66 - 38.95) | 0 | 0.00 (0.00 - 0.00) | 0 | 0.00 (0.00 - 0.00) |
| Puerto Nariño | 0 | 0.00 (0.00 - 0.00) | 1 | 1.65 (0.04 - 206.6) | 0 | 0.00 (0.00 - 0.00) |
| Puerto Rico (Caquetá) | 2 | 11.92 (1.36 - 56.44) | 1 | 3.26 (0.08 - 38.27) | 1 | 9.98 (0.25 - 89.29) |
| Puerto Rico (Meta) | 0 | 0.00 (0.00 - 0.00) | 0 | 0.00 (0.00 - 0.00) | 1 | 6.69 (0.17 - 113.71) |
| Puerto Santander (Norte de Santander) | 1 | 39.70 (1.01 - 213.59) | 1 | 22.28 (0.56 - 168.2) | 0 | 0.00 (0.00 - 0.00) |
| Puerto Tejada | 3 | 6.11 (1.13 - 36.89) | 1 | 0.85 (0.02 - 70.13) | 4 | 11.9 (2.34 - 37.43) |
| Puerto Triunfo | 0 | 0.00 (0.00 - 0.00) | 1 | 4.37 (0.11 - 129) | 1 | 2.92 (0.07 - 41.43) |
| Puerto Wilches | 4 | 42.22 (6.98 - 150.36) | 1 | 1.43 (0.04 - 40.27) | 0 | 0.00 (0.00 - 0.00) |
| Pulí | 0 | 0.00 (0.00 - 0.00) | 0 | 0.00 (0.00 - 0.00) | 1 | 24.27 (0.61 - 341.43) |
| Pupiales | 1 | 9.78 (0.25 - 62.77) | 1 | 5.21 (0.13 - 48.78) | 0 | 0.00 (0.00 - 0.00) |
| Purificación | 3 | 8.04 (1.39 - 38.33) | 1 | 1.55 (0.04 - 32.25) | 0 | 0.00 (0.00 - 0.00) |
| Purísima | 2 | 6.61 (0.80 - 58.44) | 1 | 9.81 (0.25 - 75.08) | 0 | 0.00 (0.00 - 0.00) |
| Quibdó | 5 | 10.65 (3.04 - 25.49) | 5 | 10.71 (3.00 - 25.58) | 7 | 4.60 (1.60 - 13.86) |
| Quimbaya | 1 | 2.20 (0.06 - 38.24) | 0 | 0.00 (0.00 - 0.00) | 0 | 0.00 (0.00 - 0.00) |
| Quinchía | 1 | 4.46 (0.11 - 31.56) | 0 | 0.00 (0.00 - 0.00) | 0 | 0.00 (0.00 - 0.00) |
| Quípama | 0 | 0.00 (0.00 - 0.00) | 0 | 0.00 (0.00 - 0.00) | 1 | 18.72 (0.47 - 134.58) |
| Quipile | 0 | 0.00 (0.00 - 0.00) | 0 | 0.00 (0.00 - 0.00) | 1 | 14.63 (0.37 - 305.53) |
| Ragonvalia | 0 | 0.00 (0.00 - 0.00) | 0 | 0.00 (0.00 - 0.00) | 1 | 30.28 (0.77 - 186.68) |
| Ramiriquí | 1 | 12.67 (0.32 - 234.87) | 0 | 0.00 (0.00 - 0.00) | 0 | 0.00 (0.00 - 0.00) |
| Remedios | 0 | 0.00 (0.00 - 0.00) | 2 | 21.32 (1.99 - 74.94) | 0 | 0.00 (0.00 - 0.00) |
| Remolino | 0 | 0.00 (0.00 - 0.00) | 1 | 56.4 (1.43 - 248.93) | 0 | 0.00 (0.00 - 0.00) |
| Repelón | 0 | 0.00 (0.00 - 0.00) | 0 | 0.00 (0.00 - 0.00) | 2 | 19.07 (0.81 - 81.4) |
| Restrepo (Meta) | 0 | 0.00 (0.00 - 0.00) | 1 | 41.08 (1.04 - 175.24) | 0 | 0.00 (0.00 - 0.00) |
| Restrepo (Valle del Cauca) | 2 | 1.51 (0.16 - 81.75) | 0 | 0.00 (0.00 - 0.00) | 0 | 0.00 (0.00 - 0.00) |
| Retiro | 1 | 5.09 (0.13 - 57.33) | 3 | 48.34 (3.47 - 181.7) | 7 | 64.57 (21.55 - 148.81) |
| Ricaurte (Nariño) | 1 | 1.72 (0.04 - 55.14) | 0 | 0.00 (0.00 - 0.00) | 0 | 0.00 (0.00 - 0.00) |
| Río de Oro | 0 | 0.00 (0.00 - 0.00) | 1 | 0.68 (0.02 - 203.04) | 0 | 0.00 (0.00 - 0.00) |
| Río Iro | 0 | 0.00 (0.00 - 0.00) | 0 | 0.00 (0.00 - 0.00) | 1 | 1.30 (0.03 - 93.51) |
| Río Viejo | 1 | 11.13 (0.28 - 62.65) | 0 | 0.00 (0.00 - 0.00) | 0 | 0.00 (0.00 - 0.00) |
| Rioblanco | 0 | 0.00 (0.00 - 0.00) | 0 | 0.00 (0.00 - 0.00) | 2 | 16.84 (2.04 - 94.65) |
| Riohacha | 16 | 27.95 (9.91 - 57.36) | 1 | 0.15 (0.00 - 8.81) | 7 | 6.98 (1.99 - 16.24) |
| Rionegro (Antioquia) | 23 | 42.49 (25.18 - 66.73) | 4 | 3.15 (0.66 - 12.39) | 21 | 32.81 (16.67 - 56.58) |
| Rionegro (Santander) | 2 | 4.60 (0.46 - 37.21) | 0 | 0.00 (0.00 - 0.00) | 2 | 24.90 (2.98 - 84.73) |
| Rivera | 4 | 31.30 (7.64 - 94.25) | 0 | 0.00 (0.00 - 0.00) | 1 | 9.16 (0.23 - 64.26) |
| Roberto Payán | 0 | 0.00 (0.00 - 0.00) | 2 | 12.94 (1.05 - 97.74) | 0 | 0.00 (0.00 - 0.00) |
| Roldanillo | 2 | 1.61 (0.19 - 31.39) | 0 | 0.00 (0.00 - 0.00) | 0 | 0.00 (0.00 - 0.00) |
| Rondón | 1 | 23.78 (0.60 - 434.91) | 0 | 0.00 (0.00 - 0.00) | 0 | 0.00 (0.00 - 0.00) |
| Sabana de Torres | 0 | 0.00 (0.00 - 0.00) | 1 | 3.58 (0.09 - 141.56) | 0 | 0.00 (0.00 - 0.00) |
| Sabanagrande | 0 | 0.00 (0.00 - 0.00) | 1 | 10.57 (0.27 - 49.03) | 2 | 15.85 (1.18 - 61.4) |
| Sabanalarga (Antioquia) | 2 | 25.58 (3.01 - 153.41) | 1 | 293.25 (7.42 - 1122.30) | 2 | 14.15 (1.11 - 234.11) |
| Sabanalarga (Atlántico) | 4 | 2.10 (0.54 - 12.99) | 2 | 2.44 (0.30 - 18.87) | 1 | 4.45 (0.11 - 20.34) |
| Sabanalarga (Casanare) | 1 | 68.86 (1.74 - 445.76) | 0 | 0.00 (0.00 - 0.00) | 0 | 0.00 (0.00 - 0.00) |
| Sabanas de San Ángel | 1 | 7.81 (0.20 - 62.12) | 0 | 0.00 (0.00 - 0.00) | 0 | 0.00 (0.00 - 0.00) |
| Sabaneta | 25 | 65.08 (34.6 - 110.07) | 5 | 27.81 (1.17 - 100.37) | 14 | 19.19 (9.56 - 45.43) |
| Saboyá | 1 | 9.53 (0.24 - 103.03) | 0 | 0.00 (0.00 - 0.00) | 1 | 2.25 (0.06 - 113.07) |
| Sácama | 0 | 0.00 (0.00 - 0.00) | 1 | 5.32 (0.13 - 447.71) | 0 | 0.00 (0.00 - 0.00) |
| Sáchica | 1 | 2.34 (0.06 - 377.21) | 0 | 0.00 (0.00 - 0.00) | 0 | 0.00 (0.00 - 0.00) |
| Sahagún | 10 | 32.42 (6.95 - 80.26) | 2 | 4.61 (0.28 - 21.24) | 1 | 3.29 (0.08 - 16.44) |
| Saladoblanco | 1 | 83.16 (2.11 - 335.9) | 0 | 0.00 (0.00 - 0.00) | 0 | 0.00 (0.00 - 0.00) |
| Salamina | 0 | 0.00 (0.00 - 0.00) | 0 | 0.00 (0.00 - 0.00) | 3 | 114.73 (21.42 - 332.79) |
| Salamina | 0 | 0.00 (0.00 - 0.00) | 0 | 0.00 (0.00 - 0.00) | 1 | 6.96 (0.18 - 66.53) |
| Saldaña | 1 | 19.85 (0.50 - 107.47) | 0 | 0.00 (0.00 - 0.00) | 0 | 0.00 (0.00 - 0.00) |
| Salgar | 2 | 8.10 (0.58 - 76.66) | 0 | 0.00 (0.00 - 0.00) | 0 | 0.00 (0.00 - 0.00) |
| Samacá | 2 | 13.5 (1.44 - 64.75) | 0 | 0.00 (0.00 - 0.00) | 1 | 5.98 (0.15 - 56.66) |
| Samaniego | 1 | 3.03 (0.08 - 42.60) | 1 | 1.54 (0.04 - 18.31) | 3 | 3.82 (0.41 - 31.43) |
| Sampués | 1 | 2.88 (0.07 - 28.43) | 2 | 5.87 (0.71 - 34.36) | 0 | 0.00 (0.00 - 0.00) |
| San Agustín | 1 | 0.36 (0.01 - 43.55) | 2 | 39.55 (1.57 - 151.76) | 1 | 9.98 (0.25 - 49.23) |
| San Alberto | 0 | 0.00 (0.00 - 0.00) | 1 | 3.49 (0.09 - 40.16) | 0 | 0.00 (0.00 - 0.00) |
| San Andrés | 3 | 4.76 (0.9 - 19.43) | 0 | 0.00 (0.00 - 0.00) | 1 | 9.75 (0.25 - 116.66) |
| San Andrés | 0 | 0.00 (0.00 - 0.00) | 0 | 0.00 (0.00 - 0.00) | 1 | 1.12 (0.03 - 22.65) |
| San Andres de Tumaco | 12 | 14.30 (4.40 - 42.49) | 7 | 12.97 (0.82 - 43.21) | 7 | 104.16 (13.61 - 290.44) |
| San Andrés Sotavento | 3 | 26.01 (3.65 - 82.73) | 0 | 0.00 (0.00 - 0.00) | 2 | 15.89 (0.78 - 62.93) |
| San Antero | 4 | 27.85 (3.96 - 84.37) | 1 | 2.81 (0.07 - 41.04) | 1 | 0.10 (0.00 - 50.37) |
| San Bernardo | 0 | 0.00 (0.00 - 0.00) | 0 | 0.00 (0.00 - 0.00) | 1 | 2.12 (0.05 - 65.71) |
| San Bernardo del Viento | 3 | 9.86 (1.05 - 48.21) | 1 | 0.39 (0.01 - 39.64) | 0 | 0.00 (0.00 - 0.00) |
| San Carlos de Guaroa | 1 | 178.25 (4.51 - 696.59) | 1 | 38.98 (0.99 - 210.23) | 0 | 0.00 (0.00 - 0.00) |
| San Cristóbal | 1 | 48.19 (1.22 - 262.15) | 0 | 0.00 (0.00 - 0.00) | 3 | 43.78 (8.98 - 292.09) |
| San Diego | 2 | 47.88 (1.74 - 207.32) | 0 | 0.00 (0.00 - 0.00) | 1 | 5.84 (0.15 - 108.65) |
| San Eduardo | 0 | 0.00 (0.00 - 0.00) | 0 | 0.00 (0.00 - 0.00) | 1 | 227.56 (5.76 - 1118.85) |
| San Estanislao | 1 | 8.47 (0.21 - 69.67) | 3 | 7.81 (1.33 - 66.71) | 1 | 1.78 (0.05 - 53.42) |
| San Gil | 1 | 0.97 (0.02 - 23.71) | 0 | 0.00 (0.00 - 0.00) | 2 | 0.09 (0.01 - 31.40) |
| San Jacinto | 1 | 13.27 (0.34 - 70.26) | 1 | 24.66 (0.62 - 103.87) | 3 | 11.43 (2.22 - 56.38) |
| San Jacinto del Cauca | 1 | 8.49 (0.22 - 93.11) | 0 | 0.00 (0.00 - 0.00) | 0 | 0.00 (0.00 - 0.00) |
| San Jerónimo | 1 | 6.48 (0.16 - 147.70) | 0 | 0.00 (0.00 - 0.00) | 0 | 0.00 (0.00 - 0.00) |
| San José de Miranda | 0 | 0.00 (0.00 - 0.00) | 0 | 0.00 (0.00 - 0.00) | 1 | 15.7 (0.40 - 217.46) |
| San José de Pare | 1 | 4.21 (0.11 - 341.56) | 0 | 0.00 (0.00 - 0.00) | 0 | 0.00 (0.00 - 0.00) |
| San José del Guaviare | 1 | 1.30 (0.03 - 37.19) | 2 | 4.95 (0.55 - 48.58) | 1 | 6.78 (0.17 - 34.76) |
| San Juan de Betulia | 1 | 2.78 (0.07 - 121.73) | 1 | 10.97 (0.28 - 132.04) | 2 | 51.17 (6.20 - 171.81) |
| San Juan de Urabá | 0 | 0.00 (0.00 - 0.00) | 3 | 2.65 (0.50 - 40.80) | 0 | 0.00 (0.00 - 0.00) |
| San Juan del Cesar | 3 | 2.26 (0.47 - 46.8) | 1 | 7.49 (0.19 - 47.61) | 0 | 0.00 (0.00 - 0.00) |
| San Juan Nepomuceno | 3 | 0.37 (0.03 - 45.58) | 1 | 1.76 (0.04 - 53.08) | 1 | 2.53 (0.06 - 45.83) |
| San Lorenzo | 1 | 0.17 (0.00- 135.95) | 0 | 0.00 (0.00 - 0.00) | 1 | 5.02 (0.13 - 80.78) |
| San Luis | 0 | 0.00 (0.00 - 0.00) | 0 | 0.00 (0.00 - 0.00) | 1 | 8.25 (0.21 - 88.99) |
| San Luis de Palenque | 0 | 0.00 (0.00 - 0.00) | 1 | 20.20 (0.51 - 222.03) | 0 | 0.00 (0.00 - 0.00) |
| San Luis de Sincé | 1 | 0.40 (0.01 - 28.88) | 1 | 2.28 (0.06 - 26.56) | 1 | 2.32 (0.06 - 40.32) |
| San Marcos | 2 | 0.15 (0.01 - 36.12) | 4 | 3.75 (0.91 - 26.46) | 0 | 0.00 (0.00 - 0.00) |
| San Martín de Loba | 0 | 0.00 (0.00 - 0.00) | 1 | 0.82 (0.02 - 56.43) | 0 | 0.00 (0.00 - 0.00) |
| San Miguel de Sema | 1 | 3.54 (0.09 - 282.32) | 0 | 0.00 (0.00 - 0.00) | 0 | 0.00 (0.00 - 0.00) |
| San Onofre | 4 | 10.16 (2.57 - 40.13) | 1 | 0.33 (0.01 - 43.12) | 2 | 0.60 (0.05 - 26.35) |
| San Pablo | 3 | 45.14 (8.09 - 136.45) | 1 | 4.68 (0.12 - 57.15) | 1 | 3.33 (0.08 - 68.31) |
| San Pablo de Borbur | 1 | 1.03 (0.03 - 114.20) | 0 | 0.00 (0.00 - 0.00) | 0 | 0.00 (0.00 - 0.00) |
| San Pedro | 4 | 30.29 (6.61 - 101.45) | 2 | 32.92 (3.84 - 116.27) | 1 | 3.58 (0.09 - 49.03) |
| San Pedro | 1 | 6.83 (0.17 - 47.10) | 0 | 0.00 (0.00 - 0.00) | 0 | 0.00 (0.00 - 0.00) |
| San Pedro de Urabá | 0 | 0.00 (0.00 - 0.00) | 2 | 9.34 (1.13 - 58.76) | 0 | 0.00 (0.00 - 0.00) |
| San Pelayo | 1 | 0.44 (0.01 - 20.54) | 1 | 1.60 (0.04 - 32.49) | 2 | 11.96 (1.42 - 42.21) |
| San Rafael | 1 | 4.63 (0.12 - 80.80) | 0 | 0.00 (0.00 - 0.00) | 0 | 0.00 (0.00 - 0.00) |
| San Roque | 1 | 4.49 (0.11 - 52.80) | 0 | 0.00 (0.00 - 0.00) | 0 | 0.00 (0.00 - 0.00) |
| San Sebastián | 0 | 0.00 (0.00 - 0.00) | 1 | 17.83 (0.45 - 99.33) | 0 | 0.00 (0.00 - 0.00) |
| San Sebastián de Buenavista | 0 | 0.00 (0.00 - 0.00) | 1 | 4.77 (0.12 - 60.45) | 0 | 0.00 (0.00 - 0.00) |
| San Vicente | 1 | 248.16 (6.28 - 951.73) | 0 | 0.00 (0.00 - 0.00) | 0 | 0.00 (0.00 - 0.00) |
| San Vicente de Chucurí | 1 | 0.11 (0.00 - 35.47) | 0 | 0.00 (0.00 - 0.00) | 0 | 0.00 (0.00 - 0.00) |
| San Vicente del Caguán | 1 | 1.89 (0.05 - 15.6) | 0 | 0.00 (0.00 - 0.00) | 1 | 0.33 (0.01 - 29.96) |
| San Zenón | 0 | 0.00 (0.00 - 0.00) | 1 | 1.66 (0.04 - 98.92) | 1 | 12.68 (0.32 - 110.68) |
| Sandoná | 3 | 132.65 (4.99 - 479.34) | 0 | 0.00 (0.00 - 0.00) | 1 | 5.57 (0.14 - 42.41) |
| Santa Ana | 2 | 7.03 (0.83 - 54.10) | 0 | 0.00 (0.00 - 0.00) | 1 | 5.66 (0.14 - 89.48) |
| Santa Bárbara | 1 | 3.75 (0.09 - 50.62) | 0 | 0.00 (0.00 - 0.00) | 1 | 5.61 (0.14 - 43.10) |
| Santa Bárbara de Pinto | 1 | 70.87 (1.79 - 349.15) | 0 | 0.00 (0.00 - 0.00) | 0 | 0.00 (0.00 - 0.00) |
| Santa Catalina | 0 | 0.00 (0.00 - 0.00) | 1 | 16.00 (0.41 - 107.42) | 3 | 55.94 (6.91 – 210.00) |
| Santa Lucía | 1 | 5.76 (0.15 - 107.50) | 0 | 0.00 (0.00 - 0.00) | 0 | 0.00 (0.00 - 0.00) |
| Santa Marta | 51 | 20.33 (12.53 - 30.54) | 13 | 0.55 (0.26 - 2.36) | 20 | 3.72 (2.13 - 7.51) |
| Santa Rosa | 0 | 0.00 (0.00 - 0.00) | 1 | 19.30 (0.49 – 89.00) | 1 | 8.15 (0.21 - 47.67) |
| Santa Rosa | 0 | 0.00 (0.00 - 0.00) | 0 | 0.00 (0.00 - 0.00) | 1 | 1.11 (0.03 - 147.39) |
| Santa Rosa de Cabal | 5 | 73.15 (11.30 - 195.77) | 0 | 0.00 (0.00 - 0.00) | 2 | 1.56 (0.15 - 12.27) |
| Santa Rosa de Osos | 3 | 9.27 (0.75 - 49.74) | 1 | 0.85 (0.02 - 31.95) | 1 | 1.61 (0.04 - 35.37) |
| Santa Rosa del Sur | 0 | 0.00 (0.00 - 0.00) | 1 | 0.02 (0.00 - 213.24) | 0 | 0.00 (0.00 - 0.00) |
| Santafé de Antioquia | 2 | 4.49 (0.54 - 70.6) | 0 | 0.00 (0.00 - 0.00) | 1 | 50.02 (1.27 - 198.25) |
| Santander de Quilichao | 8 | 12.12 (4.77 - 26.22) | 2 | 1.1 (0.11 - 12.68) | 2 | 1.81 (0.20 - 12.81) |
| Santiago | 0 | 0.00 (0.00 - 0.00) | 1 | 18.96 (0.48 - 164.07) | 0 | 0.00 (0.00 - 0.00) |
| Santiago de Tolú | 0 | 0.00 (0.00 - 0.00) | 1 | 37.8 (0.96 - 147.72) | 1 | 2.53 (0.06 - 26.44) |
| Santo Domingo | 1 | 26.84 (0.68 - 142.96) | 0 | 0.00 (0.00 - 0.00) | 0 | 0.00 (0.00 - 0.00) |
| Santo Tomás | 0 | 0.00 (0.00 - 0.00) | 1 | 3.31 (0.08 - 59.98) | 2 | 3.15 (0.26 - 40.31) |
| Santuario | 1 | 10.86 (0.27 - 73.16) | 0 | 0.00 (0.00 - 0.00) | 0 | 0.00 (0.00 - 0.00) |
| Saravena | 1 | 5.81 (0.15 - 29.40) | 3 | 16.34 (1.23 - 57.80) | 0 | 0.00 (0.00 - 0.00) |
| Sardinata | 2 | 23.29 (2.80 - 88.58) | 0 | 0.00 (0.00 - 0.00) | 0 | 0.00 (0.00 - 0.00) |
| Segovia | 3 | 13.39 (2.59 - 76.06) | 1 | 4.87 (0.12 - 33.57) | 0 | 0.00 (0.00 - 0.00) |
| Sesquilé | 1 | 200.91 (5.09 - 771) | 0 | 0.00 (0.00 - 0.00) | 0 | 0.00 (0.00 - 0.00) |
| Sevilla | 2 | 0.60 (0.07 - 29.16) | 0 | 0.00 (0.00 - 0.00) | 1 | 2.08 (0.05 - 20.15) |
| Sibaté | 1 | 1.79 (0.05 - 27.89) | 0 | 0.00 (0.00 - 0.00) | 0 | 0.00 (0.00 - 0.00) |
| Sibundoy | 1 | 9.35 (0.24 - 78.61) | 1 | 1.15 (0.03 - 67.90) | 0 | 0.00 (0.00 - 0.00) |
| Silvia | 1 | 1.56 (0.04 - 30.69) | 0 | 0.00 (0.00 - 0.00) | 0 | 0.00 (0.00 - 0.00) |
| Simacota | 0 | 0.00 (0.00 - 0.00) | 0 | 0.00 (0.00 - 0.00) | 1 | 2.40 (0.06 - 120.30) |
| Simití | 3 | 44.13 (2.83 - 154.70) | 1 | 1.79 (0.05 - 69.15) | 0 | 0.00 (0.00 - 0.00) |
| Sincelejo | 21 | 8.22 (2.79 - 17.34) | 6 | 3.92 (0.79 - 11.20) | 18 | 7.92 (4.02 - 14.76) |
| Sitionuevo | 0 | 0.00 (0.00 - 0.00) | 0 | 0.00 (0.00 - 0.00) | 1 | 2.53 (0.06 - 46.74) |
| Soacha | 17 | 7.95 (4.14 - 13.50) | 9 | 2.55 (1.00 - 5.79) | 12 | 4.21 (1.99 - 7.78) |
| Socorro | 0 | 0.00 (0.00 - 0.00) | 0 | 0.00 (0.00 - 0.00) | 1 | 52.53 (1.33 - 204.12) |
| Sogamoso | 8 | 16.36 (1.33 - 52.59) | 5 | 8.06 (1.29 - 23.22) | 11 | 17.62 (6.31 - 37.86) |
| Solano | 0 | 0.00 (0.00 - 0.00) | 1 | 12.7 (0.32 - 65.57) | 0 | 0.00 (0.00 - 0.00) |
| Soledad | 35 | 12.17 (7.86 - 17.8) | 7 | 1.55 (0.35 - 4.32) | 11 | 1.40 (0.53 - 3.58) |
| Solita | 0 | 0.00 (0.00 - 0.00) | 2 | 18.88 (1.82 - 175.41) | 0 | 0.00 (0.00 - 0.00) |
| Sonson | 1 | 25.01 (0.63 - 116.27) | 0 | 0.00 (0.00 - 0.00) | 1 | 0.13 (0.00 - 68.18) |
| Sopetrán | 1 | 27.64 (0.70 - 136.16) | 1 | 1.78 (0.05 - 97.28) | 0 | 0.00 (0.00 - 0.00) |
| Soplaviento | 1 | 4.22 (0.11 - 158.4) | 0 | 0.00 (0.00 - 0.00) | 0 | 0.00 (0.00 - 0.00) |
| Sotara | 0 | 0.00 (0.00 - 0.00) | 1 | 3.91 (0.10 - 54.21) | 0 | 0.00 (0.00 - 0.00) |
| Suárez | 0 | 0.00 (0.00 - 0.00) | 1 | 0.97 (0.02 - 403.99) | 1 | 33.94 (0.86 - 236.46) |
| Suaza | 1 | 23.35 (0.59 - 105.37) | 0 | 0.00 (0.00 - 0.00) | 0 | 0.00 (0.00 - 0.00) |
| Subachoque | 1 | 55.400 (1.40 - 221.24) | 0 | 0.00 (0.00 - 0.00) | 0 | 0.00 (0.00 - 0.00) |
| Sucre | 1 | 46.32 (1.17 - 220.91) | 0 | 0.00 (0.00 - 0.00) | 0 | 0.00 (0.00 - 0.00) |
| Sucre | 1 | 14.43 (0.37 - 70.10) | 0 | 0.00 (0.00 - 0.00) | 0 | 0.00 (0.00 - 0.00) |
| Supía | 2 | 11.92 (0.64 - 57.60) | 2 | 1.34 (0.09 - 67.01) | 0 | 0.00 (0.00 - 0.00) |
| Tabio | 0 | 0.00 (0.00 - 0.00) | 0 | 0.00 (0.00 - 0.00) | 1 | 7.78 (0.20 - 45.89) |
| Tadó | 1 | 3.85 (0.10 - 83.50) | 2 | 71.12 (7.66 - 228.82) | 0 | 0.00 (0.00 - 0.00) |
| Támara | 0 | 0.00 (0.00 - 0.00) | 2 | 68.51 (7.87 - 282.14) | 0 | 0.00 (0.00 - 0.00) |
| Tame | 1 | 2.39 (0.06 - 22.49) | 0 | 0.00 (0.00 - 0.00) | 0 | 0.00 (0.00 - 0.00) |
| Támesis | 1 | 3.86 (0.10 - 77.69) | 1 | 26.70 (0.68 - 127.13) | 0 | 0.00 (0.00 - 0.00) |
| Taminango | 2 | 8.06 (0.53 - 66.06) | 1 | 2.76 (0.07 - 86.46) | 1 | 0.98 (0.02 - 40.11) |
| Tangua | 0 | 0.00 (0.00 - 0.00) | 1 | 13.21 (0.33 - 112.06) | 1 | 10.33 (0.26 - 106.69) |
| Tarazá | 1 | 1.70 (0.04 - 165.15) | 2 | 0.35 (0.03 - 27.37) | 1 | 4.11 (0.10 - 63.62) |
| Tarqui | 1 | 2.48 (0.06 - 113.99) | 0 | 0.00 (0.00 - 0.00) | 0 | 0.00 (0.00 - 0.00) |
| Tauramena | 1 | 0.67 (0.02 - 69.81) | 2 | 9.25 (0.80 - 61.04) | 0 | 0.00 (0.00 - 0.00) |
| Tello | 0 | 0.00 (0.00 - 0.00) | 3 | 6.10 (0.80 - 96.62) | 1 | 3.72 (0.09 - 61.98) |
| Tenerife | 1 | 107.46 (2.72 - 421.51) | 0 | 0.00 (0.00 - 0.00) | 0 | 0.00 (0.00 - 0.00) |
| Teorama | 2 | 23.16 (2.40 - 91.34) | 0 | 0.00 (0.00 - 0.00) | 0 | 0.00 (0.00 - 0.00) |
| Teruel | 0 | 0.00 (0.00 - 0.00) | 0 | 0.00 (0.00 - 0.00) | 1 | 3.45 (0.09 - 132.78) |
| Tesalia | 2 | 12.11 (1.28 - 116.25) | 0 | 0.00 (0.00 - 0.00) | 0 | 0.00 (0.00 - 0.00) |
| Tibaná | 1 | 41.06 (1.04 - 198.69) | 0 | 0.00 (0.00 - 0.00) | 0 | 0.00 (0.00 - 0.00) |
| Tibasosa | 1 | 6.13 (0.16 - 162.20) | 0 | 0.00 (0.00 - 0.00) | 0 | 0.00 (0.00 - 0.00) |
| Tibú | 1 | 10.15 (0.26 - 62.62) | 1 | 1.38 (0.04 - 35.47) | 1 | 6.65 (0.17 - 41.16) |
| Tierralta | 1 | 0.02 (0.00 - 62.56) | 5 | 4.23 (1.13 - 35.37) | 2 | 5.26 (0.19 - 25.03) |
| Timbío | 0 | 0.00 (0.00 - 0.00) | 2 | 6.96 (0.81 - 51.22) | 1 | 0.17 (0.00 - 49.84) |
| Tipacoque | 1 | 26.14 (0.66 - 347.25) | 0 | 0.00 (0.00 - 0.00) | 0 | 0.00 (0.00 - 0.00) |
| Titiribí | 1 | 3.39 (0.09 - 87.93) | 1 | 7.41 (0.19 - 75.74) | 0 | 0.00 (0.00 - 0.00) |
| Toca | 1 | 2.55 (0.06 - 214.44) | 0 | 0.00 (0.00 - 0.00) | 0 | 0.00 (0.00 - 0.00) |
| Tocancipá | 2 | 4.56 (0.55 - 30.07) | 2 | 1.22 (0.08 - 40.10) | 2 | 3.15 (0.32 - 61.30) |
| Toledo | 1 | 13.16 (0.33 - 74.23) | 1 | 0.10 (0.00 - 183.73) | 1 | 2.63 (0.07 - 52.97) |
| Tolú Viejo | 1 | 29.54 (0.75 - 126.95) | 0 | 0.00 (0.00 - 0.00) | 3 | 34.92 (5.19 - 110.86) |
| Tona | 1 | 22.86 (0.58 - 189.80) | 0 | 0.00 (0.00 - 0.00) | 0 | 0.00 (0.00 - 0.00) |
| Tópaga | 1 | 56.12 (1.42 - 322.04) | 0 | 0.00 (0.00 - 0.00) | 0 | 0.00 (0.00 - 0.00) |
| Tota | 1 | 51.42 (1.30 - 388.65) | 0 | 0.00 (0.00 - 0.00) | 0 | 0.00 (0.00 - 0.00) |
| Trujillo | 1 | 1.18 (0.03 - 59.11) | 0 | 0.00 (0.00 - 0.00) | 0 | 0.00 (0.00 - 0.00) |
| Tubará | 2 | 16.98 (2.02 - 101.46) | 0 | 0.00 (0.00 - 0.00) | 0 | 0.00 (0.00 - 0.00) |
| Tuchín | 3 | 37.47 (4.99 - 110.50) | 0 | 0.00 (0.00 - 0.00) | 1 | 3.18 (0.08 - 82.43) |
| Tuluá | 23 | 24.45 (8.31 - 50.11) | 7 | 4.71 (1.53 - 11.24) | 4 | 1.08 (0.29 - 5.86) |
| Tunja | 18 | 6.16 (2.73 - 13.77) | 8 | 7.92 (1.86 - 19.77) | 8 | 3.99 (1.29 - 11.14) |
| Túquerres | 3 | 6.99 (0.68 - 49.70) | 2 | 3.65 (0.42 - 33.02) | 0 | 0.00 (0.00 - 0.00) |
| Turbaco | 17 | 4.11 (2.20 - 20.22) | 2 | 10.31 (1.02 - 33.71) | 5 | 16.81 (5.45 - 39.12) |
| Turbaná | 1 | 7.31 (0.19 - 74.04) | 1 | 11.13 (0.28 - 357.86) | 1 | 0.78 (0.02 - 85.58) |
| Turbo | 3 | 0.06 (0.01 - 7.44) | 4 | 5.84 (1.10 - 17.18) | 2 | 1.82 (0.11 - 9.87) |
| Turmequé | 0 | 0.00 (0.00 - 0.00) | 0 | 0.00 (0.00 - 0.00) | 1 | 7.38 (0.19 - 228.24) |
| Uramita | 1 | 2.45 (0.06 - 217.74) | 0 | 0.00 (0.00 - 0.00) | 0 | 0.00 (0.00 - 0.00) |
| Uribia | 2 | 1.07 (0.12 - 10.3) | 6 | 2.43 (0.46 - 30.12) | 1 | 0.72 (0.02 - 6.94) |
| Urrao | 0 | 0.00 (0.00 - 0.00) | 1 | 1.44 (0.04 - 38) | 0 | 0.00 (0.00 - 0.00) |
| Valencia | 2 | 8.50 (0.50 - 42.92) | 1 | 1.65 (0.04 - 25.55) | 0 | 0.00 (0.00 - 0.00) |
| Valle de San José | 1 | 22.06 (0.56 - 362.28) | 0 | 0.00 (0.00 - 0.00) | 0 | 0.00 (0.00 - 0.00) |
| Valle de San Juan | 1 | 17.30 (0.44 - 301.51) | 0 | 0.00 (0.00 - 0.00) | 0 | 0.00 (0.00 - 0.00) |
| Valle del Guamuez | 2 | 4.98 (0.18 - 31.89) | 1 | 9.15 (0.23 - 42.26) | 0 | 0.00 (0.00 - 0.00) |
| Valledupar | 43 | 24.84 (16.00 - 36.16) | 7 | 2.66 (0.42 - 7.74) | 27 | 11.30 (7.05 - 17.11) |
| Valparaíso | 1 | 4.20 (0.11 - 98.49) | 2 | 174.87 (5.42 - 651.09) | 0 | 0.00 (0.00 - 0.00) |
| Valparaíso | 1 | 17.49 (0.44 - 189.86) | 0 | 0.00 (0.00 - 0.00) | 0 | 0.00 (0.00 - 0.00) |
| Vélez | 0 | 0.00 (0.00 - 0.00) | 0 | 0.00 (0.00 - 0.00) | 1 | 15.18 (0.38 - 81.46) |
| Venadillo | 1 | 2.52 (0.06 - 49.69) | 0 | 0.00 (0.00 - 0.00) | 0 | 0.00 (0.00 - 0.00) |
| Venecia | 1 | 21.59 (0.55 - 113.58) | 1 | 5.78 (0.15 - 86.55) | 1 | 7.47 (0.19 - 81.22) |
| Ventaquemada | 1 | 1.99 (0.05 - 106.33) | 0 | 0.00 (0.00 - 0.00) | 1 | 4.87 (0.12 - 69.22) |
| Villa Caro | 1 | 12.14 (0.31 - 185.71) | 0 | 0.00 (0.00 - 0.00) | 0 | 0.00 (0.00 - 0.00) |
| Villa de Leyva | 0 | 0.00 (0.00 - 0.00) | 1 | 0.98 (0.02 - 59.51) | 1 | 19.16 (0.49 - 93.52) |
| Villa de San Diego de Ubaté | 1 | 10.46 (0.26 - 50.15) | 1 | 0.38 (0.01 - 36.17) | 1 | 0.03 (0.00 - 37.93) |
| Villa del Rosario | 9 | 20.58 (7.13 - 43.65) | 1 | 0.15 (0.00 - 48.14) | 1 | 0.94 (0.02 - 13.30) |
| Villa Rica | 2 | 16.65 (1.32 - 104.73) | 0 | 0.00 (0.00 - 0.00) | 3 | 51.66 (10.45 - 165.83) |
| Villagarzón | 1 | 4.79 (0.12 - 51.83) | 1 | 9.72 (0.25 - 69.66) | 0 | 0.00 (0.00 - 0.00) |
| Villahermosa | 0 | 0.00 (0.00 - 0.00) | 1 | 13.32 (0.34 - 119.16) | 0 | 0.00 (0.00 - 0.00) |
| Villamaría | 0 | 0.00 (0.00 - 0.00) | 2 | 8.83 (1.01 - 31.28) | 3 | 8.94 (0.59 - 34.10) |
| Villanueva | 3 | 75.14 (2.30 - 298.70) | 3 | 116.56 (14.46 - 335.99) | 2 | 23.78 (2.80 - 98.86) |
| Villanueva | 1 | 1.77 (0.04 - 28.95) | 0 | 0.00 (0.00 - 0.00) | 2 | 16.35 (1.83 - 124.31) |
| Villavicencio | 26 | 5.43 (1.97 - 11.15) | 10 | 2.79 (0.70 - 15.47) | 21 | 18.73 (8.99 - 32.55) |
| Villavieja | 0 | 0.00 (0.00 - 0.00) | 0 | 0.00 (0.00 - 0.00) | 1 | 5.45 (0.14 - 160.76) |
| Villeta | 1 | 5.23 (0.13 - 50.88) | 0 | 0.00 (0.00 - 0.00) | 0 | 0.00 (0.00 - 0.00) |
| Viracachá | 0 | 0.00 (0.00 - 0.00) | 0 | 0.00 (0.00 - 0.00) | 1 | 23.79 (0.60 - 275.92) |
| Vistahermosa | 1 | 8.38 (0.21 - 51.58) | 0 | 0.00 (0.00 - 0.00) | 0 | 0.00 (0.00 - 0.00) |
| Viterbo | 3 | 38.10 (4.58 - 130.46) | 0 | 0.00 (0.00 - 0.00) | 0 | 0.00 (0.00 - 0.00) |
| Yacuanquer | 1 | 138.79 (3.51 - 541.11) | 0 | 0.00 (0.00 - 0.00) | 0 | 0.00 (0.00 - 0.00) |
| Yaguará | 2 | 3.68 (0.16 - 247.66) | 0 | 0.00 (0.00 - 0.00) | 1 | 6.59 (0.17 - 108.01) |
| Yalí | 0 | 0.00 (0.00 - 0.00) | 1 | 12.84 (0.33 - 136.00) | 0 | 0.00 (0.00 - 0.00) |
| Yarumal | 4 | 9.29 (2.29 - 34.88) | 1 | 1.45 (0.04 - 19.36) | 0 | 0.00 (0.00 - 0.00) |
| Yolombó | 0 | 0.00 (0.00 - 0.00) | 1 | 1.22 (0.03 - 43.30) | 0 | 0.00 (0.00 - 0.00) |
| Yopal | 11 | 19.69 (8.22 - 37.78) | 10 | 17.81 (3.38 - 46.18) | 4 | 0.34 (0.06 - 10.35) |
| Yotoco | 3 | 9.27 (1.75 - 91.34) | 0 | 0.00 (0.00 - 0.00) | 0 | 0.00 (0.00 - 0.00) |
| Yumbo | 8 | 10.27 (3.78 - 22.44) | 2 | 0.47 (0.02 - 9.39) | 5 | 15.80 (3.20 - 40.84) |
| Zambrano | 0 | 0.00 (0.00 - 0.00) | 0 | 0.00 (0.00 - 0.00) | 2 | 7.87 (0.87 - 220.89) |
| Zaragoza | 1 | 3.31 (0.08 - 267.7) | 2 | 24.85 (1.59 - 95.34) | 1 | 3.70 (0.09 - 39.48) |
| Zarzal | 1 | 0.23 (0.01 - 28.76) | 1 | 0.34 (0.01 - 24.23) | 0 | 0.00 (0.00 - 0.00) |
| Zipaquirá | 15 | 15.73 (7.48 - 29.77) | 2 | 0.94 (0.05 - 14.51) | 3 | 2.98 (0.14 - 13.68) |
| Zona Bananera | 1 | 0.65 (0.02 - 53.78) | 3 | 16.65 (2.17 - 63.6) | 1 | 3.55 (0.09 - 30.60) |
| **National** | **4.506** | **18.69 (18.15 - 19.25)** | **1.425** | **5.93 (5.62 - 6.25)** | **2.593** | **11.34 (10.9 - 11.78)** |

^1^ All rates are age-standardized per 100.000 population. They were estimated including only invasive cases. The municipalities in which there were no cases for any of this types of cancer were excluded.

**Table S2. Mortality rates for breast. prostate and cervical cancer by municipalities. Colombia 2018^1^**

| **Municipality** | **Breast cancer** | | **Cervical cancer** | | **Prostate cancer** | |
| --- | --- | --- | --- | --- | --- | --- |
|  | **n** | **ASR (95% CI)** | **n** | **ASR (95% CI)** | **n** | **ASR (95% CI)** |
| Abejorral | 0 | 0.00 (0.00 - 0.00) | 1 | 4.50 (0.11 - 69.63) | 0 | 0.00 (0.00 - 0.00) |
| Acacías | 0 | 0.00 (0.00 - 0.00) | 0 | 0.00 (0.00 - 0.00) | 1 | 0.59 (0.02 - 11.29) |
| Acevedo | 0 | 0.00 (0.00 - 0.00) | 0 | 0.00 (0.00 - 0.00) | 1 | 5.14 (0.13 - 33.44) |
| Achí | 0 | 0.00 (0.00 - 0.00) | 0 | 0.00 (0.00 - 0.00) | 1 | 0.59 (0.01 - 39.12) |
| Agrado | 2 | 6.06 (0.73 - 205.84) | 0 | 0.00 (0.00 - 0.00) | 0 | 0.00 (0.00 - 0.00) |
| Agua de Dios | 13 | 191.63 (48.94 - 458.07) | 3 | 25.15 (2.21 - 116.55) | 6 | 31.58 (2.75 - 190.72) |
| Aguachica | 4 | 1.47 (0.29 - 11.69) | 1 | 0.53 (0.01 - 12.14) | 0 | 0.00 (0.00 - 0.00) |
| Aguazul | 2 | 2.51 (0.29 - 27.13) | 0 | 0.00 (0.00 - 0.00) | 0 | 0.00 (0.00 - 0.00) |
| Agustín Codazzi | 2 | 3.10 (0.35 - 24.68) | 0 | 0.00 (0.00 - 0.00) | 1 | 49.73 (1.26 - 191.65) |
| Aipe | 4 | 7.07 (1.77 - 33.45) | 1 | 6.85 (0.17 - 47.57) | 0 | 0.00 (0.00 - 0.00) |
| Alcalá | 0 | 0.00 (0.00 - 0.00) | 1 | 1.95 (0.05 - 43.91) | 0 | 0.00 (0.00 - 0.00) |
| Algarrobo | 0 | 0.00 (0.00 - 0.00) | 0 | 0.00 (0.00 - 0.00) | 1 | 6.46 (0.16 - 173.43) |
| Algeciras | 0 | 0.00 (0.00 - 0.00) | 5 | 70.48 (7.27 - 215.39) | 1 | 14.21 (0.36 - 71.28) |
| Altos del Rosario | 0 | 0.00 (0.00 - 0.00) | 1 | 3.67 (0.09 - 94.40) | 0 | 0.00 (0.00 - 0.00) |
| Amagá | 0 | 0.00 (0.00 - 0.00) | 1 | 28.37 (0.72 - 142.40) | 2 | 4.56 (0.54 - 37.28) |
| Ambalema | 0 | 0.00 (0.00 - 0.00) | 0 | 0.00 (0.00 - 0.00) | 1 | 8.41 (0.21 - 145.15) |
| Anapoima | 1 | 9.30 (0.24 - 136.12) | 0 | 0.00 (0.00 - 0.00) | 0 | 0.00 (0.00 - 0.00) |
| Andalucía | 1 | 12.03 (0.30 - 71.16) | 0 | 0.00 (0.00 - 0.00) | 1 | 3.41 (0.09 - 79.96) |
| Andes | 2 | 2.78 (0.31 - 38.75) | 0 | 0.00 (0.00 - 0.00) | 1 | 0.51 (0.01 - 16.77) |
| Angelópolis | 1 | 8.26 (0.21 - 127.78) | 0 | 0.00 (0.00 - 0.00) | 0 | 0.00 (0.00 - 0.00) |
| Anolaima | 0 | 0.00 (0.00 - 0.00) | 0 | 0.00 (0.00 - 0.00) | 2 | 2.37 (0.27 - 121.73) |
| Anorí | 0 | 0.00 (0.00 - 0.00) | 1 | 2.22 (0.06 - 58.90) | 0 | 0.00 (0.00 - 0.00) |
| Anserma | 1 | 1.23 (0.03 - 59.95) | 1 | 44.14 (1.12 - 170.63) | 1 | 0.72 (0.02 - 32.94) |
| Anza | 0 | 0.00 (0.00 - 0.00) | 0 | 0.00 (0.00 - 0.00) | 1 | 10.17 (0.26 - 105.66) |
| Apartadó | 4 | 35.01 (1.71 - 121.68) | 2 | 1.63 (0.14 - 10.13) | 6 | 1.75 (0.59 - 7.71) |
| Apulo | 0 | 0.00 (0.00 - 0.00) | 0 | 0.00 (0.00 - 0.00) | 2 | 19.69 (1.80 - 139.37) |
| Aracataca | 1 | 0.29 (0.01 - 32.92) | 2 | 8.10 (0.95 - 35.30) | 0 | 0.00 (0.00 - 0.00) |
| Aranzazu | 2 | 12.13 (0.84 - 104.56) | 0 | 0.00 (0.00 - 0.00) | 0 | 0.00 (0.00 - 0.00) |
| Aratoca | 3 | 39.86 (6.02 - 192.99) | 0 | 0.00 (0.00 - 0.00) | 0 | 0.00 (0.00 - 0.00) |
| Arauca (Arauca) | 1 | 2.79 (0.07 - 14.25) | 0 | 0.00 (0.00 - 0.00) | 2 | 2.96 (0.23 - 19.53) |
| Arauquita | 0 | 0.00 (0.00 - 0.00) | 1 | 4.20 (0.11 - 37.15) | 0 | 0.00 (0.00 - 0.00) |
| Arboletes | 1 | 7.85 (0.20- 49.41) | 1 | 4.40 (0.11 - 52.59) | 0 | 0.00 (0.00 - 0.00) |
| Arcabuco | 1 | 24.06 (0.61 - 232.87) | 0 | 0.00 (0.00 - 0.00) | 0 | 0.00 (0.00 - 0.00) |
| Argelia (Cauca) | 0 | 0.00 (0.00 - 0.00) | 1 | 4.64 (0.12 - 64.35) | 0 | 0.00 (0.00 - 0.00) |
| Argelia (Valle del Cauca) | 0 | 0.00 (0.00 - 0.00) | 1 | 10.49 (0.27 - 169.52) | 0 | 0.00 (0.00 - 0.00) |
| Ariguaní | 1 | 0.92 (0.02 - 29.98) | 0 | 0.00 (0.00 - 0.00) | 0 | 0.00 (0.00 - 0.00) |
| Arjona | 9 | 13.29 (2.27 - 40.79) | 1 | 0.13 (0.00 - 18.25) | 4 | 20.85 (3.88 – 56.00) |
| Armenia (Antioquia) | 7 | 83.19 (24.61 - 383.65) | 2 | 331.69 (14.09 - 1200.34) | 4 | 123.15 (28.18 - 380.55) |
| Armenia (Quindío) | 19 | 10.04 (5.4 - 17.04) | 8 | 1.21 (0.38 - 4.73) | 15 | 3.02 (1.66 - 6.27) |
| Ayapel | 0 | 0.00 (0.00 - 0.00) | 1 | 4.22 (0.11 - 33.53) | 3 | 58.04 (4.21 - 189.76) |
| Balboa (Cauca) | 0 | 0.00 (0.00 - 0.00) | 1 | 10.16 (0.26 - 71.64) | 0 | 0.00 (0.00 - 0.00) |
| Baranoa | 1 | 0.36 (0.01 - 18.23) | 1 | 1.07 (0.03 - 17.72) | 0 | 0.00 (0.00 - 0.00) |
| Barbosa (Antioquia) | 3 | 3.24 (0.46 - 19.64) | 0 | 0.00 (0.00 - 0.00) | 1 | 1.92 (0.05 - 21.00) |
| Barrancabermeja | 14 | 12.43 (6.64 - 21.46) | 2 | 6.89 (0.21 - 26.17) | 10 | 7.72 (3.67 - 14.83) |
| Barrancas | 2 | 2.28 (0.24 - 51.67) | 1 | 0.16 (0.00 - 34.11) | 0 | 0.00 (0.00 - 0.00) |
| Barranco de Loba | 1 | 1.33 (0.03 - 69.13) | 1 | 6.70 (0.17 - 80.41) | 0 | 0.00 (0.00 - 0.00) |
| Barranquilla | 104 | 24.35 (18.10 - 31.71) | 46 | 9.90 (5.47 - 15.81) | 78 | 13.76 (10.71 - 17.38) |
| Becerril | 0 | 0.00 (0.00 - 0.00) | 1 | 2.40 (0.06 - 406.39) | 1 | 11.35 (0.29 - 133.04) |
| Belalcázar | 1 | 10.08 (0.26 - 129.77) | 2 | 94.62 (9.25 - 300.1) | 0 | 0.00 (0.00 - 0.00) |
| Belén de Los Andaquies | 1 | 54.06 (1.37 - 266.17) | 0 | 0.00 (0.00 - 0.00) | 0 | 0.00 (0.00 - 0.00) |
| Belén de Umbría | 0 | 0.00 (0.00 - 0.00) | 0 | 0.00 (0.00 - 0.00) | 1 | 0.72 (0.02 - 33.99) |
| Belén (Boyacá) | 1 | 14.39 (0.36 - 233.27) | 0 | 0.00 (0.00 - 0.00) | 0 | 0.00 (0.00 - 0.00) |
| Belén (Nariño) | 0 | 0.00 (0.00 - 0.00) | 1 | 2.85 (0.07 - 114.72) | 0 | 0.00 (0.00 - 0.00) |
| Bello | 32 | 15.98 (10.22 - 23.52) | 10 | 8.03 (1.86 - 19.14) | 13 | 9.94 (5.06 - 17.09) |
| Boavita | 0 | 0.00 (0.00 - 0.00) | 0 | 0.00 (0.00 - 0.00) | 1 | 6.58 (0.17 - 214.45) |
| Bogotá D.C. | 442 | 27.03 (20.86 - 34.04) | 160 | 2.47 (1.62 - 3.53) | 292 | 3.69 (3.16 - 4.28) |
| Bojaya | 0 | 0.00 (0.00 - 0.00) | 1 | 4.48 (0.11 - 336.60) | 0 | 0.00 (0.00 - 0.00) |
| Bolívar (Cauca) | 1 | 0.30 (0.01 - 39.12) | 0 | 0.00 (0.00 - 0.00) | 1 | 1.62 (0.04 - 50.44) |
| Bolívar (Santander) | 0 | 0.00 (0.00 - 0.00) | 0 | 0.00 (0.00 - 0.00) | 1 | 17.10 (0.43 - 106.85) |
| Bolívar (Valle del Cauca) | 2 | 31.96 (2.10 - 127.72) | 0 | 0.00 (0.00 - 0.00) | 0 | 0.00 (0.00 - 0.00) |
| Bosconia | 1 | 5.94 (0.15 - 41.42) | 1 | 3.58 (0.09 - 97.67) | 1 | 0.17 (0.00 - 52.76) |
| Briceño (Boyacá) | 0 | 0.00 (0.00 - 0.00) | 0 | 0.00 (0.00 - 0.00) | 1 | 20.24 (0.51 - 383.61) |
| Bucaramanga | 52 | 11.96 (8.21 - 16.83) | 12 | 1.92 (0.56 - 4.76) | 36 | 19.78 (12.63 - 29.04) |
| Buenaventura | 11 | 4.42 (1.40 - 9.8) | 7 | 2.01 (0.59 - 5.08) | 6 | 4.21 (1.29 - 9.76) |
| Buenos Aires | 0 | 0.00 (0.00 - 0.00) | 0 | 0.00 (0.00 - 0.00) | 1 | 17.35 (0.44 - 72.53) |
| Buesaco | 2 | 53.73 (6.50 - 168.16) | 1 | 97.74 (2.47 - 376.25) | 1 | 8.06 (0.20 - 44.61) |
| Bugalagrande | 2 | 6.37 (0.73 - 45.86) | 0 | 0.00 (0.00 - 0.00) | 0 | 0.00 (0.00 - 0.00) |
| Cáceres | 1 | 4.40 (0.11 - 28.71) | 3 | 5.58 (1.12 - 28.96) | 0 | 0.00 (0.00 - 0.00) |
| Caicedonia | 6 | 160.53 (6.24 - 577.14) | 1 | 2.92 (0.07 - 35.39) | 1 | 0.35 (0.01 - 48.27) |
| Caimito | 1 | 5.57 (0.14 – 255.00) | 0 | 0.00 (0.00 - 0.00) | 0 | 0.00 (0.00 - 0.00) |
| Cajamarca | 1 | 24.60 (0.62 - 121.09) | 0 | 0.00 (0.00 - 0.00) | 1 | 3.82 (0.10 - 106.85) |
| Cajibío | 0 | 0.00 (0.00 - 0.00) | 2 | 3.99 (0.45 - 31.08) | 0 | 0.00 (0.00 - 0.00) |
| Cajicá | 1 | 0.54 (0.01 - 26.08) | 0 | 0.00 (0.00 - 0.00) | 1 | 7.53 (0.19 - 33.76) |
| Calamar (Bolívar) | 1 | 4.55 (0.12 - 71.34) | 1 | 1.38 (0.03 - 34.93) | 0 | 0.00 (0.00 - 0.00) |
| Calamar (Guavaire) | 0 | 0.00 (0.00 - 0.00) | 1 | 22.31 (0.56 - 171.92) | 0 | 0.00 (0.00 - 0.00) |
| Calarcá | 3 | 6.95 (1.35 - 21.65) | 2 | 2.86 (0.10- 17.79) | 7 | 1.27 (0.51 - 26.87) |
| Caldas (Antioquia) | 6 | 8.72 (2.38 - 26.3) | 1 | 0.82 (0.02 - 13.1) | 7 | 53.89 (14.06 - 123.6) |
| Caldono | 0 | 0.00 (0.00 - 0.00) | 3 | 18.42 (3.08 - 60.21) | 1 | 2.13 (0.05 - 74.31) |
| Cali | 242 | 9.86 (8.34 - 11.59) | 53 | 3.45 (1.88 - 5.58) | 158 | 7.55 (6.24 - 9.12) |
| Calima | 1 | 2.58 (0.07 - 57.42) | 0 | 0.00 (0.00 - 0.00) | 0 | 0.00 (0.00 - 0.00) |
| Caloto | 0 | 0.00 (0.00 - 0.00) | 1 | 1.46 (0.04 - 92.62) | 1 | 10.08 (0.26 - 76.69) |
| Campo de La Cruz | 0 | 0.00 (0.00 - 0.00) | 1 | 5.37 (0.14 - 72.47) | 2 | 11.79 (0.59 - 83.51) |
| Campoalegre | 2 | 1.81 (0.22 - 47.21) | 0 | 0.00 (0.00 - 0.00) | 1 | 6.49 (0.16 - 68.09) |
| Canalete | 0 | 0.00 (0.00 - 0.00) | 2 | 46.61 (2.40 - 175.93) | 0 | 0.00 (0.00 - 0.00) |
| Candelaria (Atlántico) | 4 | 8.84 (1.77 - 143.38) | 2 | 116.47 (5.56 - 418.92) | 0 | 0.00 (0.00 - 0.00) |
| Candelaria (Valle del Cauca) | 4 | 4.88 (0.47 - 22.73) | 0 | 0.00 (0.00 - 0.00) | 1 | 0.53 (0.01 - 11.62) |
| Cantagallo | 0 | 0.00 (0.00 - 0.00) | 1 | 639.65 (16.19 - 2443.75) | 0 | 0.00 (0.00 - 0.00) |
| Caparrapí | 1 | 17.97 (0.45 - 106.94) | 0 | 0.00 (0.00 - 0.00) | 0 | 0.00 (0.00 - 0.00) |
| Caqueza | 1 | 1.21 (0.03 - 64.23) | 0 | 0.00 (0.00 - 0.00) | 0 | 0.00 (0.00 - 0.00) |
| Caramanta | 1 | 19.14 (0.48 - 192.46) | 0 | 0.00 (0.00 - 0.00) | 0 | 0.00 (0.00 - 0.00) |
| Carepa | 1 | 12.54 (0.32 - 50.89) | 1 | 2.99 (0.08 - 32.29) | 0 | 0.00 (0.00 - 0.00) |
| Cartagena | 76 | 3.56 (2.61 - 5.18) | 37 | 5.44 (2.93 - 8.90) | 56 | 12.55 (9.16 - 16.71) |
| Cartagena del Chairá | 0 | 0.00 (0.00 - 0.00) | 1 | 2.87 (0.07 - 78.47) | 1 | 2.42 (0.06 - 34.47) |
| Cartago | 11 | 24.03 (9.79 - 46.31) | 0 | 0.00 (0.00 - 0.00) | 7 | 8.46 (2.39 - 20.51) |
| Caucasia | 4 | 7.73 (0.48 - 28.72) | 4 | 10.15 (1.10 - 31.54) | 1 | 0.10 (0.00 - 43.97) |
| Cereté | 3 | 4.44 (0.82 - 15.76) | 3 | 0.88 (0.04 - 9.6) | 5 | 5.14 (1.65 - 16.08) |
| Chaguaní | 1 | 32.75 (0.83 - 278.30) | 0 | 0.00 (0.00 - 0.00) | 0 | 0.00 (0.00 - 0.00) |
| Chalán | 0 | 0.00 (0.00 - 0.00) | 0 | 0.00 (0.00 - 0.00) | 1 | 9.98 (0.25 - 230.11) |
| Chaparral | 1 | 1.19 (0.03 - 39.59) | 1 | 1.06 (0.03 - 27.01) | 1 | 8.92 (0.23 - 45.39) |
| Charalá | 1 | 13.96 (0.35 - 256.06) | 0 | 0.00 (0.00 - 0.00) | 0 | 0.00 (0.00 - 0.00) |
| Charta | 1 | 108.46 (2.75 - 561.07) | 0 | 0.00 (0.00 - 0.00) | 0 | 0.00 (0.00 - 0.00) |
| Chía | 8 | 6.49 (2.32 - 18.02) | 0 | 0.00 (0.00 - 0.00) | 7 | 2.10 (0.71 - 13.31) |
| Chigorodó | 2 | 16.86 (1.48 - 57.09) | 6 | 31.27 (11.42 - 64.93) | 1 | 0.99 (0.03 - 40.03) |
| Chimichagua | 0 | 0.00 (0.00 - 0.00) | 0 | 0.00 (0.00 - 0.00) | 1 | 3.13 (0.08 - 36.72) |
| Chinácota | 0 | 0.00 (0.00 - 0.00) | 1 | 2.78 (0.07 - 59.85) | 0 | 0.00 (0.00 - 0.00) |
| Chinchiná | 6 | 19.27 (5.74 - 46.41) | 1 | 0.38 (0.01 - 17.68) | 0 | 0.00 (0.00 - 0.00) |
| Chinú | 4 | 8.37 (1.45 - 30.21) | 0 | 0.00 (0.00 - 0.00) | 0 | 0.00 (0.00 - 0.00) |
| Chiquinquirá | 1 | 1.90 (0.05 - 21.26) | 2 | 24.54 (1.08 - 87.18) | 5 | 122.45 (3.52 - 459.41) |
| Chiriguaná | 1 | 18.12 (0.46 - 98.95) | 0 | 0.00 (0.00 - 0.00) | 0 | 0.00 (0.00 - 0.00) |
| Chivolo | 1 | 6.23 (0.16 - 142.61) | 0 | 0.00 (0.00 - 0.00) | 1 | 3.38 (0.09 - 54.69) |
| Choachí | 1 | 7.01 (0.18 - 142.74) | 0 | 0.00 (0.00 - 0.00) | 1 | 26.05 (0.66 - 207.86) |
| Chocontá | 0 | 0.00 (0.00 - 0.00) | 0 | 0.00 (0.00 - 0.00) | 2 | 1.45 (0.18 - 54.76) |
| Ciénaga de Oro | 3 | 1.58 (0.30 - 17.47) | 0 | 0.00 (0.00 - 0.00) | 1 | 5.19 (0.13 - 25.70) |
| Ciénaga (Magdalena) | 4 | 3.3 (0.83 - 13.93) | 4 | 13.48 (2.46 - 36.22) | 4 | 3.61 (0.9 - 13.58) |
| Circasia | 0 | 0.00 (0.00 - 0.00) | 0 | 0.00 (0.00 - 0.00) | 1 | 0.26 (0.01 - 57.06) |
| Clemencia | 0 | 0.00 (0.00 - 0.00) | 1 | 2.68 (0.07 - 92.20) | 0 | 0.00 (0.00 - 0.00) |
| Coello | 0 | 0.00 (0.00 - 0.00) | 1 | 8.98 (0.23 - 102.98) | 0 | 0.00 (0.00 - 0.00) |
| Cogua | 0 | 0.00 (0.00 - 0.00) | 0 | 0.00 (0.00 - 0.00) | 1 | 15.75 (0.40 - 104.25) |
| Colón (Nariño) | 0 | 0.00 (0.00 - 0.00) | 0 | 0.00 (0.00 - 0.00) | 1 | 33.99 (0.86 - 260.91) |
| Colón (Putumayo) | 0 | 0.00 (0.00 - 0.00) | 1 | 280.51 (7.10 - 1078.45) | 0 | 0.00 (0.00 - 0.00) |
| Coloso | 0 | 0.00 (0.00 - 0.00) | 0 | 0.00 (0.00 - 0.00) | 1 | 33.42 (0.85 - 223.65) |
| Cómbita | 1 | 10.26 (0.26 - 115.36) | 1 | 73.5 (1.86 - 296.65) | 0 | 0.00 (0.00 - 0.00) |
| Concepción (Santander) | 0 | 0.00 (0.00 - 0.00) | 0 | 0.00 (0.00 - 0.00) | 1 | 5.43 (0.14 - 155.81) |
| Concordia (Antioquia) | 1 | 23.92 (0.61 – 109.00) | 0 | 0.00 (0.00 - 0.00) | 0 | 0.00 (0.00 - 0.00) |
| Concordia (Magdalena) | 0 | 0.00 (0.00 - 0.00) | 1 | 46.47 (1.18 - 216.60) | 0 | 0.00 (0.00 - 0.00) |
| Consaca | 0 | 0.00 (0.00 - 0.00) | 1 | 2.01 (0.05 - 95.02) | 0 | 0.00 (0.00 - 0.00) |
| Contadero | 0 | 0.00 (0.00 - 0.00) | 0 | 0.00 (0.00 - 0.00) | 1 | 0.88 (0.02 - 206.33) |
| Copacabana | 7 | 22.17 (3.83 - 60.39) | 1 | 0.69 (0.02 - 16.56) | 2 | 3.68 (0.26 - 17.37) |
| Córdoba (Bolívar) | 0 | 0.00 (0.00 - 0.00) | 1 | 20.37 (0.52 - 255.93) | 2 | 22.54 (2.73 - 114.25) |
| Corinto | 1 | 2.94 (0.07 - 30.69) | 2 | 42.28 (1.67 - 156.32) | 1 | 14.75 (0.37 - 67.79) |
| Corozal | 3 | 4.35 (0.76 - 18.52) | 3 | 27.73 (0.94 - 104.05) | 2 | 1.88 (0.23 - 20.29) |
| Cota | 0 | 0.00 (0.00 - 0.00) | 1 | 2.66 (0.07 - 38.04) | 0 | 0.00 (0.00 - 0.00) |
| Cotorra | 0 | 0.00 (0.00 - 0.00) | 0 | 0.00 (0.00 - 0.00) | 1 | 3.29 (0.08 - 61.05) |
| Covarachía | 1 | 2.74 (0.07 - 500.57) | 0 | 0.00 (0.00 - 0.00) | 0 | 0.00 (0.00 - 0.00) |
| Coveñas | 0 | 0.00 (0.00 - 0.00) | 0 | 0.00 (0.00 - 0.00) | 1 | 15.51 (0.39 - 82.63) |
| Coyaima | 0 | 0.00 (0.00 - 0.00) | 0 | 0.00 (0.00 - 0.00) | 1 | 23.31 (0.59 - 100.55) |
| Cúcuta | 39 | 11.89 (7.05 - 18.35) | 27 | 1.05 (0.65 - 2.71) | 30 | 11.71 (7.45 - 17.32) |
| Cumbal | 1 | 3.78 (0.10 - 25.71) | 1 | 1.73 (0.04 - 40.14) | 2 | 3.01 (0.12 - 41.08) |
| Cunday | 1 | 44.13 (1.12 - 237.11) | 1 | 5.43 (0.14 - 139.11) | 0 | 0.00 (0.00 - 0.00) |
| Curillo | 1 | 17.85 (0.45 - 102.23) | 0 | 0.00 (0.00 - 0.00) | 0 | 0.00 (0.00 - 0.00) |
| Curumaní | 1 | 2.82 (0.07 - 53.21) | 0 | 0.00 (0.00 - 0.00) | 0 | 0.00 (0.00 - 0.00) |
| Dagua | 0 | 0.00 (0.00 - 0.00) | 2 | 6.47 (0.25 - 53.40) | 0 | 0.00 (0.00 - 0.00) |
| Dibulla | 0 | 0.00 (0.00 - 0.00) | 0 | 0.00 (0.00 - 0.00) | 1 | 9.55 (0.24 - 70.58) |
| Distracción | 0 | 0.00 (0.00 - 0.00) | 0 | 0.00 (0.00 - 0.00) | 2 | 48.34 (5.85 - 154.11) |
| Don Matías | 1 | 14.87 (0.38 - 72.25) | 0 | 0.00 (0.00 - 0.00) | 0 | 0.00 (0.00 - 0.00) |
| Dosquebradas | 15 | 15.20 (3.45 - 36.92) | 4 | 17.11 (0.51 - 64.13) | 11 | 8.67 (3.75 - 17.01) |
| Duitama | 5 | 5.30 (1.20 - 15.84) | 1 | 6.73 (0.17 - 27.27) | 2 | 1.50 (0.18 - 22.75) |
| Ebéjico | 1 | 5.87 (0.15 - 137.94) | 0 | 0.00 (0.00 - 0.00) | 4 | 4.99 (1.30 - 98.04) |
| El Bagre | 0 | 0.00 (0.00 - 0.00) | 1 | 3.09 (0.08 - 25.94) | 0 | 0.00 (0.00 - 0.00) |
| El Banco | 1 | 1.41 (0.04 - 22.45) | 3 | 28.73 (5.18 - 77.26) | 0 | 0.00 (0.00 - 0.00) |
| El Cantón del San Pablo | 0 | 0.00 (0.00 - 0.00) | 1 | 15.07 (0.38 - 124.87) | 0 | 0.00 (0.00 - 0.00) |
| El Carmen de Bolívar | 3 | 3.70 (0.76 - 39.19) | 2 | 1.53 (0.05 - 33.96) | 3 | 12.16 (2.51 - 39.61) |
| El Carmen de Chucurí | 0 | 0.00 (0.00 - 0.00) | 1 | 6.25 (0.16 - 70.25) | 0 | 0.00 (0.00 - 0.00) |
| El Carmen de Viboral | 0 | 0.00 (0.00 - 0.00) | 1 | 3.55 (0.09 - 28.37) | 1 | 5.84 (0.15 - 31.94) |
| El Cerrito | 6 | 26.26 (7.50 - 61.38) | 0 | 0.00 (0.00 - 0.00) | 2 | 2.25 (0.24 - 17.34) |
| El Cocuy | 0 | 0.00 (0.00 - 0.00) | 2 | 16.30 (1.94 - 240.97) | 0 | 0.00 (0.00 - 0.00) |
| El Colegio | 1 | 14.66 (0.37 - 79.27) | 0 | 0.00 (0.00 - 0.00) | 1 | 11.48 (0.29 - 62.05) |
| El Copey | 0 | 0.00 (0.00 - 0.00) | 0 | 0.00 (0.00 - 0.00) | 1 | 46.12 (1.17 - 181.13) |
| El Doncello | 1 | 2.02 (0.05 - 53.17) | 0 | 0.00 (0.00 - 0.00) | 3 | 23.77 (1.87 - 87.89) |
| El Guamo | 1 | 8.86 (0.22 - 165.55) | 0 | 0.00 (0.00 - 0.00) | 0 | 0.00 (0.00 - 0.00) |
| El Litoral del San Juan | 0 | 0.00 (0.00 - 0.00) | 0 | 0.00 (0.00 - 0.00) | 1 | 2.24 (0.06 - 70.77) |
| El Paso | 1 | 3.19 (0.08 - 151.72) | 0 | 0.00 (0.00 - 0.00) | 0 | 0.00 (0.00 - 0.00) |
| El Peñol | 1 | 17.52 (0.44 - 175.86) | 3 | 2.086.38 (253.7 - 5.918.24) | 1 | 18.89 (0.48 - 225.83) |
| El Retén | 0 | 0.00 (0.00 - 0.00) | 1 | 23.93 (0.61 - 114.51) | 0 | 0.00 (0.00 - 0.00) |
| El Retorno | 0 | 0.00 (0.00 - 0.00) | 1 | 18.16 (0.46 - 153.73) | 0 | 0.00 (0.00 - 0.00) |
| El Roble | 0 | 0.00 (0.00 - 0.00) | 0 | 0.00 (0.00 - 0.00) | 1 | 3.24 (0.08 - 128.61) |
| El Santuario | 1 | 0.84 (0.02 - 70.3) | 0 | 0.00 (0.00 - 0.00) | 0 | 0.00 (0.00 - 0.00) |
| El Tablón de Gómez | 0 | 0.00 (0.00 - 0.00) | 0 | 0.00 (0.00 - 0.00) | 1 | 8.78 (0.22 - 77.78) |
| El Tambo (Cauca) | 0 | 0.00 (0.00 - 0.00) | 0 | 0.00 (0.00 - 0.00) | 3 | 6.15 (1.05 - 24.94) |
| El Tambo (Nariño) | 0 | 0.00 (0.00 - 0.00) | 0 | 0.00 (0.00 - 0.00) | 1 | 3.76 (0.1 - 113.12) |
| El Zulia | 1 | 0.31 (0.01 - 63.72) | 0 | 0.00 (0.00 - 0.00) | 0 | 0.00 (0.00 - 0.00) |
| Encino | 0 | 0.00 (0.00 - 0.00) | 0 | 0.00 (0.00 - 0.00) | 1 | 114.74 (2.91 - 577.11) |
| Entrerrios | 2 | 20.76 (2.51 - 131.72) | 0 | 0.00 (0.00 - 0.00) | 0 | 0.00 (0.00 - 0.00) |
| Envigado | 20 | 25.43 (4.99 - 63.48) | 3 | 1.58 (0.25 - 5.83) | 17 | 6.83 (3.53 - 12.58) |
| Espinal | 7 | 17.42 (4.01 - 44.93) | 0 | 0.00 (0.00 - 0.00) | 1 | 4.32 (0.11 - 21.71) |
| Facatativá | 2 | 3.46 (0.21 - 13.75) | 3 | 4.30 (0.54 - 14.98) | 3 | 7.1 (1.00 - 21.27) |
| Filandia | 0 | 0.00 (0.00 - 0.00) | 0 | 0.00 (0.00 - 0.00) | 3 | 16.42 (3.38 - 102.62) |
| Firavitoba | 1 | 1.51 (0.04 - 186.3) | 0 | 0.00 (0.00 - 0.00) | 0 | 0.00 (0.00 - 0.00) |
| Flandes | 1 | 20.95 (0.53 - 86.46) | 1 | 2.13 (0.05 - 30.73) | 0 | 0.00 (0.00 - 0.00) |
| Florencia (Caquetá) | 6 | 0.91 (0.31 - 6.70) | 9 | 4.64 (1.33 - 12.4) | 6 | 9.85 (2.48 - 23.89) |
| Florida | 4 | 7.22 (1.45 - 25.50) | 1 | 1.02 (0.03 - 32.52) | 3 | 5.49 (0.51 - 24.53) |
| Floridablanca | 21 | 7.52 (3.03 - 14.93) | 1 | 0.59 (0.01 - 4.58) | 9 | 3.07 (1.26 - 7.18) |
| Fonseca | 0 | 0.00 (0.00 - 0.00) | 1 | 2.93 (0.07 - 27.71) | 2 | 18.93 (1.01 - 74.45) |
| Fredonia | 1 | 1.99 (0.05 - 69.21) | 0 | 0.00 (0.00 - 0.00) | 2 | 16.46 (1.27 - 66.35) |
| Fresno | 0 | 0.00 (0.00 - 0.00) | 1 | 0.57 (0.01 - 51.19) | 0 | 0.00 (0.00 - 0.00) |
| Frontino | 1 | 9.93 (0.25 - 71.24) | 3 | 30.94 (5.33 - 183.03) | 1 | 1.81 (0.05 - 73.59) |
| Fuente de Oro | 1 | 10.11 (0.26 - 93.13) | 0 | 0.00 (0.00 - 0.00) | 0 | 0.00 (0.00 - 0.00) |
| Fundación | 19 | 23.22 (13.67 - 48.53) | 1 | 0.65 (0.02 - 18.41) | 1 | 1.05 (0.03 - 14.66) |
| Funes | 1 | 2.93 (0.07 - 237.41) | 1 | 1.69 (0.04 - 365.43) | 0 | 0.00 (0.00 - 0.00) |
| Funza | 1 | 2.00 (0.05 – 17.00) | 0 | 0.00 (0.00 - 0.00) | 1 | 0.1 (0.00 - 20.43) |
| Fúquene | 0 | 0.00 (0.00 - 0.00) | 0 | 0.00 (0.00 - 0.00) | 2 | 45.98 (5.25 - 259.39) |
| Fusagasugá | 3 | 1.02 (0.18 - 8.38) | 2 | 1.32 (0.14 - 7.30) | 5 | 7.36 (1.90 - 18.52) |
| Gachala | 1 | 11.16 (0.28 - 258.18) | 0 | 0.00 (0.00 - 0.00) | 0 | 0.00 (0.00 - 0.00) |
| Gachantivá | 0 | 0.00 (0.00 - 0.00) | 0 | 0.00 (0.00 - 0.00) | 1 | 146.68 (3.71 - 704.10) |
| Gachetá | 1 | 6.22 (0.16 - 96.43) | 0 | 0.00 (0.00 - 0.00) | 0 | 0.00 (0.00 - 0.00) |
| Galapa | 0 | 0.00 (0.00 - 0.00) | 1 | 5.98 (0.15 - 31.98) | 1 | 3.83 (0.10 - 44.53) |
| Galeras | 3 | 5.08 (1.04 - 46.67) | 1 | 8.90 (0.23 - 109.43) | 0 | 0.00 (0.00 - 0.00) |
| Gama | 0 | 0.00 (0.00 - 0.00) | 0 | 0.00 (0.00 - 0.00) | 1 | 34.17 (0.87 - 310.70) |
| Gamarra | 1 | 3.14 (0.08 - 63.16) | 0 | 0.00 (0.00 - 0.00) | 0 | 0.00 (0.00 - 0.00) |
| Gameza | 0 | 0.00 (0.00 - 0.00) | 1 | 94.36 (2.39 - 451.81) | 0 | 0.00 (0.00 - 0.00) |
| Garagoa | 1 | 5.37 (0.14 - 99.45) | 0 | 0.00 (0.00 - 0.00) | 2 | 24.93 (2.71 - 93.94) |
| Garzón | 4 | 13.51 (1.23 - 45.05) | 2 | 3.94 (0.15 - 20.11) | 2 | 0.51 (0.04 - 38.68) |
| Gigante | 1 | 1.48 (0.04 - 29.44) | 1 | 2.45 (0.06 - 62.01) | 0 | 0.00 (0.00 - 0.00) |
| Ginebra | 3 | 6.24 (0.99 - 52.82) | 1 | 4.31 (0.11 - 54.22) | 0 | 0.00 (0.00 - 0.00) |
| Girardot | 6 | 6.16 (0.67 - 23.26) | 1 | 0.03 (0.00 - 16.91) | 4 | 34.31 (4.45 - 97.15) |
| Girardota | 5 | 14.02 (1.76 - 45.93) | 0 | 0.00 (0.00 - 0.00) | 2 | 28.91 (1.15 - 106.38) |
| Girón | 8 | 10.27 (2.97 - 23.26) | 5 | 5.64 (0.60 - 18.09) | 5 | 3.06 (0.92 - 9.14) |
| Gómez Plata | 3 | 17.30 (3.18 - 81.40) | 0 | 0.00 (0.00 - 0.00) | 1 | 4.22 (0.11 - 82.50) |
| Granada (Meta) | 3 | 2.58 (0.31 - 21.45) | 2 | 2.14 (0.18 - 25.66) | 0 | 0.00 (0.00 - 0.00) |
| Guacarí | 0 | 0.00 (0.00 - 0.00) | 4 | 13.77 (2.70 - 43.93) | 1 | 1.44 (0.04 - 44.76) |
| Guachucal | 0 | 0.00 (0.00 - 0.00) | 0 | 0.00 (0.00 - 0.00) | 1 | 0.85 (0.02 - 86.82) |
| Guadalajara de Buga | 8 | 12.66 (2.64 - 33.36) | 3 | 4.91 (0.46 - 18.20) | 8 | 15.08 (4.70 - 33.98) |
| Guadalupe (Santander) | 0 | 0.00 (0.00 - 0.00) | 0 | 0.00 (0.00 - 0.00) | 1 | 10.06 (0.25 - 267.46) |
| Guaduas | 1 | 0.24 (0.01 - 99.25) | 2 | 3.36 (0.39 - 24.06) | 0 | 0.00 (0.00 - 0.00) |
| Guaitarilla | 0 | 0.00 (0.00 - 0.00) | 0 | 0.00 (0.00 - 0.00) | 1 | 13.64 (0.35 - 89.72) |
| Gualmatán | 1 | 12.43 (0.31 - 173.30) | 0 | 0.00 (0.00 - 0.00) | 1 | 6.68 (0.17 - 187.61) |
| Guamal (Magdalena) | 1 | 5.49 (0.14 - 42.35) | 0 | 0.00 (0.00 - 0.00) | 1 | 3.39 (0.09 - 41.72) |
| Guapi | 1 | 6.07 (0.15 - 44.92) | 0 | 0.00 (0.00 - 0.00) | 0 | 0.00 (0.00 - 0.00) |
| Guapotá | 0 | 0.00 (0.00 - 0.00) | 0 | 0.00 (0.00 - 0.00) | 1 | 85.55 (2.17 - 524.79) |
| Guaranda | 1 | 65.17 (1.65 - 267.08) | 0 | 0.00 (0.00 - 0.00) | 1 | 1.49 (0.04 - 41.79) |
| Guarne | 0 | 0.00 (0.00 - 0.00) | 1 | 0.93 (0.02 - 22.63) | 2 | 3.30 (0.31 - 22.70) |
| Guataquí | 1 | 5.66 (0.14 - 376.70) | 0 | 0.00 (0.00 - 0.00) | 1 | 134.81 (3.41 - 727.80) |
| Guática | 1 | 31.98 (0.81 - 143.54) | 0 | 0.00 (0.00 - 0.00) | 1 | 14.08 (0.36 - 92.15) |
| Guayatá | 0 | 0.00 (0.00 - 0.00) | 0 | 0.00 (0.00 - 0.00) | 1 | 14.13 (0.36 - 211.84) |
| Hatillo de Loba | 0 | 0.00 (0.00 - 0.00) | 0 | 0.00 (0.00 - 0.00) | 1 | 16.75 (0.42 - 105.41) |
| Heliconia | 1 | 21.46 (0.54 - 188.83) | 0 | 0.00 (0.00 - 0.00) | 0 | 0.00 (0.00 - 0.00) |
| Hispania | 1 | 60.86 (1.54 - 363.43) | 0 | 0.00 (0.00 - 0.00) | 0 | 0.00 (0.00 - 0.00) |
| Honda | 2 | 12.81 (1.40 - 69.99) | 0 | 0.00 (0.00 - 0.00) | 0 | 0.00 (0.00 - 0.00) |
| Ibagué | 29 | 7.34 (3.89 - 12.30) | 9 | 3.41 (0.80 - 8.35) | 18 | 2.56 (1.23 - 4.95) |
| Inzá | 0 | 0.00 (0.00 - 0.00) | 1 | 8.76 (0.22 - 46.60) | 0 | 0.00 (0.00 - 0.00) |
| Ipiales | 5 | 32.17 (4.58 – 88.00) | 3 | 10.92 (1.56 - 31.92) | 4 | 2.19 (0.59 - 16.23) |
| Iquira | 0 | 0.00 (0.00 - 0.00) | 0 | 0.00 (0.00 - 0.00) | 1 | 12.4 (0.31 - 86.21) |
| Isnos | 1 | 0.99 (0.02 - 49.36) | 1 | 1.33 (0.03 - 33.66) | 0 | 0.00 (0.00 - 0.00) |
| Istmina | 2 | 7.41 (0.7 - 49.09) | 1 | 6.06 (0.15 - 51.25) | 0 | 0.00 (0.00 - 0.00) |
| Itagui | 21 | 4.68 (2.60 - 9.80) | 7 | 8.93 (1.47 - 24.30) | 11 | 2.34 (0.74 - 6.77) |
| Ituango | 0 | 0.00 (0.00 - 0.00) | 0 | 0.00 (0.00 - 0.00) | 1 | 3.64 (0.09 - 73.64) |
| Jamundí | 3 | 1.59 (0.31 - 12.29) | 1 | 0.2 (0.01 - 9.58) | 2 | 0.93 (0.03 - 8.05) |
| Jericó (Antioquia) | 0 | 0.00 (0.00 - 0.00) | 0 | 0.00 (0.00 - 0.00) | 1 | 61.59 (1.56 - 256.79) |
| Jerusalén | 0 | 0.00 (0.00 - 0.00) | 1 | 154.93 (3.92 - 711.75) | 0 | 0.00 (0.00 - 0.00) |
| Juan de Acosta | 2 | 7.09 (0.84 - 54.84) | 0 | 0.00 (0.00 - 0.00) | 0 | 0.00 (0.00 - 0.00) |
| La Capilla | 0 | 0.00 (0.00 - 0.00) | 0 | 0.00 (0.00 - 0.00) | 1 | 0.23 (0.01 - 934.66) |
| La Ceja | 1 | 5.96 (0.15 - 29.47) | 0 | 0.00 (0.00 - 0.00) | 0 | 0.00 (0.00 - 0.00) |
| La Cruz | 2 | 18.28 (0.82 - 84.97) | 0 | 0.00 (0.00 - 0.00) | 0 | 0.00 (0.00 - 0.00) |
| La Dorada | 1 | 1.35 (0.03 - 15.05) | 1 | 0.34 (0.01 - 19.53) | 2 | 0.46 (0.01 - 14.35) |
| La Estrella | 4 | 42.72 (1.26 - 160.86) | 2 | 2.65 (0.14 - 21.15) | 1 | 9.62 (0.24 - 41.47) |
| La Florida | 0 | 0.00 (0.00 - 0.00) | 1 | 248.62 (6.29 - 973.17) | 0 | 0.00 (0.00 - 0.00) |
| La Gloria | 0 | 0.00 (0.00 - 0.00) | 2 | 19.32 (1.08 - 273.22) | 0 | 0.00 (0.00 - 0.00) |
| La Jagua de Ibirico | 1 | 5.73 (0.15 - 65.87) | 0 | 0.00 (0.00 - 0.00) | 1 | 24.04 (0.61 - 103.24) |
| La Mesa | 0 | 0.00 (0.00 - 0.00) | 1 | 0.66 (0.02 - 28.49) | 2 | 4.51 (0.35 - 39.25) |
| La Montañita | 1 | 4.14 (0.10 - 102.49) | 0 | 0.00 (0.00 - 0.00) | 0 | 0.00 (0.00 - 0.00) |
| La Palma | 2 | 23.33 (1.05 - 117.34) | 2 | 17.66 (1.18 - 164.11) | 1 | 2.42 (0.06 - 224.73) |
| La Peña | 0 | 0.00 (0.00 - 0.00) | 0 | 0.00 (0.00 - 0.00) | 2 | 399.60 (10.27 – 1,528.97) |
| La Pintada | 1 | 46.22 (1.17 - 226.96) | 0 | 0.00 (0.00 - 0.00) | 0 | 0.00 (0.00 - 0.00) |
| La Plata | 2 | 3.19 (0.37 - 23.04) | 3 | 6.31 (0.51 - 34.30) | 0 | 0.00 (0.00 - 0.00) |
| La Playa | 0 | 0.00 (0.00 - 0.00) | 1 | 49.50 (1.25 - 218.72) | 0 | 0.00 (0.00 - 0.00) |
| La Sierra | 2 | 3.83 (0.46 - 129.71) | 1 | 4.95 (0.13 - 110.73) | 0 | 0.00 (0.00 - 0.00) |
| La Tebaida | 2 | 22.96 (1.62 - 77.73) | 0 | 0.00 (0.00 - 0.00) | 3 | 18.75 (3.86 - 53.72) |
| La Unión (Antioquia) | 1 | 3.06 (0.08 - 44.85) | 0 | 0.00 (0.00 - 0.00) | 1 | 7.90 (0.2 - 58.01) |
| La Unión (Nariño) | 1 | 2.23 (0.06 - 38.82) | 0 | 0.00 (0.00 - 0.00) | 0 | 0.00 (0.00 - 0.00) |
| La Unión (Valle del Cauca) | 3 | 5.10 (0.68 - 42.73) | 0 | 0.00 (0.00 - 0.00) | 0 | 0.00 (0.00 - 0.00) |
| La Virginia | 4 | 28.98 (7.20 - 74.74) | 2 | 3.95 (0.20 - 32.19) | 1 | 2.51 (0.06 - 33.05) |
| Labateca | 1 | 9.29 (0.24 - 232.13) | 0 | 0.00 (0.00 - 0.00) | 0 | 0.00 (0.00 - 0.00) |
| Leguízamo | 0 | 0.00 (0.00 - 0.00) | 1 | 43.13 (1.09 - 184.26) | 0 | 0.00 (0.00 - 0.00) |
| Lenguazaque | 1 | 6.65 (0.17 - 287.22) | 0 | 0.00 (0.00 - 0.00) | 0 | 0.00 (0.00 - 0.00) |
| Lérida | 1 | 6.5 (0.16 - 60.37) | 1 | 9.92 (0.25 - 66.16) | 0 | 0.00 (0.00 - 0.00) |
| Leticia | 0 | 0.00 (0.00 - 0.00) | 1 | 54.55 (1.38 - 211.93) | 0 | 0.00 (0.00 - 0.00) |
| Líbano | 2 | 21.13 (1.59 - 75.76) | 1 | 2.63 (0.07 - 27.27) | 2 | 2.11 (0.26 - 21.44) |
| Liborina | 2 | 32.49 (3.63 - 137.98) | 0 | 0.00 (0.00 - 0.00) | 0 | 0.00 (0.00 - 0.00) |
| López | 1 | 8.46 (0.21 - 59.84) | 0 | 0.00 (0.00 - 0.00) | 0 | 0.00 (0.00 - 0.00) |
| Lorica | 3 | 1.75 (0.13 - 19.37) | 2 | 1.71 (0.13 - 9.45) | 1 | 1.01 (0.03 - 12.01) |
| Los Córdobas | 0 | 0.00 (0.00 - 0.00) | 2 | 7.40 (0.85 - 49.39) | 0 | 0.00 (0.00 - 0.00) |
| Los Patios | 2 | 20.34 (1.55 - 67.27) | 3 | 6.84 (1.06 - 23.01) | 1 | 0.92 (0.02 - 12.68) |
| Luruaco | 1 | 3.35 (0.08 - 46.94) | 1 | 7.55 (0.19 - 54.84) | 0 | 0.00 (0.00 - 0.00) |
| Madrid | 4 | 2.43 (0.57 - 27.41) | 0 | 0.00 (0.00 - 0.00) | 0 | 0.00 (0.00 - 0.00) |
| Magangué | 2 | 2.44 (0.30 - 12.88) | 5 | 5.56 (1.61 - 15.73) | 1 | 1.53 (0.04 - 12.57) |
| Mahates | 1 | 2.49 (0.06 - 82.1) | 1 | 1.13 (0.03 - 65.42) | 1 | 9.68 (0.25 - 51.62) |
| Maicao | 5 | 4.61 (1.36 - 12.69) | 5 | 5.79 (0.97 - 24.56) | 4 | 9.80 (2.00 - 25.77) |
| Majagual | 0 | 0.00 (0.00 - 0.00) | 0 | 0.00 (0.00 - 0.00) | 1 | 2.88 (0.07 - 32.20) |
| Málaga | 1 | 5.61 (0.14 - 48.18) | 0 | 0.00 (0.00 - 0.00) | 1 | 8.37 (0.21 - 62.41) |
| Malambo | 8 | 8.28 (3.15 - 18.32) | 5 | 13.29 (3.91 - 30.68) | 0 | 0.00 (0.00 - 0.00) |
| Mallama | 0 | 0.00 (0.00 - 0.00) | 0 | 0.00 (0.00 - 0.00) | 1 | 69.27 (1.75 - 312.55) |
| Manaure | 1 | 2.85 (0.07 - 73.77) | 1 | 7.32 (0.19 - 64.51) | 1 | 22.53 (0.57 - 119.64) |
| Manaure | 1 | 2.15 (0.05 - 12.89) | 1 | 3.82 (0.10 - 17.93) | 0 | 0.00 (0.00 - 0.00) |
| Manizales | 40 | 9.46 (3.09 - 19.97) | 10 | 0.17 (0.05 - 5.36) | 18 | 7.17 (3.79 - 12.36) |
| María La Baja | 2 | 1.18 (0.14 - 30.32) | 1 | 4.95 (0.13 - 31.05) | 1 | 0.96 (0.02 - 18.08) |
| Marinilla | 3 | 20.69 (1.13 - 73.07) | 1 | 4.69 (0.12 - 31.24) | 2 | 8.87 (0.50 - 35.97) |
| Mariquita | 0 | 0.00 (0.00 - 0.00) | 1 | 1.25 (0.03 - 35.15) | 0 | 0.00 (0.00 - 0.00) |
| Marsella | 1 | 1.82 (0.05 - 40.74) | 0 | 0.00 (0.00 - 0.00) | 0 | 0.00 (0.00 - 0.00) |
| Marulanda | 1 | 84.37 (2.14 - 451.20) | 0 | 0.00 (0.00 - 0.00) | 0 | 0.00 (0.00 - 0.00) |
| Medellín | 226 | 57.21 (44.5 - 71.61) | 70 | 2.74 (2.02 - 3.70) | 162 | 2.02 (1.68 - 2.76) |
| Mercaderes | 1 | 3.65 (0.09 - 86.48) | 0 | 0.00 (0.00 - 0.00) | 2 | 60.29 (3.61 - 209.44) |
| Milán | 1 | 0.16 (0.00 - 174.31) | 0 | 0.00 (0.00 - 0.00) | 0 | 0.00 (0.00 - 0.00) |
| Miraflores (Boyacá) | 0 | 0.00 (0.00 - 0.00) | 0 | 0.00 (0.00 - 0.00) | 1 | 25.42 (0.64 - 131.93) |
| Miraflores (Guaviare) | 0 | 0.00 (0.00 - 0.00) | 1 | 9.26 (0.23 - 105.22) | 0 | 0.00 (0.00 - 0.00) |
| Mocoa | 1 | 0.34 (0.01 - 32.34) | 1 | 4.17 (0.11 - 34.97) | 2 | 11.85 (0.44 - 48.31) |
| Momil | 0 | 0.00 (0.00 - 0.00) | 1 | 0.08 (0.00 - 72.81) | 0 | 0.00 (0.00 - 0.00) |
| Mompós | 5 | 8.25 (2.39 - 29.24) | 3 | 29.98 (1.83 - 102.80) | 2 | 6.17 (0.71 - 47.01) |
| Moniquirá | 0 | 0.00 (0.00 - 0.00) | 0 | 0.00 (0.00 - 0.00) | 2 | 3.60 (0.44 - 47.51) |
| Montelíbano | 3 | 6.94 (1.21 - 21.28) | 1 | 0.36 (0.01 - 55.81) | 1 | 5.04 (0.13 - 25.76) |
| Montenegro | 3 | 15.04 (2.88 - 49.83) | 2 | 8.02 (0.91 - 44.42) | 3 | 21.52 (1.99 - 73.12) |
| Montería | 26 | 17.51 (8.76 - 29.79) | 17 | 2.58 (1.31 - 5.52) | 16 | 6.97 (3.49 - 12.23) |
| Moñitos | 1 | 5.14 (0.13 - 67.08) | 2 | 14.53 (0.73 - 60.34) | 2 | 17.97 (1.63 - 66.04) |
| Morales | 0 | 0.00 (0.00 - 0.00) | 1 | 29.43 (0.75 - 127.32) | 0 | 0.00 (0.00 - 0.00) |
| Morroa | 0 | 0.00 (0.00 - 0.00) | 0 | 0.00 (0.00 - 0.00) | 1 | 3.31 (0.08 - 79.65) |
| Mosquera (Cundinamarca) | 2 | 4.33 (0.52 - 18.29) | 1 | 3.68 (0.09 - 23.84) | 1 | 0.30 (0.01 - 18.30) |
| Muzo | 0 | 0.00 (0.00 - 0.00) | 1 | 5.41 (0.14 - 105.77) | 0 | 0.00 (0.00 - 0.00) |
| Natagaima | 1 | 4.37 (0.11 - 53.42) | 0 | 0.00 (0.00 - 0.00) | 0 | 0.00 (0.00 - 0.00) |
| Necoclí | 1 | 0.53 (0.01 - 18.95) | 1 | 1.02 (0.03 - 21.15) | 0 | 0.00 (0.00 - 0.00) |
| Neira | 1 | 6.32 (0.16 - 49.01) | 0 | 0.00 (0.00 - 0.00) | 0 | 0.00 (0.00 - 0.00) |
| Neiva | 30 | 14.37 (9.27 - 21.21) | 7 | 6.11 (0.68 - 18.22) | 27 | 8.02 (4.86 - 14.12) |
| Nemocón | 1 | 30.32 (0.77 - 137.55) | 0 | 0.00 (0.00 - 0.00) | 0 | 0.00 (0.00 - 0.00) |
| Nimaima | 2 | 1.16 (0.14 - 211.27) | 0 | 0.00 (0.00 - 0.00) | 0 | 0.00 (0.00 - 0.00) |
| Nobsa | 1 | 2.39 (0.06 - 231.96) | 0 | 0.00 (0.00 - 0.00) | 0 | 0.00 (0.00 - 0.00) |
| Ocaña | 7 | 12.94 (4.56 - 28.67) | 2 | 2.15 (0.11 - 12.66) | 5 | 5.41 (1.61 - 16.48) |
| Oiba | 1 | 3.76 (0.10 - 174.11) | 1 | 0.50 (0.01 - 139.23) | 0 | 0.00 (0.00 - 0.00) |
| Olaya Herrera | 0 | 0.00 (0.00 - 0.00) | 0 | 0.00 (0.00 - 0.00) | 1 | 8.07 (0.20 - 136.9) |
| Onzaga | 1 | 20.61 (0.52 - 220.03) | 0 | 0.00 (0.00 - 0.00) | 0 | 0.00 (0.00 - 0.00) |
| Oporapa | 1 | 0.29 (0.01 - 157.12) | 0 | 0.00 (0.00 - 0.00) | 0 | 0.00 (0.00 - 0.00) |
| Orito | 1 | 4.02 (0.10 - 68.23) | 0 | 0.00 (0.00 - 0.00) | 0 | 0.00 (0.00 - 0.00) |
| Ortega | 0 | 0.00 (0.00 - 0.00) | 0 | 0.00 (0.00 - 0.00) | 1 | 1.70 (0.04 - 29.79) |
| Ovejas | 0 | 0.00 (0.00 - 0.00) | 0 | 0.00 (0.00 - 0.00) | 1 | 37.50 (0.95 - 151.87) |
| Pacho | 3 | 12.32 (1.95 - 48.46) | 0 | 0.00 (0.00 - 0.00) | 1 | 6.23 (0.16 - 44.64) |
| Pácora | 1 | 23.49 (0.59 - 136.18) | 0 | 0.00 (0.00 - 0.00) | 3 | 46.27 (4.44 - 189.85) |
| Páez | 0 | 0.00 (0.00 - 0.00) | 0 | 0.00 (0.00 - 0.00) | 1 | 27.68 (0.70 - 868.35) |
| Pailitas | 1 | 2.36 (0.06 - 61.37) | 2 | 9.90 (1.20 - 74.19) | 0 | 0.00 (0.00 - 0.00) |
| Paipa | 1 | 2.45 (0.06 - 48.69) | 0 | 0.00 (0.00 - 0.00) | 0 | 0.00 (0.00 - 0.00) |
| Palermo | 1 | 1.48 (0.04 - 28.96) | 0 | 0.00 (0.00 - 0.00) | 0 | 0.00 (0.00 - 0.00) |
| Palestina (Huila) | 1 | 3.34 (0.08 - 361.9) | 0 | 0.00 (0.00 - 0.00) | 0 | 0.00 (0.00 - 0.00) |
| Palmira | 32 | 3.58 (2.12 - 8.19) | 7 | 0.71 (0.12 - 5.94) | 16 | 9.34 (5.26 - 15.82) |
| Pamplona | 0 | 0.00 (0.00 - 0.00) | 0 | 0.00 (0.00 - 0.00) | 3 | 8.32 (1.72 - 29.23) |
| Pamplonita | 1 | 22.31 (0.56 - 222.85) | 0 | 0.00 (0.00 - 0.00) | 0 | 0.00 (0.00 - 0.00) |
| Páramo | 0 | 0.00 (0.00 - 0.00) | 1 | 22.02 (0.56 - 220.08) | 0 | 0.00 (0.00 - 0.00) |
| Pasto | 31 | 17.89 (7.21 - 33.93) | 20 | 4.33 (1.88 - 8.51) | 20 | 2.25 (1.35 - 4.96) |
| Pauna | 0 | 0.00 (0.00 - 0.00) | 1 | 1.66 (0.04 - 257.93) | 0 | 0.00 (0.00 - 0.00) |
| Paz de Ariporo | 3 | 54.25 (3.37 - 185.96) | 0 | 0.00 (0.00 - 0.00) | 0 | 0.00 (0.00 - 0.00) |
| Pensilvania | 0 | 0.00 (0.00 - 0.00) | 1 | 3.35 (0.08 - 36.56) | 0 | 0.00 (0.00 - 0.00) |
| Peñol | 2 | 12.54 (1.48 - 89.44) | 1 | 17.13 (0.43 - 91.26) | 0 | 0.00 (0.00 - 0.00) |
| Pereira | 41 | 17.25 (10.73 - 25.78) | 8 | 2.63 (0.78 - 6.22) | 22 | 3.90 (2.28 - 6.88) |
| Piedecuesta | 5 | 1.87 (0.49 - 7.55) | 6 | 5.80 (0.88 – 17.00) | 2 | 1.82 (0.16 - 9.62) |
| Piendamó | 0 | 0.00 (0.00 - 0.00) | 2 | 13.90 (0.46 - 55.82) | 0 | 0.00 (0.00 - 0.00) |
| Pinillos | 0 | 0.00 (0.00 - 0.00) | 1 | 1.48 (0.04 - 145.87) | 1 | 4.69 (0.12 - 37.16) |
| Piojó | 0 | 0.00 (0.00 - 0.00) | 1 | 24.47 (0.62 - 221.62) | 0 | 0.00 (0.00 - 0.00) |
| Pital | 1 | 8.70 (0.22 - 106.6) | 0 | 0.00 (0.00 - 0.00) | 0 | 0.00 (0.00 - 0.00) |
| Pitalito | 4 | 17.38 (2.74 - 48.11) | 4 | 3.29 (0.83 - 18.09) | 4 | 9.05 (1.06 - 28.14) |
| Pivijay | 0 | 0.00 (0.00 - 0.00) | 1 | 0.96 (0.02 - 33.86) | 0 | 0.00 (0.00 - 0.00) |
| Planadas | 1 | 0.64 (0.02 - 59.2) | 0 | 0.00 (0.00 - 0.00) | 0 | 0.00 (0.00 - 0.00) |
| Planeta Rica | 5 | 25.88 (1.13 - 92.58) | 2 | 8.19 (0.73 - 31.27) | 1 | 0.58 (0.01 - 20.32) |
| Plato | 0 | 0.00 (0.00 - 0.00) | 1 | 4.34 (0.11 - 21.89) | 0 | 0.00 (0.00 - 0.00) |
| Policarpa | 2 | 93.69 (2.39 - 362.82) | 0 | 0.00 (0.00 - 0.00) | 1 | 1.31 (0.03 - 68.09) |
| Ponedera | 1 | 1.77 (0.04 - 37.81) | 1 | 2.02 (0.05 - 58.08) | 1 | 29.39 (0.74 - 126.21) |
| Popayán | 14 | 2.35 (0.85 - 6.27) | 7 | 5.17 (1.87 - 11.02) | 14 | 49.39 (20.79 - 91.21) |
| Pradera | 3 | 3.43 (0.55 - 19.41) | 4 | 7.75 (1.27 - 26.66) | 0 | 0.00 (0.00 - 0.00) |
| Pueblo Bello | 2 | 29.72 (1.42 - 114.2) | 0 | 0.00 (0.00 - 0.00) | 0 | 0.00 (0.00 - 0.00) |
| Puebloviejo | 0 | 0.00 (0.00 - 0.00) | 0 | 0.00 (0.00 - 0.00) | 1 | 3.99 (0.10 - 43.44) |
| Puerto Asís | 2 | 4.97 (0.60 - 79.81) | 2 | 11.57 (1.26 - 40.51) | 0 | 0.00 (0.00 - 0.00) |
| Puerto Berrío | 0 | 0.00 (0.00 - 0.00) | 3 | 18.16 (3.62 - 54.12) | 0 | 0.00 (0.00 - 0.00) |
| Puerto Boyacá | 1 | 2.15 (0.05 - 31.27) | 2 | 4.57 (0.17 - 26.71) | 2 | 3.51 (0.42 - 18.47) |
| Puerto Carreño | 0 | 0.00 (0.00 - 0.00) | 1 | 2.05 (0.05 - 351.03) | 0 | 0.00 (0.00 - 0.00) |
| Puerto Escondido | 1 | 1.86 (0.05 - 49.1) | 0 | 0.00 (0.00 - 0.00) | 1 | 2.69 (0.07 - 31.46) |
| Puerto Libertador | 1 | 1.53 (0.04 - 31.16) | 2 | 1.62 (0.08 - 185.61) | 0 | 0.00 (0.00 - 0.00) |
| Puerto López | 0 | 0.00 (0.00 - 0.00) | 2 | 14.50 (0.85 - 58.23) | 0 | 0.00 (0.00 - 0.00) |
| Puerto Nare | 1 | 0.84 (0.02 - 71.53) | 0 | 0.00 (0.00 - 0.00) | 0 | 0.00 (0.00 - 0.00) |
| Puerto Rico (Caquetá) | 3 | 12.39 (2.46 - 48.7) | 0 | 0.00 (0.00 - 0.00) | 0 | 0.00 (0.00 - 0.00) |
| Puerto Salgar | 0 | 0.00 (0.00 - 0.00) | 0 | 0.00 (0.00 - 0.00) | 1 | 0.88 (0.02 - 84.84) |
| Puerto Tejada | 5 | 39.53 (9.24 - 99.18) | 1 | 2.40 (0.06 - 23.76) | 3 | 11.96 (1.47 - 43.27) |
| Puerto Triunfo | 1 | 37.07 (0.94 - 172.88) | 0 | 0.00 (0.00 - 0.00) | 1 | 2.14 (0.05 - 55.49) |
| Puerto Wilches | 1 | 181.41 (4.59 - 691.82) | 0 | 0.00 (0.00 - 0.00) | 0 | 0.00 (0.00 - 0.00) |
| Pupiales | 0 | 0.00 (0.00 - 0.00) | 1 | 12.80 (0.32 - 70.17) | 2 | 5.71 (0.67 - 55.56) |
| Purificación | 1 | 1.06 (0.03 - 50.59) | 1 | 2.14 (0.05 - 48.02) | 1 | 2.94 (0.07 - 33.03) |
| Quetame | 0 | 0.00 (0.00 - 0.00) | 1 | 7.93 (0.20 - 189.91) | 0 | 0.00 (0.00 - 0.00) |
| Quibdó | 3 | 3.75 (0.40 - 16.85) | 1 | 0.61 (0.02 - 18.82) | 4 | 13.00 (3.54 - 31.98) |
| Quimbaya | 1 | 7.99 (0.20 - 45.70) | 2 | 10.67 (1.27 - 42.37) | 2 | 3.46 (0.27 - 37.05) |
| Quípama | 1 | 28.92 (0.73 - 175.4) | 0 | 0.00 (0.00 - 0.00) | 0 | 0.00 (0.00 - 0.00) |
| Quipile | 1 | 20.90 (0.53 - 158.4) | 0 | 0.00 (0.00 - 0.00) | 0 | 0.00 (0.00 - 0.00) |
| Regidor | 1 | 64.93 (1.64 - 274.65) | 0 | 0.00 (0.00 - 0.00) | 0 | 0.00 (0.00 - 0.00) |
| Remedios | 1 | 4.55 (0.12 - 59.21) | 0 | 0.00 (0.00 - 0.00) | 1 | 0.70 (0.02 - 38.12) |
| Remolino | 1 | 12.25 (0.31 - 126.71) | 0 | 0.00 (0.00 - 0.00) | 0 | 0.00 (0.00 - 0.00) |
| Restrepo (Meta) | 1 | 8.80 (0.22 - 118.28) | 0 | 0.00 (0.00 - 0.00) | 0 | 0.00 (0.00 - 0.00) |
| Retiro | 0 | 0.00 (0.00 - 0.00) | 2 | 35.87 (3.61 - 130.5) | 0 | 0.00 (0.00 - 0.00) |
| Río de Oro | 0 | 0.00 (0.00 - 0.00) | 1 | 1.49 (0.04 - 91.87) | 0 | 0.00 (0.00 - 0.00) |
| Riohacha | 6 | 1.33 (0.46 - 4.53) | 4 | 9.96 (1.55 - 27.51) | 2 | 0.62 (0.07 - 4.35) |
| Rionegro (Antioquia) | 7 | 11.35 (3.32 - 26.97) | 2 | 0.42 (0.04 - 9.23) | 5 | 29.46 (8.47 - 66.22) |
| Riosucio (Caldas) | 2 | 3.45 (0.31 - 17.82) | 1 | 0.04 (0.00 - 27.50) | 2 | 1.78 (0.22 - 16.13) |
| Rivera | 3 | 7.99 (0.96 - 75.83) | 0 | 0.00 (0.00 - 0.00) | 2 | 0.56 (0.07 - 106.63) |
| Roldanillo | 1 | 1.57 (0.04 - 27.03) | 0 | 0.00 (0.00 - 0.00) | 4 | 20.04 (3.18 - 63.83) |
| Rosas | 2 | 10.20 (0.84 - 156.35) | 0 | 0.00 (0.00 - 0.00) | 0 | 0.00 (0.00 - 0.00) |
| Rovira | 1 | 1.45 (0.04 - 75.53) | 1 | 1.56 (0.04 - 85.91) | 0 | 0.00 (0.00 - 0.00) |
| Sabanagrande | 1 | 76.48 (1.94 - 295.66) | 0 | 0.00 (0.00 - 0.00) | 0 | 0.00 (0.00 - 0.00) |
| Sabanalarga (Antioquia) | 1 | 3.73 (0.09 - 170.55) | 0 | 0.00 (0.00 - 0.00) | 0 | 0.00 (0.00 - 0.00) |
| Sabanalarga (Atlántico) | 3 | 6.09 (1.05 - 18.93) | 1 | 4.38 (0.11 - 22.71) | 2 | 0.26 (0.03 - 19.08) |
| Sabanas de San Ángel | 1 | 8.52 (0.22 - 62.62) | 0 | 0.00 (0.00 - 0.00) | 0 | 0.00 (0.00 - 0.00) |
| Sabaneta | 7 | 6.56 (1.09 - 30.97) | 2 | 4.40 (0.34 - 24.50) | 4 | 30.14 (6.9 - 77.64) |
| Saboyá | 1 | 41.89 (1.06 - 186.17) | 0 | 0.00 (0.00 - 0.00) | 0 | 0.00 (0.00 - 0.00) |
| Sácama | 0 | 0.00 (0.00 - 0.00) | 1 | 79.98 (2.02 - 568.76) | 0 | 0.00 (0.00 - 0.00) |
| Sahagún | 4 | 1.50 (0.24 - 11.93) | 5 | 1.27 (0.37 - 18.13) | 2 | 2.83 (0.34 - 13.89) |
| Salamina | 0 | 0.00 (0.00 - 0.00) | 0 | 0.00 (0.00 - 0.00) | 5 | 73.08 (23.73 - 255.24) |
| Salamina | 1 | 14.98 (0.38 - 171.30) | 0 | 0.00 (0.00 - 0.00) | 0 | 0.00 (0.00 - 0.00) |
| Salazar | 1 | 3.36 (0.08 - 152.28) | 0 | 0.00 (0.00 - 0.00) | 0 | 0.00 (0.00 - 0.00) |
| Samacá | 1 | 2.31 (0.06 - 65.85) | 0 | 0.00 (0.00 - 0.00) | 0 | 0.00 (0.00 - 0.00) |
| Samaná | 1 | 8.54 (0.22 - 78.37) | 0 | 0.00 (0.00 - 0.00) | 0 | 0.00 (0.00 - 0.00) |
| Samaniego | 0 | 0.00 (0.00 - 0.00) | 2 | 2.81 (0.18 - 28.07) | 0 | 0.00 (0.00 - 0.00) |
| Sampués | 1 | 5.15 (0.13 - 54.59) | 1 | 1.91 (0.05 - 27.72) | 1 | 3.33 (0.08 - 27.31) |
| San Agustín | 3 | 15.94 (1.00 - 77.66) | 0 | 0.00 (0.00 - 0.00) | 0 | 0.00 (0.00 - 0.00) |
| San Alberto | 0 | 0.00 (0.00 - 0.00) | 1 | 10.29 (0.26 - 76.04) | 0 | 0.00 (0.00 - 0.00) |
| San Andrés | 1 | 5.20 (0.13 - 179.48) | 0 | 0.00 (0.00 - 0.00) | 5 | 9.75 (2.13 - 31.48) |
| San Andrés | 3 | 5.99 (0.76 - 31.96) | 0 | 0.00 (0.00 - 0.00) | 1 | 21.41 (0.54 - 201.94) |
| San Andrés de Cuerquía | 1 | 11.95 (0.30 - 319.54) | 0 | 0.00 (0.00 - 0.00) | 0 | 0.00 (0.00 - 0.00) |
| San Andrés de Tumaco | 9 | 12.15 (3.32 - 28.86) | 7 | 14.37 (4.80 - 30.72) | 2 | 4.12 (0.4 - 13.70) |
| San Andrés Sotavento | 1 | 74.76 (1.89 - 288.91) | 1 | 0.48 (0.01 - 30.51) | 0 | 0.00 (0.00 - 0.00) |
| San Antero | 0 | 0.00 (0.00 - 0.00) | 0 | 0.00 (0.00 - 0.00) | 4 | 21.65 (5.85 - 68.82) |
| San Antonio | 1 | 1.26 (0.03 - 127.30) | 0 | 0.00 (0.00 - 0.00) | 0 | 0.00 (0.00 - 0.00) |
| San Benito Abad | 1 | 1.32 (0.03 - 51.26) | 0 | 0.00 (0.00 - 0.00) | 0 | 0.00 (0.00 - 0.00) |
| San Bernardo | 0 | 0.00 (0.00 - 0.00) | 1 | 2.40 (0.06 - 121.71) | 0 | 0.00 (0.00 - 0.00) |
| San Carlos | 0 | 0.00 (0.00 - 0.00) | 0 | 0.00 (0.00 - 0.00) | 1 | 2.53 (0.06 - 52.93) |
| San Cayetano | 0 | 0.00 (0.00 - 0.00) | 1 | 12.08 (0.31 - 195.45) | 0 | 0.00 (0.00 - 0.00) |
| San Diego | 1 | 5.31 (0.13 - 66.39) | 0 | 0.00 (0.00 - 0.00) | 0 | 0.00 (0.00 - 0.00) |
| San Estanislao | 0 | 0.00 (0.00 - 0.00) | 1 | 3.01 (0.08 - 112.82) | 0 | 0.00 (0.00 - 0.00) |
| San Gil | 3 | 6.39 (1.11 - 27.50) | 0 | 0.00 (0.00 - 0.00) | 1 | 0.31 (0.01 - 43.29) |
| San Jacinto | 0 | 0.00 (0.00 - 0.00) | 1 | 4.75 (0.12 - 43.57) | 1 | 1.03 (0.03 - 43.78) |
| San Jacinto del Cauca | 1 | 52.62 (1.33 - 220.93) | 0 | 0.00 (0.00 - 0.00) | 0 | 0.00 (0.00 - 0.00) |
| San Jerónimo | 0 | 0.00 (0.00 - 0.00) | 0 | 0.00 (0.00 - 0.00) | 1 | 5.15 (0.13 - 79.44) |
| San Joaquín | 1 | 41.33 (1.05 - 710.49) | 0 | 0.00 (0.00 - 0.00) | 0 | 0.00 (0.00 - 0.00) |
| San José de Pare | 0 | 0.00 (0.00 - 0.00) | 0 | 0.00 (0.00 - 0.00) | 1 | 12.00 (0.30 - 560.68) |
| San José del Fragua | 1 | 12.43 (0.31 - 84.94) | 0 | 0.00 (0.00 - 0.00) | 0 | 0.00 (0.00 - 0.00) |
| San José del Guaviare | 0 | 0.00 (0.00 - 0.00) | 2 | 0.89 (0.10 - 63.32) | 0 | 0.00 (0.00 - 0.00) |
| San Juan de Arama | 0 | 0.00 (0.00 - 0.00) | 1 | 2.35 (0.06 - 143.82) | 0 | 0.00 (0.00 - 0.00) |
| San Juan de Río Seco | 0 | 0.00 (0.00 - 0.00) | 0 | 0.00 (0.00 - 0.00) | 1 | 2.92 (0.07 - 114.54) |
| San Juan de Urabá | 0 | 0.00 (0.00 - 0.00) | 1 | 0.43 (0.01 - 40.03) | 0 | 0.00 (0.00 - 0.00) |
| San Juan del Cesar | 0 | 0.00 (0.00 - 0.00) | 2 | 11.73 (0.78 - 48.73) | 1 | 16.14 (0.41 - 78.38) |
| San Juan Nepomuceno | 5 | 2.66 (0.84 - 41.17) | 0 | 0.00 (0.00 - 0.00) | 1 | 3.32 (0.08 - 40.29) |
| San Luis de Sincé | 0 | 0.00 (0.00 - 0.00) | 1 | 3.46 (0.09 - 31.72) | 1 | 3.86 (0.10 - 32.71) |
| San Marcos | 2 | 4.49 (0.42 - 23.47) | 1 | 1.81 (0.05 - 57.33) | 0 | 0.00 (0.00 - 0.00) |
| San Martín | 0 | 0.00 (0.00 - 0.00) | 0 | 0.00 (0.00 - 0.00) | 2 | 19.90 (1.79 - 89.30) |
| San Martín | 1 | 21.56 (0.55 - 93.87) | 0 | 0.00 (0.00 - 0.00) | 0 | 0.00 (0.00 - 0.00) |
| San Martín de Loba | 0 | 0.00 (0.00 - 0.00) | 1 | 5.73 (0.15 - 73.36) | 0 | 0.00 (0.00 - 0.00) |
| San Miguel de Sema | 0 | 0.00 (0.00 - 0.00) | 0 | 0.00 (0.00 - 0.00) | 1 | 8.64 (0.22 - 261.43) |
| San Onofre | 1 | 2.12 (0.05 - 21.61) | 0 | 0.00 (0.00 - 0.00) | 1 | 0.57 (0.01 - 36.7) |
| San Pablo | 0 | 0.00 (0.00 - 0.00) | 1 | 3.69 (0.09 - 233.22) | 0 | 0.00 (0.00 - 0.00) |
| San Pablo | 2 | 13.39 (1.30 - 74.24) | 0 | 0.00 (0.00 - 0.00) | 0 | 0.00 (0.00 - 0.00) |
| San Pedro | 3 | 18.84 (0.97 - 74.91) | 1 | 5.14 (0.13 - 60.54) | 1 | 8.09 (0.20 - 77.87) |
| San Pedro | 2 | 25.00 (1.23 - 181.47) | 0 | 0.00 (0.00 - 0.00) | 0 | 0.00 (0.00 - 0.00) |
| San Pedro de Urabá | 1 | 0.85 (0.02 - 152.52) | 1 | 3.80 (0.10 - 61.82) | 1 | 23.16 (0.59 - 98.38) |
| San Pelayo | 0 | 0.00 (0.00 - 0.00) | 0 | 0.00 (0.00 - 0.00) | 1 | 0.49 (0.01 - 24.94) |
| San Roque | 1 | 15.70 (0.40 - 95.14) | 0 | 0.00 (0.00 - 0.00) | 1 | 3.51 (0.09 - 59.62) |
| San Sebastián | 0 | 0.00 (0.00 - 0.00) | 1 | 3.93 (0.10 - 70.32) | 1 | 2.66 (0.07 - 48.58) |
| San Vicente | 0 | 0.00 (0.00 - 0.00) | 1 | 6.24 (0.16 - 68.24) | 0 | 0.00 (0.00 - 0.00) |
| San Vicente de Chucurí | 1 | 1.48 (0.04 - 32.19) | 0 | 0.00 (0.00 - 0.00) | 1 | 1.27 (0.03 - 26.06) |
| San Vicente del Caguán | 1 | 1.33 (0.03 - 16.93) | 5 | 8.55 (1.22 - 28.38) | 1 | 4.80 (0.12 - 22.54) |
| Sandoná | 0 | 0.00 (0.00 - 0.00) | 1 | 0.65 (0.02 - 57.52) | 0 | 0.00 (0.00 - 0.00) |
| Santa Ana | 0 | 0.00 (0.00 - 0.00) | 0 | 0.00 (0.00 - 0.00) | 1 | 6.20 (0.16 - 47.79) |
| Santa Bárbara | 0 | 0.00 (0.00 - 0.00) | 1 | 12.24 (0.31 - 82.25) | 0 | 0.00 (0.00 - 0.00) |
| Santa Bárbara | 3 | 2.27 (0.46 - 79.53) | 0 | 0.00 (0.00 - 0.00) | 0 | 0.00 (0.00 - 0.00) |
| Santa Catalina | 1 | 8.91 (0.23 - 139.57) | 1 | 6.67 (0.17 - 330.14) | 1 | 32.27 (0.82 - 172.27) |
| Santa Isabel | 0 | 0.00 (0.00 - 0.00) | 1 | 4.73 (0.12 - 211.53) | 0 | 0.00 (0.00 - 0.00) |
| Santa María | 0 | 0.00 (0.00 - 0.00) | 0 | 0.00 (0.00 - 0.00) | 1 | 4.00 (0.10 - 95.32) |
| Santa María | 2 | 72.69 (4.09 - 267.41) | 0 | 0.00 (0.00 - 0.00) | 0 | 0.00 (0.00 - 0.00) |
| Santa Marta | 35 | 12.55 (4.40 - 25.40) | 5 | 1.10 (0.33 - 3.69) | 25 | 7.19 (4.51 - 11.18) |
| Santa Rosa | 1 | 21.64 (0.55 - 103.32) | 2 | 29.83 (3.61 - 113.12) | 1 | 1.75 (0.04 - 38.18) |
| Santa Rosa | 0 | 0.00 (0.00 - 0.00) | 1 | 9.12 (0.23 - 447.48) | 0 | 0.00 (0.00 - 0.00) |
| Santa Rosa de Cabal | 5 | 6.2 (1.33 - 22.30) | 1 | 0.53 (0.01 - 17.52) | 2 | 6.91 (0.69 - 25.76) |
| Santa Rosa de Osos | 1 | 0.81 (0.02 - 198.25) | 0 | 0.00 (0.00 - 0.00) | 2 | 4.38 (0.47 - 25.79) |
| Santa Rosa del Sur | 1 | 0.84 (0.02 - 24.71) | 1 | 5.63 (0.14 - 29.73) | 0 | 0.00 (0.00 - 0.00) |
| Santacruz | 0 | 0.00 (0.00 - 0.00) | 0 | 0.00 (0.00 - 0.00) | 1 | 12.98 (0.33 - 60.48) |
| Santafé de Antioquia | 2 | 14.95 (1.81 - 69.10) | 0 | 0.00 (0.00 - 0.00) | 0 | 0.00 (0.00 - 0.00) |
| Santander de Quilichao | 3 | 1.91 (0.24 - 9.94) | 1 | 26.15 (0.66 - 100.60) | 4 | 8.12 (1.58 - 23.40) |
| Santiago | 1 | 14.02 (0.35 - 257.90) | 0 | 0.00 (0.00 - 0.00) | 0 | 0.00 (0.00 - 0.00) |
| Santiago de Tolú | 4 | 22.22 (2.39 - 72.40) | 0 | 0.00 (0.00 - 0.00) | 1 | 4.19 (0.11 - 50.88) |
| Santo Tomás | 2 | 4.43 (0.22 - 42.98) | 0 | 0.00 (0.00 - 0.00) | 1 | 3.17 (0.08 - 38.57) |
| Santuario | 0 | 0.00 (0.00 - 0.00) | 1 | 2.19 (0.06 - 137.57) | 0 | 0.00 (0.00 - 0.00) |
| Saravena | 3 | 3.13 (0.20 - 33.46) | 2 | 7.03 (0.55 - 53.15) | 0 | 0.00 (0.00 - 0.00) |
| Sardinata | 0 | 0.00 (0.00 - 0.00) | 1 | 13.76 (0.35 - 66.93) | 0 | 0.00 (0.00 - 0.00) |
| Sasaima | 0 | 0.00 (0.00 - 0.00) | 0 | 0.00 (0.00 - 0.00) | 1 | 1.09 (0.03 - 78.15) |
| Sativasur | 0 | 0.00 (0.00 - 0.00) | 0 | 0.00 (0.00 - 0.00) | 1 | 23.26 (0.59 - 1234.21) |
| Segovia | 2 | 3.76 (0.14 - 27.36) | 1 | 4.77 (0.12 - 35.7) | 0 | 0.00 (0.00 - 0.00) |
| Sevilla | 1 | 11.73 (0.30 - 51.82) | 1 | 0.77 (0.02 - 28.93) | 1 | 0.54 (0.01 - 30.79) |
| Sibaté | 2 | 7.02 (0.68 - 29.77) | 0 | 0.00 (0.00 - 0.00) | 1 | 40.46 (1.02 - 157.18) |
| Sibundoy | 0 | 0.00 (0.00 - 0.00) | 1 | 7.25 (0.18 - 66.46) | 1 | 2.36 (0.06 - 79.24) |
| Silos | 0 | 0.00 (0.00 - 0.00) | 1 | 25.51 (0.65 - 443.83) | 0 | 0.00 (0.00 - 0.00) |
| Silvania | 1 | 4.98 (0.13 - 43.55) | 1 | 5.23 (0.13 - 59.84) | 0 | 0.00 (0.00 - 0.00) |
| Silvia | 1 | 10.31 (0.26 - 50.15) | 0 | 0.00 (0.00 - 0.00) | 0 | 0.00 (0.00 - 0.00) |
| Simacota | 1 | 14.60 (0.37 - 175.31) | 0 | 0.00 (0.00 - 0.00) | 0 | 0.00 (0.00 - 0.00) |
| Simijaca | 1 | 7.63 (0.19 - 223.85) | 0 | 0.00 (0.00 - 0.00) | 0 | 0.00 (0.00 - 0.00) |
| Simití | 2 | 36.20 (2.83 - 130.82) | 0 | 0.00 (0.00 - 0.00) | 0 | 0.00 (0.00 - 0.00) |
| Sincelejo | 18 | 1.74 (0.96 - 5.46) | 10 | 2.43 (0.68 - 8.05) | 9 | 4.32 (1.78 - 9.02) |
| Soacha | 8 | 0.84 (0.23 - 5.14) | 6 | 0.31 (0.08 - 2.91) | 10 | 5.96 (2.21 - 12.13) |
| Soatá | 0 | 0.00 (0.00 - 0.00) | 0 | 0.00 (0.00 - 0.00) | 1 | 6.71 (0.17 - 166.1) |
| Socha | 0 | 0.00 (0.00 - 0.00) | 0 | 0.00 (0.00 - 0.00) | 1 | 782.39 (19.81 - 2987.81) |
| Socorro | 1 | 21.52 (0.54 - 93.33) | 0 | 0.00 (0.00 - 0.00) | 0 | 0.00 (0.00 - 0.00) |
| Sogamoso | 6 | 8.81 (2.37 - 22.04) | 1 | 0.93 (0.02 - 18.35) | 1 | 2.19 (0.06 - 14.58) |
| Solano | 0 | 0.00 (0.00 - 0.00) | 1 | 4.96 (0.13 - 103.13) | 0 | 0.00 (0.00 - 0.00) |
| Soledad | 17 | 10.19 (1.41 - 28.51) | 9 | 5.88 (1.12 - 14.84) | 11 | 0.39 (0.19 - 1.94) |
| Solita | 0 | 0.00 (0.00 - 0.00) | 1 | 19.49 (0.49 - 128.37) | 0 | 0.00 (0.00 - 0.00) |
| Sonson | 1 | 3.66 (0.09 - 31.69) | 0 | 0.00 (0.00 - 0.00) | 0 | 0.00 (0.00 - 0.00) |
| Sora | 0 | 0.00 (0.00 - 0.00) | 0 | 0.00 (0.00 - 0.00) | 1 | 10.09 (0.26 - 660.74) |
| Suaita | 0 | 0.00 (0.00 - 0.00) | 1 | 20.64 (0.52 - 161.57) | 0 | 0.00 (0.00 - 0.00) |
| Suan | 0 | 0.00 (0.00 - 0.00) | 0 | 0.00 (0.00 - 0.00) | 1 | 62.34 (1.58 - 266.68) |
| Suárez | 0 | 0.00 (0.00 - 0.00) | 0 | 0.00 (0.00 - 0.00) | 1 | 3.08 (0.08 - 272.25) |
| Suaza | 0 | 0.00 (0.00 - 0.00) | 0 | 0.00 (0.00 - 0.00) | 1 | 6.47 (0.16 - 71.97) |
| Sucre | 1 | 1.81 (0.05 - 149.17) | 0 | 0.00 (0.00 - 0.00) | 0 | 0.00 (0.00 - 0.00) |
| Suesca | 0 | 0.00 (0.00 - 0.00) | 0 | 0.00 (0.00 - 0.00) | 1 | 3.05 (0.08 - 63.04) |
| Supía | 2 | 9.63 (1.14 - 53.45) | 2 | 10.22 (1.10 - 51.91) | 3 | 12.42 (0.98 - 61.19) |
| Susacón | 1 | 55.68 (1.41 - 403.04) | 0 | 0.00 (0.00 - 0.00) | 1 | 6.59 (0.17 - 297.19) |
| Sutamarchán | 1 | 8.54 (0.22 - 234.25) | 0 | 0.00 (0.00 - 0.00) | 1 | 7.17 (0.18 - 171.95) |
| Tabio | 1 | 0.52 (0.01 - 74.91) | 0 | 0.00 (0.00 - 0.00) | 0 | 0.00 (0.00 - 0.00) |
| Tadó | 1 | 8.02 (0.20 - 60.98) | 0 | 0.00 (0.00 - 0.00) | 1 | 86.28 (2.18 - 342.67) |
| Tamalameque | 0 | 0.00 (0.00 - 0.00) | 0 | 0.00 (0.00 - 0.00) | 1 | 20.87 (0.53 - 121.14) |
| Tame | 0 | 0.00 (0.00 - 0.00) | 1 | 5.84 (0.15 - 41.18) | 0 | 0.00 (0.00 - 0.00) |
| Támesis | 1 | 18.74 (0.47 - 125.85) | 0 | 0.00 (0.00 - 0.00) | 0 | 0.00 (0.00 - 0.00) |
| Taminango | 0 | 0.00 (0.00 - 0.00) | 1 | 6.89 (0.17 - 62.59) | 1 | 1.24 (0.03 - 46.99) |
| Tangua | 0 | 0.00 (0.00 - 0.00) | 0 | 0.00 (0.00 - 0.00) | 3 | 31.13 (3.29 - 147.43) |
| Tarqui | 0 | 0.00 (0.00 - 0.00) | 3 | 101.39 (5.10 - 355.92) | 2 | 32.25 (1.70 - 129.63) |
| Tauramena | 0 | 0.00 (0.00 - 0.00) | 1 | 1.75 (0.04 - 45.95) | 0 | 0.00 (0.00 - 0.00) |
| Tello | 0 | 0.00 (0.00 - 0.00) | 1 | 1.38 (0.04 - 90.77) | 1 | 2.48 (0.06 - 92.08) |
| Tena | 1 | 3.37 (0.09 - 227.27) | 1 | 14.41 (0.36 - 389.42) | 0 | 0.00 (0.00 - 0.00) |
| Tenjo | 1 | 13.77 (0.35 - 80.88) | 0 | 0.00 (0.00 - 0.00) | 0 | 0.00 (0.00 - 0.00) |
| Teorama | 1 | 21.93 (0.56 - 130.03) | 0 | 0.00 (0.00 - 0.00) | 0 | 0.00 (0.00 - 0.00) |
| Teruel | 0 | 0.00 (0.00 - 0.00) | 1 | 3.34 (0.08 - 145.32) | 0 | 0.00 (0.00 - 0.00) |
| Tibaná | 1 | 1.49 (0.04 - 111.1) | 0 | 0.00 (0.00 - 0.00) | 1 | 0.38 (0.01 - 154.41) |
| Tibasosa | 1 | 9.44 (0.24 - 95.66) | 0 | 0.00 (0.00 - 0.00) | 0 | 0.00 (0.00 - 0.00) |
| Tibú | 1 | 3.61 (0.09 - 47.1) | 1 | 1.68 (0.04 - 40.99) | 0 | 0.00 (0.00 - 0.00) |
| Tierralta | 1 | 1.27 (0.03 - 31.03) | 6 | 6.75 (1.69 - 20.97) | 0 | 0.00 (0.00 - 0.00) |
| Timaná | 1 | 3.34 (0.08 - 457.31) | 0 | 0.00 (0.00 - 0.00) | 1 | 1.84 (0.05 - 85.89) |
| Timbío | 0 | 0.00 (0.00 - 0.00) | 1 | 1.24 (0.03 - 33.89) | 1 | 1.34 (0.03 - 30.55) |
| Tinjacá | 0 | 0.00 (0.00 - 0.00) | 1 | 29.15 (0.74 - 989.24) | 0 | 0.00 (0.00 - 0.00) |
| Tipacoque | 0 | 0.00 (0.00 - 0.00) | 0 | 0.00 (0.00 - 0.00) | 2 | 39.00 (4.72 - 353.36) |
| Tiquisio | 1 | 19.35 (0.49 - 115.31) | 0 | 0.00 (0.00 - 0.00) | 0 | 0.00 (0.00 - 0.00) |
| Tocaima | 1 | 20.52 (0.52 - 111.92) | 0 | 0.00 (0.00 - 0.00) | 0 | 0.00 (0.00 - 0.00) |
| Toledo | 2 | 6.71 (0.34 - 96.6) | 0 | 0.00 (0.00 - 0.00) | 0 | 0.00 (0.00 - 0.00) |
| Tolú Viejo | 3 | 18.43 (3.67 - 67.42) | 1 | 11.25 (0.28 - 68.27) | 0 | 0.00 (0.00 - 0.00) |
| Toro | 1 | 2.09 (0.05 - 67.01) | 1 | 0.19 (0.00 - 134.44) | 1 | 12.80 (0.32 - 86.10) |
| Totoró | 0 | 0.00 (0.00 - 0.00) | 1 | 5.16 (0.13 - 62.63) | 0 | 0.00 (0.00 - 0.00) |
| Tubará | 1 | 2.89 (0.07 - 168.4) | 0 | 0.00 (0.00 - 0.00) | 0 | 0.00 (0.00 - 0.00) |
| Tuchín | 0 | 0.00 (0.00 - 0.00) | 2 | 33.72 (1.27 - 125.62) | 1 | 0.84 (0.02 - 31.21) |
| Tuluá | 14 | 4.13 (1.87 - 11.15) | 7 | 4.59 (1.46 - 11.04) | 14 | 15.45 (6.38 - 29.82) |
| Tunja | 17 | 107.23 (39.53 - 210.42) | 2 | 1.15 (0.14 - 5.43) | 4 | 0.40 (0.09 - 16.01) |
| Túquerres | 1 | 5.91 (0.15 - 31.36) | 1 | 0.79 (0.02 - 31.52) | 0 | 0.00 (0.00 - 0.00) |
| Turbaco | 5 | 3.49 (0.62 - 20.81) | 1 | 0.64 (0.02 - 11.99) | 4 | 7.14 (1.73 - 26.05) |
| Turbaná | 1 | 31.77 (0.8 - 138.24) | 0 | 0.00 (0.00 - 0.00) | 1 | 12.08 (0.31 - 93.8) |
| Turbo | 4 | 9 (2.15 - 25.25) | 5 | 3.32 (1.04 - 10.18) | 4 | 64.62 (3.12 - 224.15) |
| Turmequé | 0 | 0.00 (0.00 - 0.00) | 0 | 0.00 (0.00 - 0.00) | 1 | 9.95 (0.25 - 150.72) |
| Tuta | 0 | 0.00 (0.00 - 0.00) | 0 | 0.00 (0.00 - 0.00) | 1 | 0.46 (0.01 - 78.78) |
| Ubaque | 0 | 0.00 (0.00 - 0.00) | 0 | 0.00 (0.00 - 0.00) | 1 | 13.24 (0.34 - 163.44) |
| Uramita | 0 | 0.00 (0.00 - 0.00) | 1 | 35.55 (0.90 - 219.92) | 0 | 0.00 (0.00 - 0.00) |
| Uribia | 3 | 1.24 (0.21 - 5.83) | 4 | 3.87 (0.76 - 12.76) | 0 | 0.00 (0.00 - 0.00) |
| Urrao | 1 | 0.08 (0.00 - 40.33) | 2 | 6.43 (0.37 - 28.63) | 0 | 0.00 (0.00 - 0.00) |
| Valdivia | 1 | 3.03 (0.08 - 47.68) | 0 | 0.00 (0.00 - 0.00) | 0 | 0.00 (0.00 - 0.00) |
| Valencia | 0 | 0.00 (0.00 - 0.00) | 0 | 0.00 (0.00 - 0.00) | 1 | 3.76 (0.10 - 33.83) |
| Valledupar | 26 | 5.09 (3.06 - 8.33) | 15 | 10.23 (4.18 - 19.4) | 23 | 12.74 (6.70 - 21.33) |
| Venecia | 1 | 15.36 (0.39 - 301.01) | 0 | 0.00 (0.00 - 0.00) | 0 | 0.00 (0.00 - 0.00) |
| Venecia | 1 | 0.69 (0.02 - 88.75) | 0 | 0.00 (0.00 - 0.00) | 0 | 0.00 (0.00 - 0.00) |
| Vianí | 1 | 0.82 (0.02 - 322.98) | 0 | 0.00 (0.00 - 0.00) | 0 | 0.00 (0.00 - 0.00) |
| Victoria | 0 | 0.00 (0.00 - 0.00) | 1 | 9.85 (0.25 - 201.05) | 0 | 0.00 (0.00 - 0.00) |
| Vijes | 1 | 0.61 (0.02 - 151.47) | 0 | 0.00 (0.00 - 0.00) | 0 | 0.00 (0.00 - 0.00) |
| Villa Caro | 1 | 21.38 (0.54 - 276.17) | 0 | 0.00 (0.00 - 0.00) | 0 | 0.00 (0.00 - 0.00) |
| Villa de Leyva | 0 | 0.00 (0.00 - 0.00) | 1 | 11.65 (0.29 - 69.05) | 0 | 0.00 (0.00 - 0.00) |
| Villa de San Diego de Ubaté | 2 | 2.90 (0.35 - 49.92) | 0 | 0.00 (0.00 - 0.00) | 0 | 0.00 (0.00 - 0.00) |
| Villa del Rosario | 1 | 3.81 (0.10 - 22.71) | 1 | 1.30 (0.03 - 12.63) | 1 | 1.88 (0.05 - 12.47) |
| Villamaría | 2 | 30.83 (1.21 - 112.6) | 0 | 0.00 (0.00 - 0.00) | 1 | 0.83 (0.02 - 18.18) |
| Villanueva | 2 | 18.25 (1.38 - 77.33) | 0 | 0.00 (0.00 - 0.00) | 0 | 0.00 (0.00 - 0.00) |
| Villanueva | 1 | 0.70 (0.02 - 41.71) | 0 | 0.00 (0.00 - 0.00) | 0 | 0.00 (0.00 - 0.00) |
| Villavicencio | 25 | 14.59 (7.77 - 24.16) | 13 | 2.82 (0.48 - 7.79) | 12 | 4.24 (2.10 - 7.75) |
| Villavieja | 0 | 0.00 (0.00 - 0.00) | 0 | 0.00 (0.00 - 0.00) | 1 | 45.63 (1.16 - 227.97) |
| Yacopí | 0 | 0.00 (0.00 - 0.00) | 1 | 0.96 (0.02 - 144.05) | 1 | 0.82 (0.02 - 109.06) |
| Yacuanquer | 1 | 1.45 (0.04 - 124.49) | 0 | 0.00 (0.00 - 0.00) | 0 | 0.00 (0.00 - 0.00) |
| Yaguará | 1 | 9.63 (0.24 - 232.18) | 0 | 0.00 (0.00 - 0.00) | 0 | 0.00 (0.00 - 0.00) |
| Yalí | 0 | 0.00 (0.00 - 0.00) | 0 | 0.00 (0.00 - 0.00) | 1 | 8.28 (0.21 - 118.17) |
| Yarumal | 1 | 4.33 (0.11 - 28.75) | 0 | 0.00 (0.00 - 0.00) | 1 | 1.64 (0.04 - 29.49) |
| Yolombó | 0 | 0.00 (0.00 - 0.00) | 1 | 7.24 (0.18 - 51.60) | 0 | 0.00 (0.00 - 0.00) |
| Yopal | 3 | 3.13 (0.45 - 24.39) | 5 | 12.04 (3.35 - 28.26) | 1 | 1.67 (0.04 - 13.43) |
| Yumbo | 5 | 0.74 (0.19 - 15.30) | 2 | 8.28 (0.29 - 31.55) | 4 | 0.06 (0.01 - 12.24) |
| Zambrano | 2 | 4.95 (0.52 - 121.62) | 0 | 0.00 (0.00 - 0.00) | 0 | 0.00 (0.00 - 0.00) |
| Zapayán | 1 | 107.82 (2.73 - 427.03) | 0 | 0.00 (0.00 - 0.00) | 0 | 0.00 (0.00 - 0.00) |
| Zaragoza | 0 | 0.00 (0.00 - 0.00) | 1 | 4.30 (0.11 - 81.31) | 0 | 0.00 (0.00 - 0.00) |
| Zarzal | 2 | 4.62 (0.17 - 30.92) | 0 | 0.00 (0.00 - 0.00) | 1 | 0.75 (0.02 - 32.26) |
| Zetaquira | 0 | 0.00 (0.00 - 0.00) | 0 | 0.00 (0.00 - 0.00) | 1 | 2.07 (0.05 - 288.12) |
| Zipaquirá | 1 | 7.22 (0.18 - 28.64) | 0 | 0.00 (0.00 - 0.00) | 7 | 68.35 (11.78 - 177.76) |
| Zona Bananera | 1 | 0.40 (0.01 - 16.41) | 2 | 5.41 (0.20 - 39.24) | 0 | 0.00 (0.00 - 0.00) |
| **National** | **2,454** | **10.48 (10.07 - 10.91)** | **1,021** | **4.31 (4.05 - 4.58)** | **1,641** | **7.58 (7.22 - 7.96)** |

^1^ All-cause mortality rates are age-standardized per 100.000 population. They were estimated including only invasive cases. The municipalities in which there were no deaths in any of this types of cancer were excluded.
