# Supplementary figures and images for: Patterns of breast, prostate and cervical cancer incidence and mortality in Colombia: an administrative registry data analysis
Source: BMC Cancer. 2020 Nov 11;20:1097. doi: 10.1186/s12885-020-07611-9 (PMC7661250; doi:10.1186/s12885-020-07611-9)

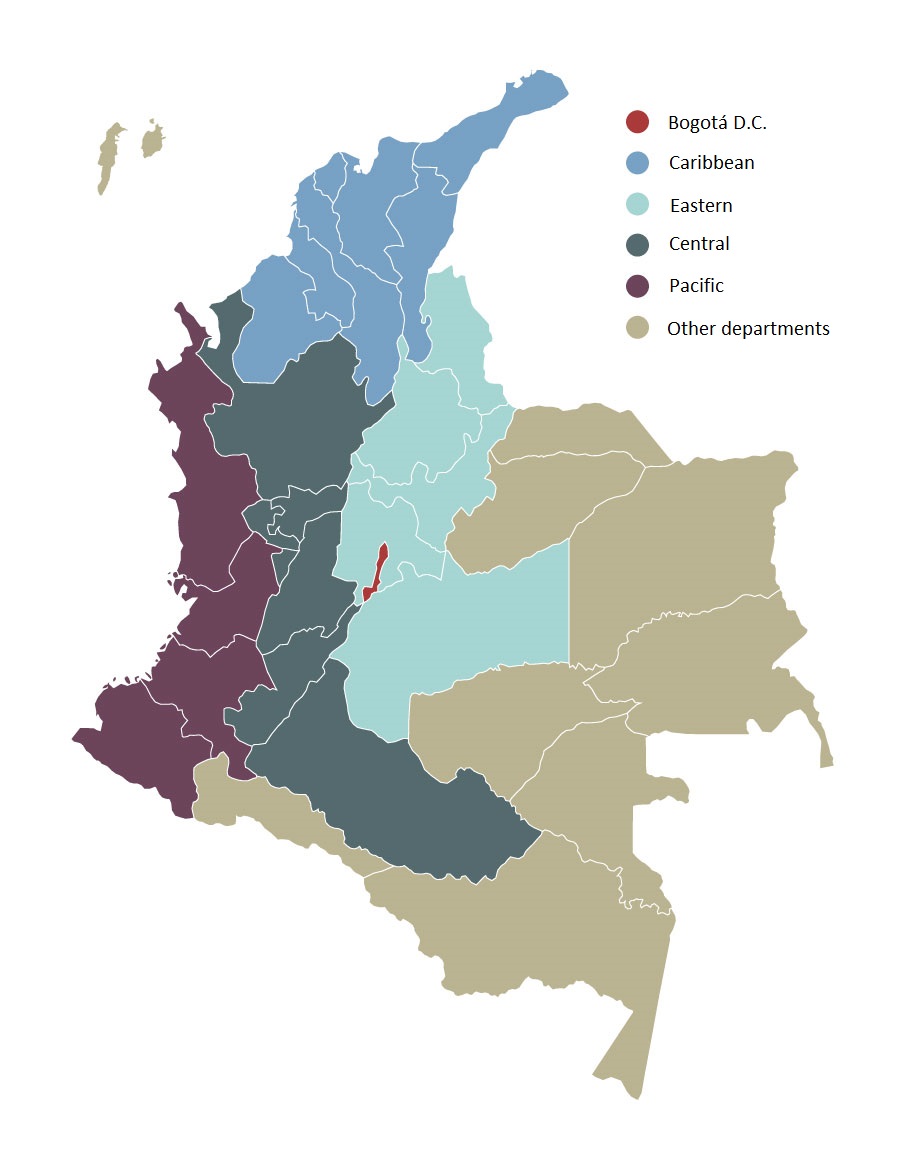

Supplement: Supplementary file 1 — Additional file 1: Figure S1. Socioeconomic distribution of regions in Colombia, 20181. Table S1. Incidence rates for breast, prostate and cervical cancer by municipalities, Colombia 20181. Table S2. Mortality rates for breast. Prostate and cervical cancer by municipalities. Colombia 20181. [file 12885_2020_7611_MOESM1_ESM.zip › Fig 1_Sup materialR3.jpg]
